# CD169+ Macrophages Mediate the Immune Response of Allergic Rhinitis Through the Keap1/Nrf2/HO‐1 Axis

**DOI:** 10.1002/advs.202309331

**Published:** 2024-10-22

**Authors:** Wenwen Qi, Chengcheng Liu, Lei Shi, Hui Li, Xiaozhi Hou, Hongjie Du, Luqiu Chen, Xiaochen Gao, Xue Cao, Na Guo, Yuhan Dong, Chengzhilin Li, Fanyu Yuan, Zhenxiao Teng, Houyang Hu, Fangyuan Zhu, Xuanchen Zhou, Lulu Guo, Miaoqing Zhao, Ming Xia

**Affiliations:** ^1^ Department of Otolaryngology Shandong Provincial Hospital Affiliated to Shandong First Medical University Jinan China; ^2^ Department of Central Laboratory Shandong Provincial Hospital Affiliated to Shandong First Medical University Jinan China; ^3^ Medical Science and Technology Innovation Center Shandong First Medical University & Shandong Academy of Medical Sciences Jinan China; ^4^ Department of Pediatric Surgery Qilu Hospital Cheeloo College of Medicine Shandong University Jinan Shandong 250012 China; ^5^ Department of Otolaryngology Shandong Provincial Hospital Shandong University Jinan China; ^6^ Advanced Medical Research Institute Cheeloo College of Medicine NHC Key Laboratory of Otorhinolaryngology Shandong University Jinan China; ^7^ Department of Pathology Shandong Cancer Hospital and Institute Shandong First Medical University and Shandong Academy of Medical Sciences Jinan China; ^8^ NHC Key Laboratory of Otorhinolaryngology Jinan China

**Keywords:** allergic rhinitis, CD169+ macrophages, immune response, Keap1/Nrf2/HO‐1 axis, ROS

## Abstract

CD169+ macrophages are a newly defined macrophage subpopulation that can recognize and bind with other cells through related ligands, playing an essential role in antigen presentation and immune tolerance. However, its role in Allergic Rhinitis (AR) is still unclear. To investigate the characteristics of CD169+ macrophages in AR, this work first detects their expression patterns in the nasal mucosa of clinical patients. These results show a significant increase in CD169+ macrophages in the nasal mucosa of patients with AR. Subsequently, this work establishes an animal AR model using CD169 transgenic mice and compared the advantages of the two models. Moreover, this work also demonstrates the effects of CD169 knockout on eosinophils, Th cells, Treg cells, and the migration of dendritic cells (DCs). In addition, this metabolomic data shows that CD169+ macrophages can upregulate alanine production and increase reactive oxygen species (ROS) levels. This process may be mediated through the Keap1/Nrf2/HO‐1 signaling pathway. In addition, this work also finds that SLC38A2 plays an essential role in the process of CD169+ macrophages promoting alanine uptake by DCs. This study confirms that CD169+ macrophages can upregulate their internal alanine production and increase ROS levels through the Keap1/Nrf2/HO‐1 axis, playing an irreplaceable role in AR.

## Graphical abstract

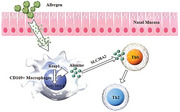

## 1 Introduction

In recent decades, the prevalence of allergic diseases has witnessed a significant increase, coinciding with the rapid advancement of society and the deterioration of the environment. This escalation poses a substantial economic burden on public health. Notably, our comprehension of the underlying mechanisms behind allergic diseases has progressed considerably recently. It is well established that the onset and progression of allergic diseases are intricately linked to alterations in the immune system. Allergic Rhinitis (AR) not only directly impacts the patient's health but also induces sleep disturbances and diminishes overall work and study efficiency. AR is recognized as a complex interplay between environmental exposures and genetic predisposition. This condition has evolved into a global concern, notably due to its escalating incidence in recent years.^[^ ^1^ ^]^

The foundation of AR rests upon the imbalance within the immune network of helper T cells (Th cells). As T cells can solely recognize antigenic peptides obtained after processing by antigen‐presenting cells, such as dendritic cells (DC), these cells assume a pivotal role. Dendritic cells serve as the principal antigen‐presenting cells in the human body. Their functions encompass the ingestion and processing of allergens, secretion of chemokines, and upregulation of surface co‐stimulatory molecules. Subsequently, dendritic cells present antigenic peptides to initial T cells, driving their differentiation into Th2 cells. Th2 cells, crucial components of humoral immunity, orchestrate the regulation of type 2 immune responses, primarily through the secretion of cytokines like interleukin‐4 (IL‐4), IL‐5, and IL‐13.^[^ ^2^ ^]^ Recent years have yielded substantial progress in the understanding of allergic disease pathogenesis. The atypical activation of Th2 cells in mice and humans, mediated by DC, results from a blend of genetic factors and external environmental influences. These external factors encompass household dust mites, bacteria, and specific allergens.^[^ ^3^ ^]^ Research has underscored the significant role that dendritic cells play in regulating nasal mucosal immunity and influencing CD4+ T cell polarization. Current treatments for AR, encompassing glucocorticoids, immunotherapy, and anti‐IgE therapy, all function through the regulation of DC activity.^[^ ^4^ ^]^ Given dendritic cells’ involvement in initiating allergic reactions and their critical role in allergic diseases, research in this realm has garnered considerable attention, albeit with some gaps in the upstream investigation of dendritic cells.

Macrophages constitute a recently identified group of immune cells known for their high plasticity and multifaceted roles in various physiological processes. These roles extend beyond their well‐documented function in combating bacterial infections to encompass participation in immune responses and tissue repair during sterile inflammation. Presently, macrophages are classified into four main categories: classically activated macrophages (M1 macrophages), alternatively activated macrophages (M2 macrophages), tumor‐associated macrophages (TAM), and CD169+ macrophages. They are ubiquitous throughout the body's diverse tissues and organs. Surface markers help distinguish macrophage subgroups,^[^ ^5^ ^]^ with type CD80, CD68, CD86, and MHCII characterizing M1 macrophages, which secrete inflammatory factors such as IL‐12, TNF, and IL‐18. M1 macrophages are adept at phagocytizing pathogens and eliminating intracellular bacteria. M2 macrophages, on the other hand, are characterized by surface markers CD163 and CD206 and serve diverse roles beyond allergic diseases. CD169, also known as either sialoadhesion (Sn) or sialic acid‐binding immunoglobulin‐like lectin 1 (Siglec1), is a specific marker for tissue‐resident macrophages.^[^ ^6^, ^7^, ^8^, ^9^, ^10^, ^11^, ^12^ ^]^ Macrophages contribute significantly to tissue remodeling, angiogenesis, and combating parasitic infections.

Our research has demonstrated an elevated presence of CD169+ macrophages in the nasal mucosal epithelium of individuals suffering from AR. Furthermore, our work with AR mouse models has unveiled the critical role played by CD169+ macrophages in promoting dendritic cell activation. Consequently, our current study seeks to elucidate the precise phenotype of CD169+ macrophages in the nasal mucosa and unravel the mechanisms through which they contribute to the pathogenesis of AR. Our investigation extends to comprehending the broader implications on various immune cells and cytokines within the AR pathway. Ultimately, we aim to identify novel targets for drug interventions to prevent and treat AR.

## 2 Results

### 2.1 CD169 was Highly Expressed in Nasal Mucosal Epithelial Cells and Nasal Lavage Fluid in Individuals Affected by AR

**Figure 1:**
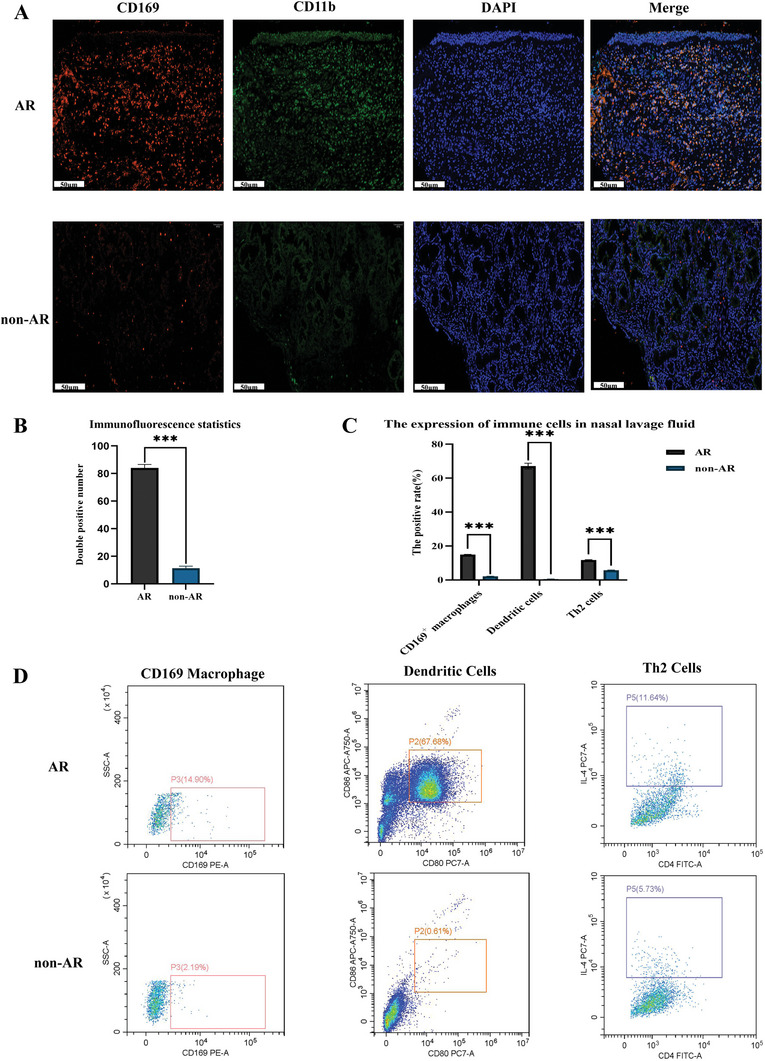
Nasal mucosal epithelial immunofluorescence and flow cytometry of nasal lavage fluid in patients with allergic rhinitis and non‐allergic rhinitis. A) Nasal mucosal epithelial immunofluorescence. Red is CD169 staining, green is CD11b staining, blue is DAPI staining, scale bar = 50 µm. B) The statistical analysis of the fluorescent intensity. The fluorescent intensity was quantified using Image J. C) Statistical diagram of flow cytometry results of nasal lavage fluid. The bar graphs and the table show quantification of the results, with each value representing the mean ± SEM of three independent experiments. Statistical significance is shown using the unpaired two‐tailed Student *t*‐test analysis; ***p < 0.001. D) CD169+ macrophages, dendritic cells and flow cytometry were detected in nasal lavage fluid of patients.

We selected pathological sections of inferior turbinate mucosa and nasal lavage fluid by normal saline of 20 hospitalized patients with allergic rhinitis as the experimental group, and selected 20 cases of inferior turbinate mucosa of patients with simple non‐allergic diseases such as nasal septum deviation as the control group hospitalized in the department of otorhinolaryngology head and neck surgery, Shandong Provincial Hospital Affiliated to Shandong First Medical University from January 1 from 2019 to December 31, 2019. Immunofluorescence analysis was conducted on the pathological sections, where cell nuclei stained blue using DAPI, CD11b was represented in green, and CD169 was depicted in red. **Figure** **1** illustrates that CD169 expression is substantially elevated in the nasal mucosa of AR patients. The predominant localization of CD169 clustered around blood vessels and lymphatic structures.

### 2.2 Knockout of CD169+ Macrophages can Alleviate Allergic Behavior in Mice

**Figure 2:**
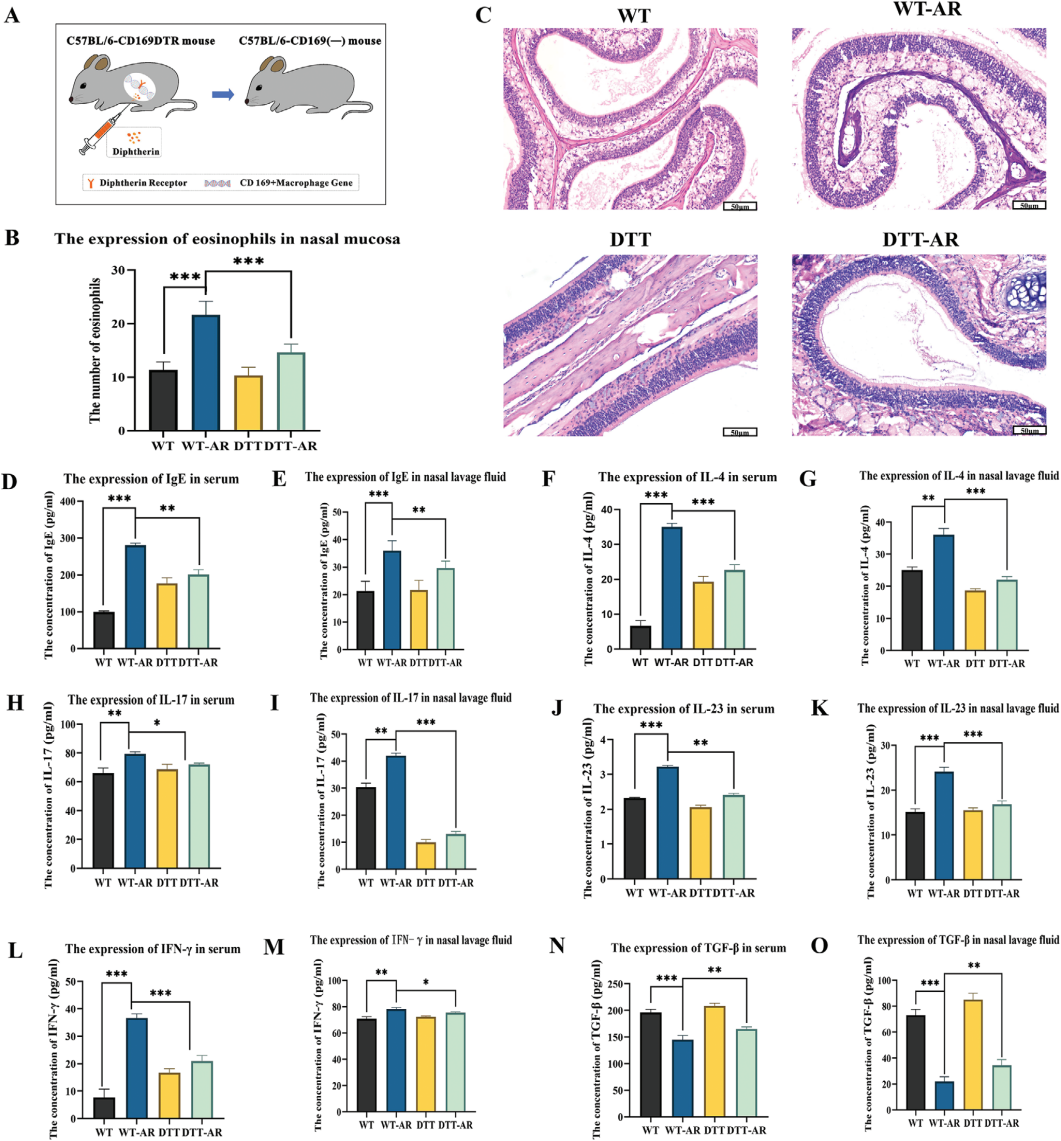
Detection of nasal mucosa and lavage fluid in mice. A) The construction model of transgenic mice. The diphtheria toxin receptor was inserted into a fragment of the mouse gene expressing CD169+ macrophages. When given intraperitoneal injection of diphtheria toxin, the mouse could selectively knock out CD169+ macrophages. B) Statistical map of eosinophils in nasal mucosa of mice. C) HE staining of mouse nasal mucosa, scale bar = 50 µm. D) Serum IgE content in mice. E) IgE content in nasal lavage fluid of mice. F) Serum IL‐4 content in mice. G) IL‐4 content in nasal lavage fluid of mice. H) Serum IL‐17 content in mice. I) IL‐17 content in nasal lavage fluid of mice. J) Serum IL‐23 content in mice. K) IL‐23 content in nasal lavage fluid of mice. L) Serum IFN‐γ content in mice. M) IFN‐γ content in nasal lavage fluid of mice. N) Serum TGF‐β content in mice. O) TGF‐β content in nasal lavage fluid of mice. All these results showed that the model was successfully constructed, and the mouse allergy indexes significantly decreased after CD169+ macrophages were knocked out. The bar graphs show the quantification of the results, with each value representing the mean ± SEM of three independent experiments. Statistical significance is shown using the unpaired two‐tailed Student's *t*‐test; *p < 0.05; **p < 0.01; ***p < 0.001.

**Figure** **2**A depicts the experimental setup involving four distinct groups: The WT group consisted of standard C57 mice, the WT‐AR group comprised normal C57 mice with an induced AR model by ovalbumin (OVA) as reported in the literature^[^ ^13^, ^14^, ^15^ ^]^ and our article published earlier,^[^ ^16^, ^17^ ^]^ the diphtheria toxin receptor (DTT) group involved transgenic mice treated with diphtheria toxin, and the DTT‐AR group consisted of transgenic mice treated with diphtheria toxin and subjected to the AR model.^[^ ^18^, ^19^, ^20^, ^21^, ^22^, ^23^ ^]^ During the experiment, the number of instances in which mice scratched their noses or sneezed within 10 min was documented. **Tables** **1** and **2** summarizes the results, revealing that the WT‐AR group exhibited a significantly higher frequency of nose scratching and sneezing than the WT group, signifying the effective establishment of the AR model. Notably, the table suggests a substantial reduction in allergic behaviors when CD169+ macrophages were knocked out, implicating these macrophages in initiating allergic responses. Conversely, in non‐AR mice, knocking out CD169+ macrophages did not result in any significant difference in allergic behaviors compared to the WT group, indicating that CD169+ macrophage knockout had no discernible impact on their behavior.

**Table 1:** Number of nose scratches(n = 11, $\bar{X}$ ± S).

Group	Day 0	Day 5	Day 15	Day 20	Day 25	Day 30	Day 35	Day 40
WT	9.4 ± 1.31	9.3 ± 1.14	9.5 ± 2.19	8.9 ± 1.93	9.5 ± 2.13	7.98 ± 1.31	9.41 ± 1.43	8.34 ± 3.13
WT‐AR	8.7 ± 1.21	9.4 ± 1.22	11.9 ± 1.22	12.43 ± 3.12	11.54 ± 2.15	13.43 ± 2.62	18.41 ± 1.53	18.21 ± 2.14
DTT	9.2 + 1.24	8.4 ± 1.35	8.3 ± 1.98	7.4 ± 5.1	8.43 ± 2.51	8.42 ± 1.42	9.13 ± 1.45	9.87 ± 2.41
DTT‐AR	9.6 ± 1.12	9.1 ± 0.81	9.3 ± 1.32	9.23 ± 2.3	9.87 ± 1.23	8.46 ± 2.14	9.52 ± 3.13	10.41 ± 1.56

**Table 2:** Number of sneezes(n = 11, $\bar{X}$ ± S).

Group	Day 0	Day 5	Day 15	Day 20	Day 25	Day 30	Day 35	Day 40
WT	5.1 ± 0.56	5.6 ± 3.16	6.0 ± 2.16	4.24 ± 1.21	5.75 ± 1.24	5.31 ± 1.53	6.31 ± 1.52	5.85 ± 2.41
WT‐AR	4.5 ± 2.78	5.3 ± 1.08	6.9 ± 3.78	7.42 ± 2.51	9.52 ± 1.34	9.87 ± 1.41	10.52 ± 1.34	11.53 ± 2.13
DTT	5.2 ± 1.31	5.5 ± 2.15	5.8 ± 1.54	4.62 ± 1.34	5.24 ± 1.63	6.34 ± 2.52	5.21 ± 1.52	6.53 ± 1.53
DTT‐AR	5.5 ± 2.19	5.1 ± 3.09	5.7 ± 3.09	4.21 ± 1.53	5.21 ± 1.43	6.24 ± 1.24	6.41 ± 1.53	6.42 ± 2.63

### 2.3 Knockout of CD169+ Macrophages Reduced the Number of Eosinophils and the Expression of Related Cytokines in AR Mice

**Figure 3:**
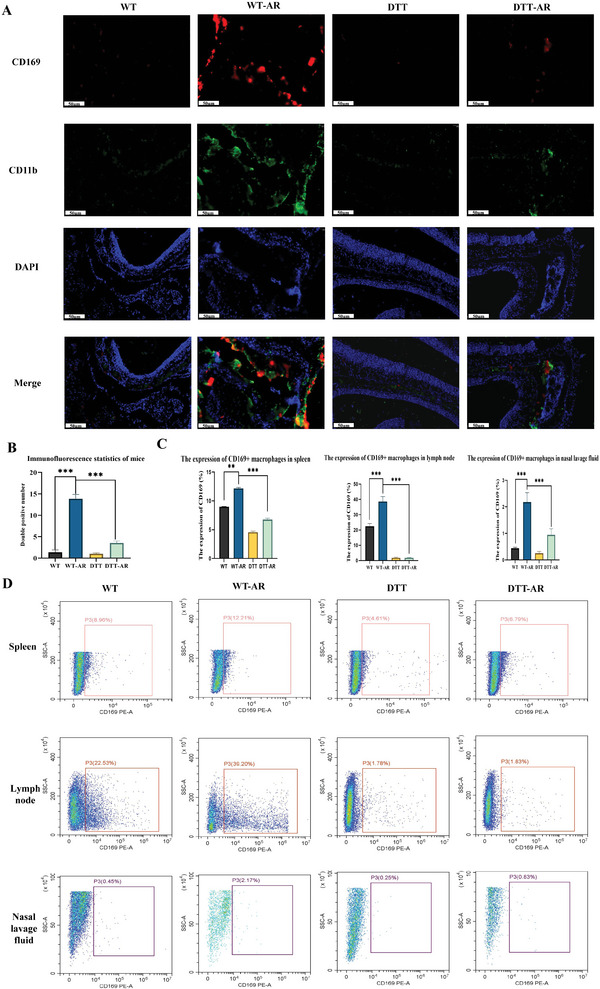
Immunofluorescence and flow cytometry were used to detect the expression of CD169+ macrophages in the spleen, lymph nodes, and nasal lavage fluid of mice. A) Nasal mucosal epithelial immunofluorescence and the statistical analysis of the fluorescent intensity. Red is CD169 staining, green is CD11b staining, blue is DAPI staining, scale bar = 50 µm. B) The statistical analysis of the fluorescent intensity. The fluorescent intensity was quantified using Image J. The bar graphs and the table show quantification of the results, with each value representing the mean ± SEM of three independent experiments. Statistical significance is shown using the unpaired two‐tailed Student's *t*‐test; ***p < 0.001. C) Statistical diagram of flow cytometry results of spleen, lymph node and nasal lavage fluid. The bar graphs and the table show quantification of the results, with each value representing the mean ± SEM of three independent experiments. Statistical significance is shown using the Student *t*‐test analysis; **p < 0.01; ***p < 0.001. D) Flow cytometry was used to detect the expression of CD169 macrophages in the spleen, lymph nodes and nasal lavage fluid of mice.

Following the euthanasia of the mice, their nasal mucosa (head) was subjected to H&E staining, and the eosinophil count was conducted using an optical microscope. As illustrated in Figure 2B,C, knocking out CD169+ macrophages resulted in a substantial reduction in the number of eosinophils in the nasal mucosa of AR mice. Notably, the DTT‐AR group exhibited a lower eosinophil count than the WT‐AR group. However, there was no significant alteration in eosinophil levels in non‐AR mice upon CD169+ macrophage knockout. Blood samples were collected from the eyeball and allowed to sit at 4 °C for 24 h, after which serum was isolated. Subsequent low‐temperature centrifugation of the serum allowed for IgE ELISA detection. Enzymograph processing of the ELISA results, as depicted in **Figure** **3**D, revealed a significant reduction in serum IgE concentration in the DTT‐AR group following CD169+ macrophage removal compared to the WT‐AR group. In addition, following euthanasia, the nasal cavities of mice were rinsed with PBS, and the nasal lavage solution was collected.^[^ ^24^ ^]^ After low‐temperature centrifugation, the nasal lavage solution was subjected to IgE ELISA detection (Figure 3D). Notably, CD169+ macrophage elimination led to a substantially lower IgE concentration in the nasal lavage solution of the DTT‐AR group compared to the WT‐AR group, consistent with the serum ELISA findings. Following the euthanasia of the mice, the nasal lavage fluid and serum of the mice were used for ELISA detection of inflammation‐related cytokines. As illustrated in Figure 2, the expression levels of IL‐23, IL‐17, IL‐4, and IFN‐γ in nasal lavage fluid and serum were all elevated following sensitization. At the same time, the above cytokines were lowered to varying degrees after the knockout of CD169+ macrophages. In contrast, TGF‐β levels suggest that other macrophages may possess compensatory effects.

### 2.4 AR Impacted the Expression of CD169+ Macrophages and Knockout of CD169+ Macrophages Inhibited the Differentiation of Th0 Cells into Th17 and Th2 Cells

**Figure 4:**
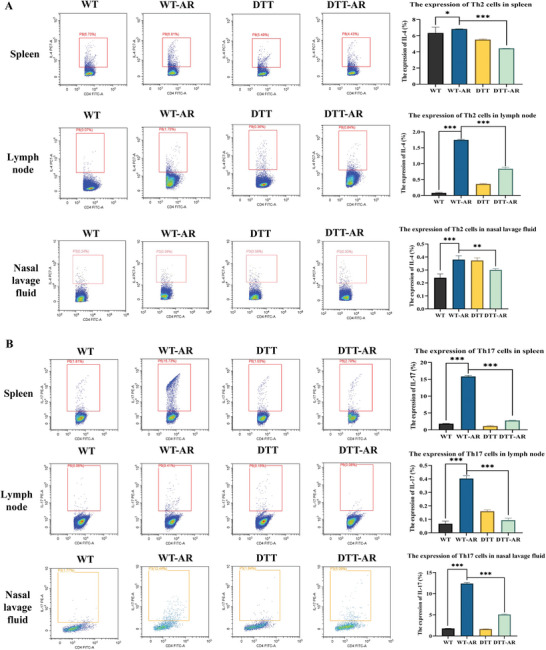
Knockout of CD169+ macrophages inhibited the differentiation of Th0 cells into Th17 and Th2 cells. A) Flow cytometry was used to detect the expression of Th2 in the spleen, lymph nodes, and nasal lavage fluid of mice. B) Flow cytometry was used to detect the expression of Th17 in the spleen, lymph nodes, and nasal lavage fluid of mice. The bar graphs show quantification of the results, with each value representing the mean ± SEM of three independent experiments. Statistical significance is shown using the unpaired two‐tailed Student's *t*‐test; *p < 0.05; **p < 0.01; ***p < 0.001.

Following the euthanasia of the mice, flow detection of CD169+ macrophages was conducted using nasal lavage fluid, neck, mesenteric lymph nodes, and spleen. Because the allergens were mainly drained to the deep and shallow lymph nodes of the neck through the nasal mucosa, throat mucosa, and bronchial mucosa of the upper and lower respiratory tract, we selected cervical lymph nodes for the experiment.^[^ ^25^ ^]^ As depicted in Figures 3 and **4**, it was observed that the expression of CD169+ macrophages in the spleen, lymph nodes, and nasal lavage fluid of mice subjected to diphtheria toxin treatment decreased in both the DTT group and DTT‐AR group, indicating the successful knockout of CD169+ macrophages in transgenic mice. However, CD169+ macrophages in nasal lavage fluid and spleen showed a slight increase in the DTT‐AR group, suggesting that AR influenced the expression of CD169+ macrophages. Upon euthanasia of the mice, relevant indexes for Th2 (CD4, IL‐4) and Th17 (CD4, IL‐17) cells were stained using nasal lavage fluid, cervical, mesenteric lymph nodes, and spleen, followed by analysis through flow cytometry. As displayed in Figure 4, the proportion of Th2 and Th17 cells in nasal lavage fluid, lymph nodes, and spleen increased after sensitization, irrespective of diphtheria toxin usage. However, following diphtheria toxin treatment, the proportion of Th2 and Th17 cells in the nasal lavage fluid, lymph nodes, and spleen decreased, regardless of sensitization.

### 2.5 Knockout of CD169+ Macrophages Inhibited the Mature Proportion of Dendritic Cells and Increased the Proportion of Treg Cells in AR Mice

**Figure 5:**
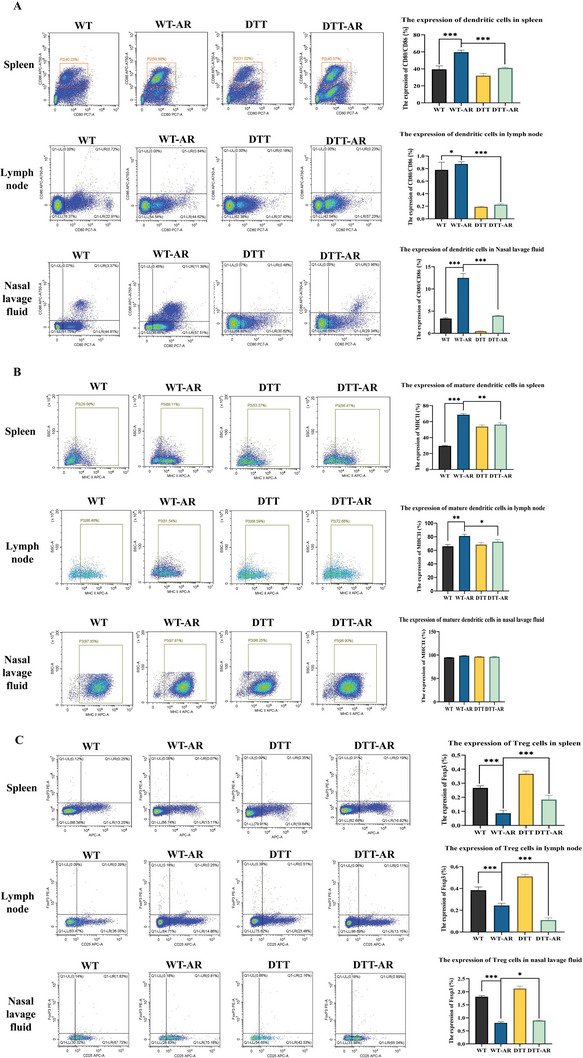
The knockout of CD169+ macrophage inhibited the expression of dendritic cells and Treg cells in mice. A) Flow cytometry was used to detect the expression of dendritic cells in the spleen, lymph nodes, and nasal lavage fluid of mice. B) Flow cytometry was used to detect the expression of mature dendritic cells in the spleen, lymph nodes, and nasal lavage fluid of mice. C) Flow cytometry was used to detect the expression of Treg in the spleen, lymph nodes, and nasal lavage fluid of mice. The bar graphs show quantification of the results, with each value representing the mean ± SEM of three independent experiments. Statistical significance is shown using the unpaired two‐tailed Student's *t*‐test; *p < 0.05; **p < 0.01; ***p < 0.001.

Following euthanasia of the mice, dendritic cells (CD80, CD86) and Treg cells (CD25, Foxp3) were stained using nasal irrigation fluid, cervical, mesenteric lymph nodes, and spleen.^[^ ^26^ ^]^ As depicted in **Figure** **5**, it was determined that following sensitization, the total number of dendritic cells, as well as the proportion of mature cells, was increased. In the spleen, lymph nodes, and nasal lavage fluid of mice treated with diphtheria toxin, the total count of DC in both the DTT group and the DTT‐AR group decreased relative to the WT group. While the total number of DC cells experienced a slight increase following sensitization, it decreased significantly compared to the WT‐AR group. The number of mature dendritic cells was altered according to the total number of dendritic cells (Figure 5), but not as significantly as the total number of dendritic cells. These results suggest that CD169+ macrophage knockout can significantly limit the proportion of dendritic cells and inhibit the maturation of dendritic cells within AR mice. However, the levels of Treg cells in nasal lavage fluid, lymph nodes, and spleen of mice were reduced following sensitization, irrespective of diphtheria toxin usage. Following treatment with diphtheria toxin, the proportion of Treg cells in nasal lavage fluid, lymph nodes, and spleen in mice increased regardless of sensitization.

### 2.6 CD169+ Macrophages Knocked Out Abnormal Alanine/Aspartate/Glutamate Metabolic Pathways in Mice

**Figure 6:**
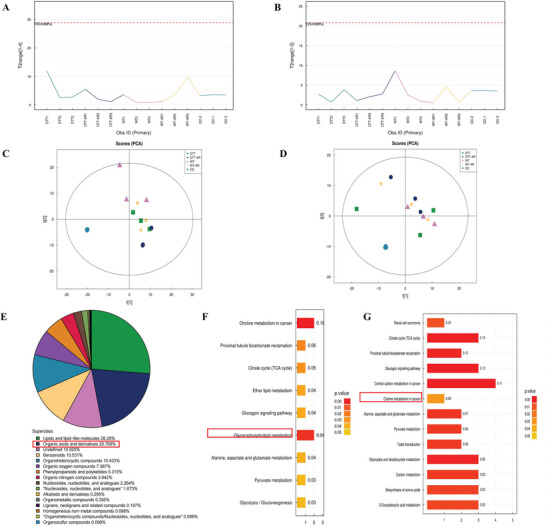
Metabolomics results of mouse nasal lavage fluid. A) Hotellings T2 diagram of the positive ion model population sample. B) Hotellings T2 diagram of the negative ion model population sample. C) PCA analysis of positive ion model population samples. D) PCA analysis of negative ion model population samples. E) The number and proportion of metabolites identified in each chemical classification. In the F and G histogram, the vertical axis represents each KEGG metabolic pathway, and the horizontal axis represents the number of differentially expressed metabolites in each KEGG metabolic pathway. Color represents the P value of enrichment analysis, and the darker the color, the smaller the P value, the more significant the enrichment degree. F) The alanine pathway was down‐regulated in WT and DTT groups. G) The downregulation of the alanine pathway was more evident in WT‐AR and DTT‐AR groups.

The comparison of the total ion chromatogram (TIC) used for quality control demonstrated that the intensity and retention times of each peak value were largely consistent. Hotelling's T2 test tests the samples through multivariate modeling and defines 95% or 99% confidence intervals, which can be used to diagnose outlier samples. The test results of Hotelling's T2 are shown in **Figure** **6**. In the figure, the horizontal coordinate represents all experimental and QC samples, the vertical coordinate reflects the confidence interval, and the red line defines the 99% confidence interval range. The experimental results showed that all QC samples were within a 99% confidence interval, indicating good experiment repeatability. As indicated in Figure 6C,D, the quality control samples in both positive and negative ion modes displayed close clustering, suggesting the repeatability of the experiment.

Metabolites identified in both positive and negative ion modes were categorized and quantified based on their chemical classification and attribution information, and the distribution of various metabolites is illustrated in Figure 6E. Furthermore, the enrichment analysis function of KEGG pathways was employed to analyze the differential metabolic pathways, with the up‐regulated and down‐regulated information depicted in different colors. Changes in the alanine pathway can be observed in Figure 6.

### 2.7 CD169+ Macrophages up‐Regulate Internal Alanine Production and ROS Levels via the Keap1/Nrf2/HO‐1 Axis

**Figure 7:**
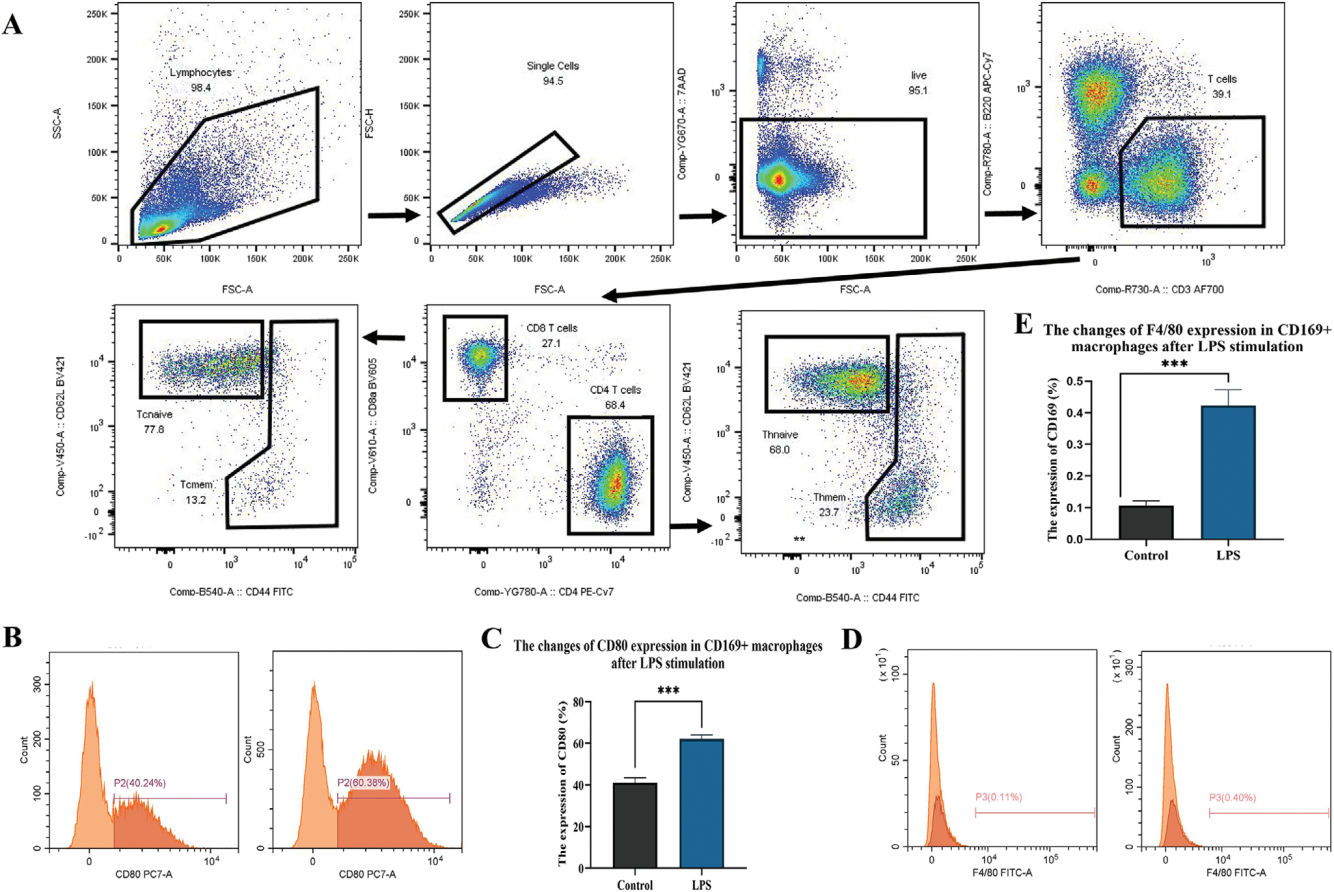
Properties of CD169+ macrophages by flow sorting. A)CD169+ macrophages from the spleen of mice in the WT group were separated and cultured by flow sorting technique. B) Lipopolysaccharide (LPS) stimulated the cell population, and the positive rates of CD80 were detected by flow cytometry. C) Statistical graph of CD80 positive rate. D) The positive rates of F4/80 were detected by flow cytometry and the statistical graph (E). The bar graphs show the quantification of the results, with each value representing the mean ± SEM of three independent experiments. Statistical significance is shown using the unpaired two‐tailed Student's *t*‐test; ***p < 0.001.

Following the death of the mice, nasal lavage solution was utilized to treat mice from the WT group, facilitating the flow sorting of CD169+ macrophages, which were subsequently isolated and cultured in vitro for 48 h. These selected CD169+ macrophages were then subjected to lipopolysaccharide (LPS) stimulation, and the phenotype changes of CD169+ macrophages were assessed using flow cytometry.^[^ ^27^, ^28^ ^]^ The results indicated an increased proportion of CD80+, but no significant change in the positive rate of F4/80, as **Figure** **7** shows.

**Figure 8:**
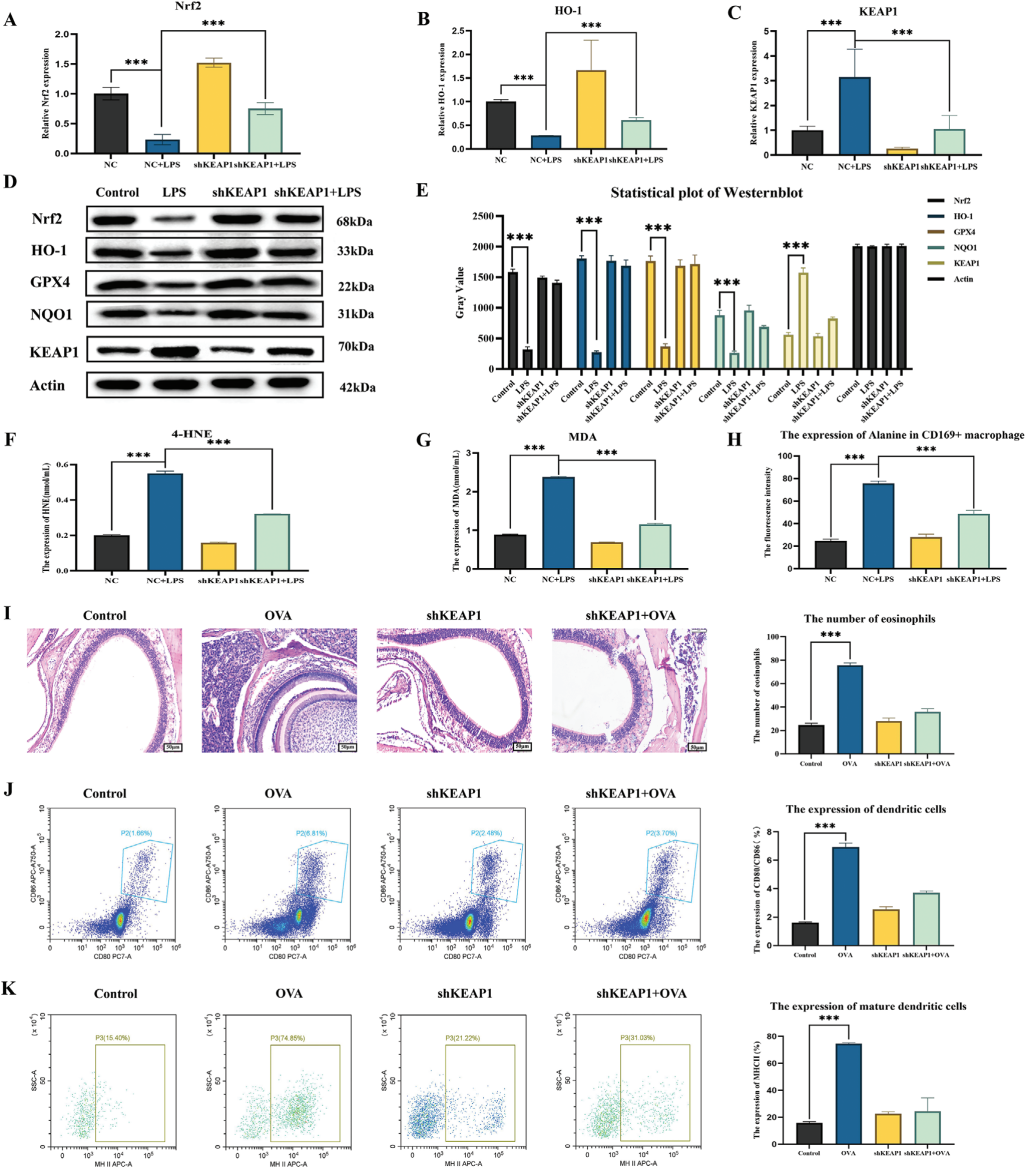
CD169+ macrophages up‐regulate their internal alanine production and ROS levels through the Keap1/Nrf2/HO‐1 axis. A) The RNA expression level of Nrf2 was detected by Q‐PCR. B) The RNA expression level of HO‐1 was detected by Q‐PCR. C) The RNA expression level of KEAP1 was detected by Q‐PCR. D) Western blot detected the Keap1/Nrf2/HO‐1 protein pathway. E) The statistical map of western blot. F) The expression level of HNE was detected by ELISA assay. G) The expression level of MDA was detected by ELISA assay. H) The expression level of alanine was detected by an alanine test kit. I) HE staining of the nasal mucosa and statistical map of eosinophilic content, scale bar = 50 µm. J) Flow cytometry of mouse dendritic cells. K) Flow cytometry of mouse dendritic cell maturity. The bar graphs show the quantification of the results, with each value representing the mean ± SEM of three independent experiments. Statistical significance is shown using the unpaired two‐tailed Student's *t*‐test; ***p < 0.001.

Western blot was used to assess the protein content expression of the Keap1/Nrf2/HO‐1 pathway in these cells (**Figure** **8**D,E), and qPCR was used to examine alterations in the mRNA levels within this pathway (Figure 8A–C). To gauge changes in oxidative stress products MDA and HNE within the cell culture medium, ELISA technology was utilized (Figure 8F,G). The findings revealed an upregulation of Nrf2 and HO‐1 expression in CD169 macrophages following LPS treatment, coupled with a decrease in Keap1 expression, reversible after transfection with shKEAP1. We constructed a mouse model with Keap1 gene knockout, and took nasal lavage fluid and nasal mucosa of mice for HE staining and flow cytometry experiments. The results showed that the knockout of keap1 could inhibit the local allergic inflammatory reaction and the maturation of dendritic cells in the nasal mucosa, as Figure 8I–K depict. These suggest that CD169 macrophages can up‐regulate the production of alanine and ROS levels within their cells through the Keap1/Nrf2/HO‐1 axis, subsequently releasing them into the nasal mucosal microenvironment.

### 2.8 SLC38A2 Inhibitors can Reverse the Promotion of CD169+ Macrophages on the Maturation and Migration of Dendritic Cells

**Figure 9:**
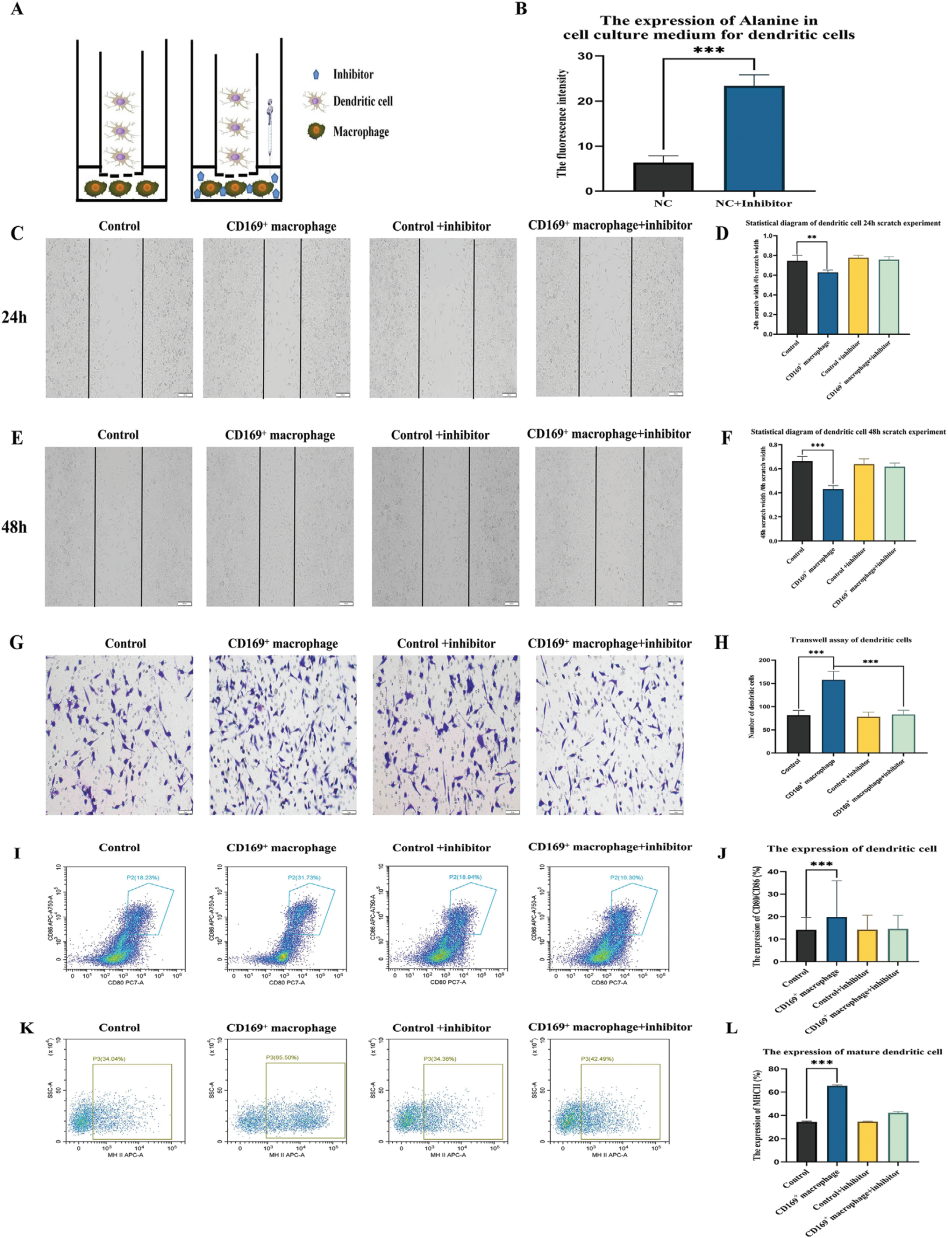
Influence of CD169+ macrophages co‐culture with dendritic cells on the migration of dendritic cells treated with SLC38A2 inhibitor. A) Co‐culture diagram. B) Detection of alanine content in co‐cultured cell lines. C) 24‐h cell scratch map of dendritic cells. D) 24‐h cell scratch statistics, scale bar = 100 µm. E) 48‐h cell scratch map of dendritic cells. F) 48‐h cell scratch statistics, scale bar = 100 µm. G) 48‐h Transwell results of dendritic cells, scale bar = 50 µm. H)Transwell experimental statistical diagram. I) Flow cytometry was used to detect the expression of dendritic cells in the nasal lavage fluid of mice. J) The statistical map of dendritic cells. K) Flow cytometry was used to detect the expression of mature dendritic cells in the nasal lavage fluid of mice. L) The statistical map of mature dendritic cells. The bar graphs show the quantification of the results, with each value representing the mean ± SEM of three independent experiments. Statistical significance is shown using the unpaired two‐tailed Student's *t*‐test; **p < 0.01; ***p < 0.001.

A co‐culture system was also devised for CD169 macrophages and dendritic cells (**Figure** **9**A), and an alanine detection kit was used to gauge changes in alanine content in the cell culture medium post‐*co*‐culture. The results demonstrated that the SLC38A2 inhibitor significantly reduced the uptake of alanine by DC (Figure 9B), as reported.^[^ ^29^, ^30^, ^31^ ^]^ Dendritic cell lines were co‐cultured alongside CD169+ macrophages from the flow sorting, and dendritic cells were subjected to cell scratch and transwell experiments. The migration ability of dendritic cells was significantly improved following the addition of CD169+ macrophages, however, the migration ability was limited after the addition of SLC38A2 inhibitors, indicating that SLC38A2 inhibitors could reverse the migration of dendritic cells caused by CD169+ macrophages (Figure 9). The co‐cultured dendritic cells were then examined by flow cytometry, as illustrated in Figure 9I,K, indicating that SLC38A2 inhibitors could reverse the maturation of dendritic cells caused by CD169+ macrophages.

### 2.9 Inhibition of the SLC38A2 Pathway can Inhibit the Expression of Mouse CD169+ Macrophages, the Maturation of Mouse Dendritic Cells, and Impact the Expression of Mouse Th Cells

**Figure 10:**
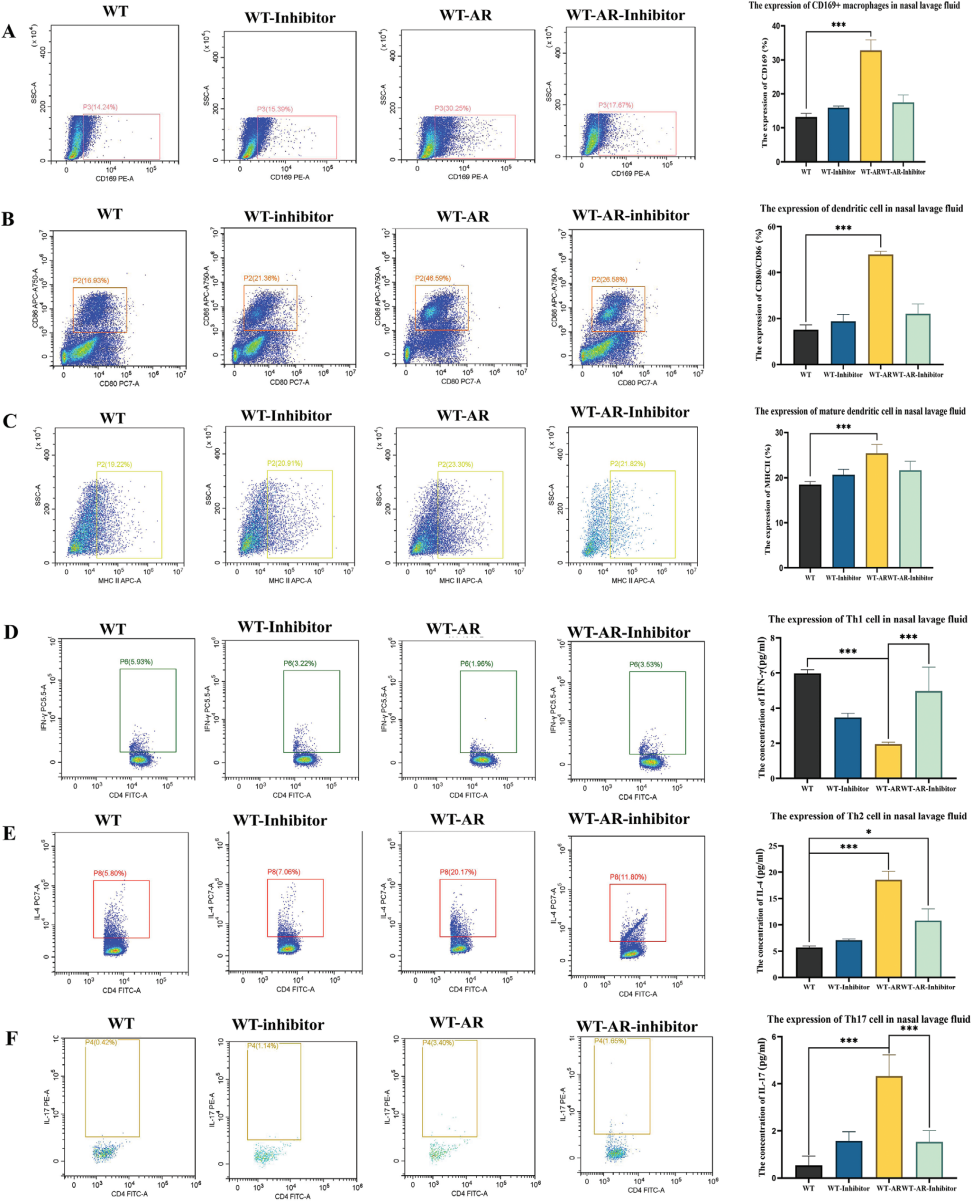
Effects of SLC38A2 inhibitors on immune cells and inflammatory factors in mouse nasal lavage fluid. A) The expression of CD169+ macrophages in mouse nasal lavage fluid was detected by flow cytometry. B) Flow cytometry was used to detect the expression of dendritic cells in the nasal lavage fluid of mice. C) The expression of mature dendritic cells in mouse nasal lavage fluid was detected by flow cytometry. D) The expression of Th1 cells in mouse nasal lavage fluid was detected by flow cytometry. E) The expression of Th2 cells in mouse nasal lavage fluid was detected by flow cytometry. F) The expression of Th17 cells in mouse nasal lavage fluid was detected by flow cytometry. The bar graphs show the quantification of the results, with each value representing the mean ± SEM of three independent experiments. Statistical significance is shown using the unpaired two‐tailed Student's *t*‐test; *p < 0.05; ***p < 0.001.

The AR mouse model was reconstructed using wild‐type C57 female mice. After the mice were killed through nasal inhalation of a SLC38A2 inhibitor, the expression of CD169+ macrophages, dendritic cells, mature dendritic cells, and Th cells in the nasal lavage fluid of mice exposed to SLC38A2 inhibitor were detected via flow cytometry. CD169+ macrophages, dendritic cells, mature dendritic cells, Th2, and Th17 cells decreased, and the proportion of Th1 cells was elevated (**Figure** **10**).

## 3 Discussion

Studies have shown that allergic disorders, including allergic rhinitis, hinge upon an imbalance in the immune response between Th cells.^[^ ^32^ ^]^ Th cells regulate one another through secreted cytokines to maintain the balance of immune function in the body. Moreover, regulatory T cells (Tregs) are crucial for maintaining the immune tolerance of the mucosal barrier.^[^ ^33^ ^]^ Dendritic cells, the most common antigen‐presenting cells (APC), can absorb and process antigens, activate initial T lymphocytes, stimulate antigen‐specific immune responses, and regulate immune tolerance.^[^ ^34^ ^]^ Under typical physiological conditions, dendritic cells can maintain the relative equilibrium of Th2 cell function and resist invasions of extracellular pathogens by participating in humoral immunity. When DC regulation is uncontrolled, Th2 cells overreact and induce allergic diseases linked to Th2 cell immune disorders, including allergic rhinitis, conjunctivitis, drug allergy, atopic dermatitis, allergic asthma, and severe allergic diseases.^[^ ^35^ ^]^

CD169, also known as Sialoadhesin (Sialoadhesin, Sn), belongs to the sialic acid‐binding immunoglobulin type lectins (Siglecs) family. It is mainly involved in antigen capture and activation of adaptive immune function,^[^ ^36^ ^]^ expressed on the surface of specific macrophages. CD169 was first discovered in the central hematopoietic island of the mouse bone marrow by Professor Kroc's team in 1986. These macrophages, which express receptors for goat red blood cells without mediating phagocytosis, were subsequently named Siglec‐1, SN, or CD169. Significant changes in the numbers of CD169+ macrophages can be detected in human tissues, lymph nodes, and peripheral blood, particularly in neoplastic and autoimmune diseases.^[^ ^37^ ^]^ Surface markers of CD169+ macrophages in lymph nodes include CD68, MHC type II, CD11c, CD11b, and F4/80. CD169+ macrophages can directly interact with dendritic cells, B cells, and T cells through CD169, participating in immune regulation, antigen presentation, and exerting antiviral functions, thus garnering increasing attention.^[^ ^38^ ^]^ The successful development of CD169 gene knockout mice (DTR mice), has brought the role of CD169+ macrophages in immune regulation and their application in various disease models to the forefront. Recent research has revealed that CD169+ macrophages play a significant role in inflammatory responses, primarily driven by the TNF‐α produced by CD169 macrophages. For example, CD169+ macrophages in the colon can secrete CCL8, attracting monocytes that promote the occurrence and development of enteritis in a model of enteritis induced by dextran sodium sulfate (DSS). Deletion of CD169+ macrophages significantly alleviates enteritis symptoms. In the spleen, CD169+ macrophages capture blood‐derived apoptotic cells through CCL22‐CCR4, secrete CCL22, increase the number of Tregs, and induce immune tolerance.^[^ ^39^ ^]^ In animal models with blood‐brain barrier injury, CD169+ macrophages in the nervous system are significantly increased. Moreover, the inflammatory response of the nervous system is significantly reduced in the absence of CD169+ macrophages.

Metabolomics can directly reflect the state and substance changes within an organism's body, and it finds widespread application in clinical diagnosis, drug development, and microbiology research.^[^ ^40^, ^41^ ^]^ Metabolomics can be categorized into non‐target and target metabolomics using different test methods.^[^ ^42^ ^]^ Non‐target metabolomics aims to detect all endogenous small molecule metabolites to uncover the metabolic mechanisms underlying their changes,^[^ ^43^, ^44^ ^]^ while targeted analysis is suitable for analyzing specific metabolites.^[^ ^45^ ^]^ The application of metabolomics in the ear, nose, and throat (ENT) primarily focuses on the early diagnosis of laryngeal cancer, nasopharyngeal cancer, and other tumors, with limited research on targeted drugs.^[^ ^46^ ^]^ In this study, metabolomics technology was applied to investigate the mechanism of CD169+ macrophage generation and development in allergic rhinitis, offering new insights into treating this condition.

Both in vivo and in vitro experiments have demonstrated that CD169+ macrophages are essential in inducing the differentiation and migration of dendritic cells. To optimize knockout efficiency, a modeling pre‐experiment was conducted (Figures S2–S4, Supporting Information). The knockout of CD169+ macrophages before sensitization was observed to have a higher impact than knocking out after sensitization. After DTR mice were injected with diphtheria toxin, CD169+ macrophages were detected every other month (Figure S1, Supporting Information), revealing that CD169 macrophages essentially recovered within about 3 months. The recovery was faster in the spleen and nasal lavage fluid, with lymph nodes showing the slowest recovery. This study employed pathological sections of the inferior turbinate and nasal mucosa from AR patients and nasal mucosa sections from non‐AR patients for immunofluorescence staining. The results indicated the presence of CD11b+CD169+ macrophages and CD11b+CD301b+PDL2+ DCs (i.e., cDC2) aggregated in the lamina propria and perilymphatic ectopic lymphatic follicular region of the nasal mucosa in AR patients. Subsequently, CD169‐DTR mice were used to create an AR mouse model. The experiment comprised four groups: ordinary C57 mice in the WT group, normal C57 mice with an AR model in the WT‐AR group, transgenic mice treated with diphtheria toxin in the DTT group, and transgenic mice treated with diphtheria toxin and induced AR model in the DTT‐AR group. The study involved various aspects, including behavioral observations where the number of nose scratching and sneezing of mice within 10 min was recorded (Tables 1 and 2). The findings showed that the WT‐AR group exhibited significantly higher nose scratching and sneezing numbers than the WT group, confirming the effectiveness of the modeling. Notably, allergic behavior significantly decreased when CD169+ macrophages were knocked out, indicating that CD169+ macrophages contributed to allergic reactions. However, in non‐AR mice, the knockout of CD169 macrophages had no significant impact on allergic behavior compared to the WT group, suggesting that the knockout of CD169 macrophages did not affect non‐allergic behavior. Following the euthanasia of the mice, nasal irrigation fluid, peripheral blood, lymph nodes, spleen, and nasal mucosa were collected for flow cytometry of immune cell populations, cytokine ELISA, and H&E staining. As shown in Figure 3, these analyses confirmed the reduction in the expression of CD169+ macrophages in both the DTT and the DTT‐AR groups, with lymph nodes showing the most noticeable reduction. Eosinophil counts in the nasal mucosa of AR mice significantly decreased after CD169 macrophages were knocked out (the DTT‐AR group was lower than the WT‐AR group), while no changes were observed in non‐AR mice. CD169+ macrophages in nasal lavage fluid and spleen increased slightly in the DTT‐AR group, suggesting an increased expression of CD169+ macrophages in AR mice. CD169+ macrophage knockout resulted in a reduced proportion of dendritic cells, Th2, and Th17 cells, and the maturation of dendritic cells in AR mice, while the proportion of Treg cells increased. Changes in Th1 cells were not significant, indicating the potential differentiation of Th0 cells into other Th cell types. The ELISA assay of nasal lavage solution and peripheral blood serum indicated elevated expression of IL‐4, IL‐17, IL‐23, and IFN‐ γ in both nasal lavage solution and serum following sensitization. After CD169+ macrophages were knocked out, these cytokines exhibited varying degrees of reduction. Conversely, TGF‐ β suggested the potential for other macrophages to have compensatory effects.

Additionally, the phenotype of CD169+ macrophages was further identified in animal experiments. After sorting CD169 macrophages through flow cytometry and culturing them in vitro for 48 h, as shown in Figure 7, these cells were subjected to lipopolysaccharide (LPS) stimulation. The subsequent flow cytometry analysis revealed an increased proportion of CD80+ cells. However, there were no significant changes in the F4/80 rates. Dendritic cells can express co‐stimulatory molecules that facilitate the transformation of initial CD4+ T cells into Th cells. Activated CD4+ T cells differentiate into various effector CD4 T cells, mediating the immune response through the secretion of different cytokines. In addition to Th1 and Th2 cells, researchers have identified Th17, Th9, Th22 cells, follicular T‐helper cells, and Treg cells capable of suppressing the immune response. When CD169+ macrophages were knocked out, Th2 cells decreased, and the proportion of Th1 cells decreased slightly in nasal lavage fluid and lymph nodes, with no significant changes in the spleen. This may be due to the differentiation of Th0 cells into other Th cell types.

To investigate the regulatory role of CD169+ macrophages on dendritic cells, metabolomic tests were conducted on nasal lavage fluid from DTR transgenic mice. The research team employed high‐resolution, non‐targeted metabolomics technology and ultra‐high‐performance liquid chromatography‐time mass spectrometry (UHPLC‐Q‐TOF MS) to detect metabolites in the samples. Identifying metabolites in biological samples involved matching information such as metabolite retention time, molecular mass (with a molecular mass error within <10 ppm), secondary fragmentation spectra, collision energy, and other information in the local database. These identification results were rigorously reviewed and manually confirmed. As depicted in Figure 6, the hotellings T2 diagram of the positive ion model population sample suggests minimal variation due to instrument errors throughout the study. PCA examination demonstrated that the quality control samples formed tight clusters in positive and negative ion modes, indicating excellent repeatability and reliable experimental data. In this project, a total of 1016 metabolites were identified across both positive and negative ion modes, and they were subjected to classified statistics. The results are presented in Figure 6, emphasizing the significant changes in the alanine pathway.

To identify metabolites capable of predicting the efficacy of subcutaneous immunotherapy for seasonal allergic rhinitis through serum metabolomics, Yu et al.^[^ ^47^ ^]^ recorded 43 cases of Artemisia pollen AR patients undergoing 1 year of subcutaneous immunotherapy, dividing patients into an ineffective group (n = 10) and an influential group (n = 33) based on the treatment outcomes. A metabolomic analysis was carried out using liquid chromatography‐mass spectrometry combined with gas chromatography‐mass spectrometry to identify differential compounds and related metabolic pathways. A total of 129 differential metabolites were identified, spanning four metabolic pathways: taurine and hypotaurine metabolism, pentose and glucuronic acid interconversion, pentose phosphate metabolism, and alanine, aspartic acid, and glutamate metabolism. These findings indicated that the alanine pathway holds potential as a predictive biomarker for effective subcutaneous immunotherapy, aligning with our research group's metabolomics findings.

Research has shown that alanine metabolism is associated with oxidative stress.^[^ ^48^, ^49^ ^]^ Nrf2 is a critical transcriptional regulator of anti‐oxidative stress mechanisms.^[^ ^50^ ^]^ In normal conditions, the Neh2 domain in Nrf2 binds to the DGR domain in Keap1 in the cytoplasm, leading to its degradation through Cul3/Rbx1 E3 ubiquitination, thus maintaining its stability. Upon stimulation by endogenous and exogenous oxidants, such as ROS and ozone, Nrf2 and Keap1 disassemble Nrf2 in the cell nucleus. There, it forms a heterodimer with Nej1 by binding with Maf protein and activates downstream antioxidants like HO‐1, SOD, and CAT, among others, to combat oxidative stress.^[^ ^50^, ^51^, ^52^, ^53^ ^]^ Studies have suggested that airway inflammation and hyperreactivity in mouse models of allergic asthma may be related to Nrf2 expression disorders.^[^ ^54^ ^]^ Other research has found higher Nrf2 protein and Nrf2 mRNA expression in children with severe asthma than those with mild asthma.^[^ ^55^ ^]^ MDA, a product of membrane lipid peroxidation, reflects the body's internal antioxidant capacity and the level of oxidative stress.^[^ ^56^ ^]^ Zhang Yan et al. found that high levels of MDA can induce pathological changes in the lungs of asthmatic mice.^[^ ^57^ ^]^ CAT and GSH are essential antioxidant enzymes that remove oxidative substances and mitigate oxidative stress damage. Both are downstream antioxidant proteases of the Keap1/Nrf2/HO‐1 signaling pathway, and their expression is regulated by this pathway.^[^ ^58^ ^]^ The Keap1/Nrf2/HO‐1 signaling pathway is a protective mechanism for various organs under different stress conditions. It protects against oxidative stress damage in the body's multiple organs and represents a focal point in studying the pathogenesis of oxidative stress‐related diseases. This pathway contributes to anti‐inflammation, anti‐oxidation, regulation of Ca^2+^ inflow, cell death modulation, mitochondrial damage mitigation, iron‐mediated cell death, pyroptosis regulation, and autophagy regulation.^[^ ^59^ ^]^

To delve further into the mechanism of CD169 macrophages in allergic rhinitis, flow cytometry was employed to extract CD169 macrophages from mouse spleen (Figure 7). Western blot was utilized to investigate the protein content expression of the Keap1/Nrf2/HO‐1 pathway in these cells (Figure 8). Additionally, qPCR was used to assess changes in mRNA levels in this pathway (Figure 8A–C). ELISA technology was employed to measure alterations in oxidative stress products, specifically MDA and HNE, in the cell culture medium (Figure 8F,G). These analyses indicated that the expression of Nrf2 and HO‐1 was elevated in CD169 macrophages treated with LPS, whereas the expression of Keap1 was reduced. Notably, this effect could be reversed after transfection with shKEAP1. These findings suggest that CD169 macrophages can up‐regulate alanine production and levels of ROS via the Keap1/Nrf2/HO‐1 axis, subsequently releasing these factors into the nasal mucosal microenvironment.

Existing literature has demonstrated that SLC38A2 plays a role in mediating the intracellular transport of alanine, thereby maintaining cellular stability. Cells lacking SLC38A2 cannot concentrate intracellular alanine and experience severe metabolic crises, which result in significantly impaired cell growth.^[^ ^60^, ^61^ ^]^ A co‐culture system for CD169 macrophages and dendritic cells was constructed (Figure 9A), and an alanine detection kit was employed to measure the changes in alanine content in the cell culture medium following co‐culture. The results revealed that the SLC38A2 inhibitor significantly reduced alanine uptake by DCs (Figure 9B). Additional transwell and flow cytometry experiments corroborated that the SLC38A2 inhibitor could suppress the migration and maturation of dendritic cells following co‐culture (Figure 9C–K) and in vivo experiments have also confirmed this problem as Figure 10 shown.

Although our study has confirmed that CD169+macrophages play an extremely important role in allergic rhinitis, there are still many problems to be solved in our study. For example, in our study, we used OVA to establish allergic rhinitis mouse model, but many studies have shown that using dust mites to establish allergic rhinitis may be more consistent with the natural environment sensitization process.^[^ ^2^, ^62^ ^]^ In the next study, we will try to use dust mites instead of OVA to establish allergic rhinitis model, so as to explore whether there are differences in the role of CD169+macrophages in different allergic rhinitis models, and whether there are new pathogenesis.

This study demonstrated that CD169+ macrophages can up‐regulate their internal alanine production and increase ROS levels through the Keap1/Nrf2/HO‐1 axis. These substances are then released into the nasal mucosal microenvironment, ultimately promoting alanine uptake by dendritic cells through SLC38A2. This regulation of dendritic cell migration and maturation contributes to the cascade of allergic rhinitis. The research findings expand our understanding of the role of CD169+ macrophages and provide a novel target for potential diagnosis and treatment in patients with AR. However, more in‐depth and comprehensive studies are still needed on the mechanism of CD169+ macrophages in allergic rhinitis. For example, in our next study, we will try to use dust mites to replace ovalbumin to build a model of allergic rhinitis, so as to explore whether there are differences in the role of CD169+ macrophages in different allergy models and whether there is a new pathogenesis.

## 4 Experimental Section

### Cell Lines and Animals

In this study, DCs from human dendritic cell lines were acquired from the American Type Culture Collection (ATCC). The wild‐type C57BL/6 mice were purchased from Jinan Pengyue Laboratory Animal Co., LTD. CD169‐DTR mice were maintained by Gene&Peacebiotech Co., Ltd., (Jiangsu, China)., and were raised in the Animal Center of Shandong Provincial Hospital. The animal center had aseptic animal feeding conditions. The Ethics Committee of Shandong Provincial Hospital Affiliated to Shandong First Medical University reviewed and approved the animal study (NO.2020‐354). The animal experiment was divided into four groups: ordinary C57 mice were WT group; allergic rhinitis model of ordinary C57 mice were WT‐AR group; transgenic mice treated with diphtheria toxin were DTT group and transgenic mice treated with diphtheria toxin and constructed allergic rhinitis model were DTT‐AR group.

### Case Selection

A total of six female patients with AR and six female patients with simple deviated nasal septum were chosen from Shandong Provincial Hospital between 2020 and 2022. All patients had no other underlying diseases and had not undergone nasal spray therapy within 6 months. The studies involving human participants were reviewed and approved by the Ethics Committee of Shandong Provincial Hospital Affiliated to Shandong First Medical University (NO.2020‐443). The patients/participants provided written informed consent to participate in this study. Pathological samples from the inferior turbinate of patients were acquired during the operation. The samples were processed immediately (within 30 min), and three thin sections were obtained from each sample, totaling 36 sections.

### Materials

DMEM and RPMI‐1640 cell media were purchased from HyClone (USA), and fetal bovine serum (FBS) was acquired from Gibico (Australia). Ammonium chloride solution was acquired from YuanMu Biological Technology Co., Ltd in Shanghai. Recombinant murine IL‐4 and granulocyte‐macrophage colony‐stimulating factor (GM‐CSF) were purchased from USA PeproTech. Lipopolysaccharide, aluminum hydroxide, Ovalbumin (OVA, grade V), and 4′‐6‐diamidino‐ 2‐phenylindole (DAPI) were purchased from USA Sigma. The Western blot kit was purchased from Solarbio Science & Technology Co., Ltd in Beijing. Anti‐mouse IFN‐γ of percp/cy5.5‐conjugated, PE‐conjugated anti‐mouse IL‐4, Alexa Fluor 647‐conjugated anti‐mouse IL‐17α, APC‐conjugated anti‐mouse CD11c, anti‐mouse CD80 of FITC‐conjugated, and PE‐conjugated anti‐mouse CD86 were obtained from BioLegend (USA). The Mouse ELISA Kit for IL‐4, TNF‐α, CCL17, and IgE were purchased from Multisciences in China. The mouse histamine ELISA Kit was obtained from International Inc. (Germany). All other chemicals were of reagent grade and used directly as they were received.

### Immunofluorescence Staining of Human and Mice Pathological Sections

Initially, the selected pathological sections were incubated at 60 °C for 2 h, and then cooled to room temperature. We placed the sections into xylene, anhydrous ethanol liquid, 95% ethanol solution, 85% ethanol solution, 75% ethanol solution, and PBS buffer solution sequentially. We placed the tissue sections in a repair box containing sodium citrate repair solution for antigen repair and washed them at room temperature using PBS buffer. A 5% BSA solution was supplemented onto the tissue surface to uniformly cover the tissue, and the tissue was placed in an incubator at 37 °C for 30 min. The sealing solution was gently removed, and the primary antibody was added to incubate at 4 °C overnight. After overnight incubation, the slides were washed in PBS buffer. The corresponding fluorescent secondary antibody was added to cover the tissue and incubated for 1 h in darkness. The slides were washed using a PBS solution. After the slices were slightly dried, DAPI dye solution was added into the water‐blocking ring and incubated at room temperature for 3–5 min in the dark. Each slice had 100 µL anti‐fluorescence quenching agent added into the water‐blocking ring, and the slice was dried and sealed using resin. Image acquisition was performed using a fluorescence microscope (blue light for DAPI, green light for CD169‐CY3).

### Flow Cytometry of Dendritic Cells in Nasal Lavage Fluid, Lymph Nodes, and Spleen of Mice

100 µL of the cell suspension, which had been crushed and filtered to adjust the cell concentration, was mixed with 5 µL of MHCI‐APC, 1 µL of CD86‐APC‐Cy7, and 2 µL of CD80‐PE‐Cy72 flow antibody for staining, and incubated on ice for 30 min in the dark. Two milliliters of 1 x Permeabilization Buffer was added to each tube before being centrifuged at room temperature at 350 x *g* for 5 min, and the supernatant was discarded. An aliquot of 500 µL of 1 x Flow Cytometry Staining Buffer was added to each tube. It is possible to use a 5% formaldehyde solution to detect the staining without light under elongated detection times.

### Flow Cytometry of CD169+ Macrophages in Nasal Lavage Fluid, Lymph Nodes, and Spleen of Mice

100 µL of cell suspension, which had been crushed and filtered to adjust the cell concentration, was mixed with 0.5 µL of F4/80, 2 µL of CD169‐PE, 1 µL of CD11b‐APC, 1 µL of CD206‐PE‐Cy7, and 1 µL of CD86‐APC‐Cy7 flow antibodies for surface staining, and shaken. The mixture was incubated on ice in the dark for 30 min. An aliquot of 2 mL of 1 × Permeabilization Buffer was added to each tube before centrifugation at room temperature 350 × *g* for 5 min, and the supernatant was carefully discarded. An aliquot of 500 µL of 1 × Flow Cytometry Staining Buffer was added to each tube. It is possible to use a 5% formaldehyde solution to detect the staining without light under elongated detection times.

### Flow Cytometry of Th Cells in Mouse Nasal Lavage Fluid, Lymph Nodes, and Spleen

DMEM medium supplemented with 10% fetal bovine serum was used to precipitate the cells, with a cell concentration of 1 × 10^7^ cells mL^−1^. A mixture of 250 µL of the cell suspension was added into the flow tube, and 1 µL of PMA (250×) and 1 µL of BFA (250×) were added. Samples containing only cell suspension lacking antibodies were employed as controls. After aspirating the mixed cells, they were incubated at 37 °C for 7 h and mixed every hour by shaking. A sample of 100 µL of the cell suspension was obtained from the sample tube and transferred into a new flow tube, alongside an appropriate amount of CD3‐FITC (2 µL) and CD4‐PE (1 µL). The cell suspension was mixed and incubated in the dark at room temperature for 15 min. A 100 µL aliquot of FIX & PERM Medium A reagent was added to each tube, and the suspension was mixed and incubated at room temperature in the dark for 15 min. An aliquot of 2 mL of 1 × Flow Cytometry Staining Buffer solution was added to each tube and pre‐cooled, centrifuged at 300 × *g* for 5 min, and the supernatant was discarded. Each tube was mixed with 100 µL of FIX & PERM Medium B and 5 µL of IL‐4‐PE‐Cy7, 5 µL of IFN‐γ‐PC5.5, and 1 µL of IL‐17‐PE. After aspirating and mixing the suspension, it was incubated at room temperature in the dark for 15 min. An addition of 2 mL of 1 × Flow Cytometry Staining Buffer reagent was made to each tube. Tubes were centrifuged at 300 × *g* for 5 min, and the supernatant was discarded carefully. An aliquot of 500 µL of 1 × Flow Cytometry Staining Buffer was added to each tube (5% formaldehyde solution could also be employed in the case of elongated for long detection time) of the staining cell suspension, and staining was detected in the dark.

### Treg Cell Flow Cytometry in Nasal Lavage Fluid, Lymph Nodes, and Spleen of Mice

100 µL of cell suspension was obtained, which had been crushed and filtered to adjust the cell concentration, and 0.5 µL of CD4‐FITC and 1 µL of CD25‐APC antibodies were included for surface staining. The mixed cell suspension was aspirated and incubated on ice in the dark for 30 min. A sample of 2 mL of red cell lysate was added to each tube, and the mixed cell suspension was aspirated before being incubated in the dark at room temperature for 15 min. The samples were centrifuged at room temperature at 300 to 400 × *g* for 5 min, and the supernatant was discarded. Two milliliters of 1 × Flow Cytometry Staining Buffer reagent was added to each tube. The cell suspension was mixed, centrifuged at room temperature at 300 × *g* for 5 min, and the supernatant was carefully discarded. Each tube was mixed with 1 mL of Fixation/Permeabilization buffer and mixed at room temperature in the dark for 1 hour. Without washing, 2 mL of 1 × Permeabilization Buffer was added to each tube, and the mixture was centrifuged at room temperature at 300 × *g* for 5 min, before carefully discarding the supernatant. The cells were precipitated using 100 µL of 1×Permeabilization Buffer reagent. Five microliters of FoxP3 antibody and the same mass number of the control were added. The mixed cell suspension was aspirated and incubated at room temperature for 30 min in the dark. Two milliliters of 1×Permeabilization Buffer reagent was added to each tube, which was then centrifuged at room temperature at 300 × *g* for 5 min, and the supernatant was carefully discarded (the lower liquid layer could be removed using a pipette tip). An aliquot of 500 µL of 1 × Flow Cytometry Staining Buffer reagent was added to each tube for staining detection.

### ELISa Detection of Cytokines in Nasal Lavage Fluid and Serum of Mice

Nasal lavage solution/serum was centrifuged at 300 × *g* at 4 °C for 10 min to remove the precipitate, and the supernatant was obtained for immediate testing. A 300 µL aliquot of 1 × wash buffer was incubated with the sample at room temperature for 30 s. After pouring and removing the washing liquid, the enzyme‐labeled plate was patted dry on absorbent paper until dry. An addition of 100 µL of twice diluted standard was made to each standard well, and 100 µL of the standard diluent was added to the blank well. Nasal lavage solution/serum was included in the sample well alongside 80 µL of 1 × detection buffer and 20 µL of the sample. A sample of 100 µL of cell culture supernatant was added to the sample well, and 50 µL of detection antibody diluted at 1:100 per well was added. The plate was sealed, ensuring the absence of bubbles, shaken on an oscillator at 300 rpm, and incubated at room temperature for 2 h. The original liquid was removed, and 300 µL of new liquid was added to each well to wash the plate, which was thoroughly cleaned six times, before being pat dry using absorbent paper. A sample of 100 µL of horseradish peroxidase streptavidin diluted at 1:100 per well was added. The plate was once again sealed using a clean sealing film. The plate was shaken on the oscillator at 300 rpm and incubated at room temperature for 1 hour. A sample of 100 µL of color‐developing substrate TMB was added to each well and incubated at room temperature for 30 min in the dark. Termination liquid was added in the amount of 100 µL to each well, and the color was observed to change from blue to yellow after being allowed to stand. If the color change was not noticeable, samples were mixed thoroughly. Within 30 min, the enzyme marker was examined using an absorbance of 450 nm.

### AR Model Mouse Modeling

Forty female C57BL/6 mice were randomly separated into the control group (WT) and the AR group (WT‐AR). Additionally, 40 female CD169‐DTR mice were randomly divided into two groups: the CD169+ macrophage knockout group (DTT) and the CD169+ macrophage knockout and sensitization group (DTT‐AR). The WT group was intraperitoneally injected with 400 µL of normal saline on day 1 and day 8, and intranasally administered 10 µL of normal saline every day for 4 weeks beginning on day 9. The WT‐AR group was intraperitoneally injected with 400 µL of ovalbumin aluminum hydroxide suspension (containing 20 µg ovalbumin) on day 1 and day 8, and each mouse was administered 10 µL ovalbumin solution via nasal drip daily for 4 weeks beginning on day 9. The DTT group was administered 10 µg of Diphtherin (DT) per kg of body weight on day 1 and day 21, 400 µL of normal saline was injected intraperitoneally on day 4 and day 12, and 10 µL of normal saline was administered daily for 4 weeks beginning on day 13. The DTT‐AR group was administered 10 µg of Diphtherin (DT) per kg of body weight on day 1 and day 21, intraperitoneally injected with 400 µL of ovalbumin aluminum hydroxide suspension (containing 20 µg of ovalbumin) on day 4 and day 12, and given 10 µL of each ovalbumin solution daily for 4 weeks beginning on day 13. On the 41^st^ day, blood was obtained from the eyeballs of all mice. Posthumously, nasal lavage solution, neck, mesenteric lymph nodes, and spleen were obtained for flow cytometry, H&E staining, ELISA detection, and other follow‐up experiments.

### H&E Staining of Mouse Lymph Nodes, Spleen, and Nasal Mucosa

After incubating for 2 h at 60 °C, the sections were placed at room temperature. The selected sections were placed into xylene I solution and allowed to stand for 15 min to infiltrate fully, followed by xylene II solution for 15 min, anhydrous ethanol I for 5 min, anhydrous ethanol II for 5 min, 95% ethanol for 5 min, 85% ethanol for 5 min, 75% ethanol for 5 min, and PBS buffer for 5 min. The sections were placed into the hematoxylin solution for staining for ≈5 min (while observing discoloration), and the staining was terminated using tap water. After rinsing with running tap water, the sections were placed in 0.6% ammonia water for pH balancing, and rinsed with tap water again. Slices were placed into an eosin dye solution and stained for 1 to 3 min. The sections were then incubated in 95% ethanol I for 5 min, 95% ethanol II for 5 min, anhydrous ethanol I for 5 min, anhydrous ethanol II for 15 min, xylene solution I for 15 min, xylene solution II for 15 min to dehydrate and clear the slides before being sealed with neutral gum.

### Dendritic Cells and CD169+ Macrophage co‐Culturing

Flow‐sorted CD169+ macrophages and dendritic cells were co‐cultured using the Transwell co‐culture system. Dendritic cells (DC) were seeded into the upper chamber, and flow‐sorted CD169+ macrophages were seeded into the lower chamber. After 12 h of co‐culture, a SLC38A2 inhibitor was added to the lower chamber fluid, and a follow‐up experiment was conducted after 48 h of culture.

### Cell Scratch Experiment

In advance, a ruler and marker were placed on an ultra‐clean table for at least 30 min under ultraviolet irradiation to sterilize. A horizontal line was drawn using a marker on the opposite side of the 6‐well plate, crossing the well every 1 cm, with a total of 5 lines drawn. The cells were selected, mixed with 2 mL of DMEM culture solution supplemented with 10% FBS, inoculated on six‐well plates with a cell density of 5 × 10^5^ cells well^−1^, and placed in an incubator at 37 °C overnight. The next day, a 200 µL pipette was used to scratch along the previous day's horizontal line. The suspended cells were washed three times using 5 mL of PBS buffer for removal, observed under the microscope, and photographed before being recorded and retained immediately. The culture medium and CD169+ macrophages and/or SLC38A2 inhibitor were supplemented, and the control group was cultured in an incubator for 24 or 48 h in the absence of any drugs. After culturing for this 24‐ or 48‐h period, the culture medium was removed, washed with PBS buffer, and photographed under a microscope.

### Transwell Experiment

The cells were digested and resuspended in medium. Following cell counting, the cells were adjusted to 2.5 × 10^5^ cells mL^−1^. Medium supplemented with 10% FBS was added to the lower part of the Transwell compartment at a volume of 500 µL, while 300 µL of medium containing serum‐free cells was added to the upper portion. The plate was incubated at 37 °C and washed with PBS buffer 1 day later. The plate was fixed with 4% paraformaldehyde solution for 30 min and washed with PBS three times. A 1 mL aliquot of 0.1% crystal violet solution was added, allowed to stand for 10 min, rinsed under running water, and the upper layer of non‐migrated cells was gently wiped off. The collected images were assessed under a fluorescence microscope. Three high‐magnification fields (200×) were randomly counted for each specimen, and the mean value was calculated.

### Metabolomics Analysis of Mouse Nasal Lavage Fluid

After the nasal irrigation solution was thawed on ice, samples were obtained and added to the pre‐cooled dissolved solution (methanol/acetonitrile/water = 2:2:1) and vortexed. Ultrasound exposure was conducted at a low temperature for 30 min, and the mixture was allowed to stand at −20 °C for 10 min. The mixture was centrifuged at 14 000 × *g* at 4 °C for 20 min, the supernatant was removed, and the pellet was vacuum‐dried. A 100 µL aliquot of acetonitrile aqueous solution (acetonitrile:water = 1:1, v/v) was added for resolution, and mass spectrometry analysis was conducted. After vortex mixing, the suspension was centrifuged at 14 000 × *g* at 4 °C for 15 min, before carefully absorbing the supernatant, and performing sample analysis. The separation was conducted using a HILIC column, with a column temperature of 25 °C. The flow rate was set at 0.5 mL min^−1^, and a sample size of 2 µL. The mobile phase comprised A:water+25 mM ammonium acetate+25 mM ammonia water, and B: acetonitrile. The gradient elution procedure was as follows: 0 to 0.5 min, 95% B; 0.5 to 7 min, 65% B; 7 to 8 min, 40%B; 8 to 9 min, 40%B; 9 to 9.1 min, 95%B; 9.1 to 12 min, 95%B. The samples were placed in a 4 °C automatic injector throughout the analysis. A random method was adopted to conduct continuous analysis of the samples in order, and quality control samples were inserted into the sample queue to ensure the stability and reliability of the obtained data.

### Statistical Analysis

Each experiment was conducted independently in at least triplicate. Statistical analysis was carried out using IBM SPSS Statistics V23.0 software. Data are presented as mean ± standard error of mean of three independent experiment. For analysis of the statistical differences between the two groups, a unpaired two‐tailed student's *t*‐test was used, and a one‐way analysis of variance (ANOVA) was employed for analysis between two or more groups. *p* < 0.05 was considered to be statistically significant and *p < 0.05; **p < 0.01; ***p < 0.001.

## Conflict of Interest

The authors declare no conflict of interest.

## Author Contributions

W.Q., C.L., and L.S. contributed equally to this work. XM and ZMQ provided direction and guidance throughout the preparation of the manuscript. GLL made a significant contribution to the revision of the article. QWW, SL, and LCC performed the literature search and wrote the original manuscript. ZXC, WXH, and GXC provided constructive suggestions and significantly revised the manuscript. ZFY, GN, and BX conducted a detailed analysis of the data in the article. CX, LCZL, and HHY collected clinical samples. YFY and DYH conducted experimental operations. All authors read and approved the final manuscript.

## Supporting information



Supporting Information

## Data Availability

The data that support the findings of this study are available from the corresponding author upon reasonable request.
