# Supplementary material for: CD169+ Macrophages Mediate the Immune Response of Allergic Rhinitis Through the Keap1/Nrf2/HO‐1 Axis
Source: Adv Sci (Weinh). 2024 Oct 22;11(45):2309331. doi: 10.1002/advs.202309331 (PMC11615775; doi:10.1002/advs.202309331)
Supplement: Supplementary file 1 — Supporting Information [file ADVS-11-2309331-s001.pdf]

ADVANCED SCIENCE
Open Access

# Supporting Information

for *Adv. Sci.*, DOI 10.1002/advs.202309331

CD169+ Macrophages Mediate the Immune Response of Allergic Rhinitis Through the Keap1/Nrf2/HO-1 Axis

*Wenwen Qi, Chengcheng Liu, Lei Shi, Hui Li, Xiaozhi Hou, Hongjie Du, Luqiu Chen, Xiaochen Gao, Xue Cao, Na Guo, Yuhan Dong, Chengzhilin Li, Fanyu Yuan, Zhenxiao Teng, Houyang Hu, Fangyuan Zhu, Xuanchen Zhou, Lulu Guo*, Miaoqing Zhao* and Ming Xia**

# Raw Data

## FIG1A

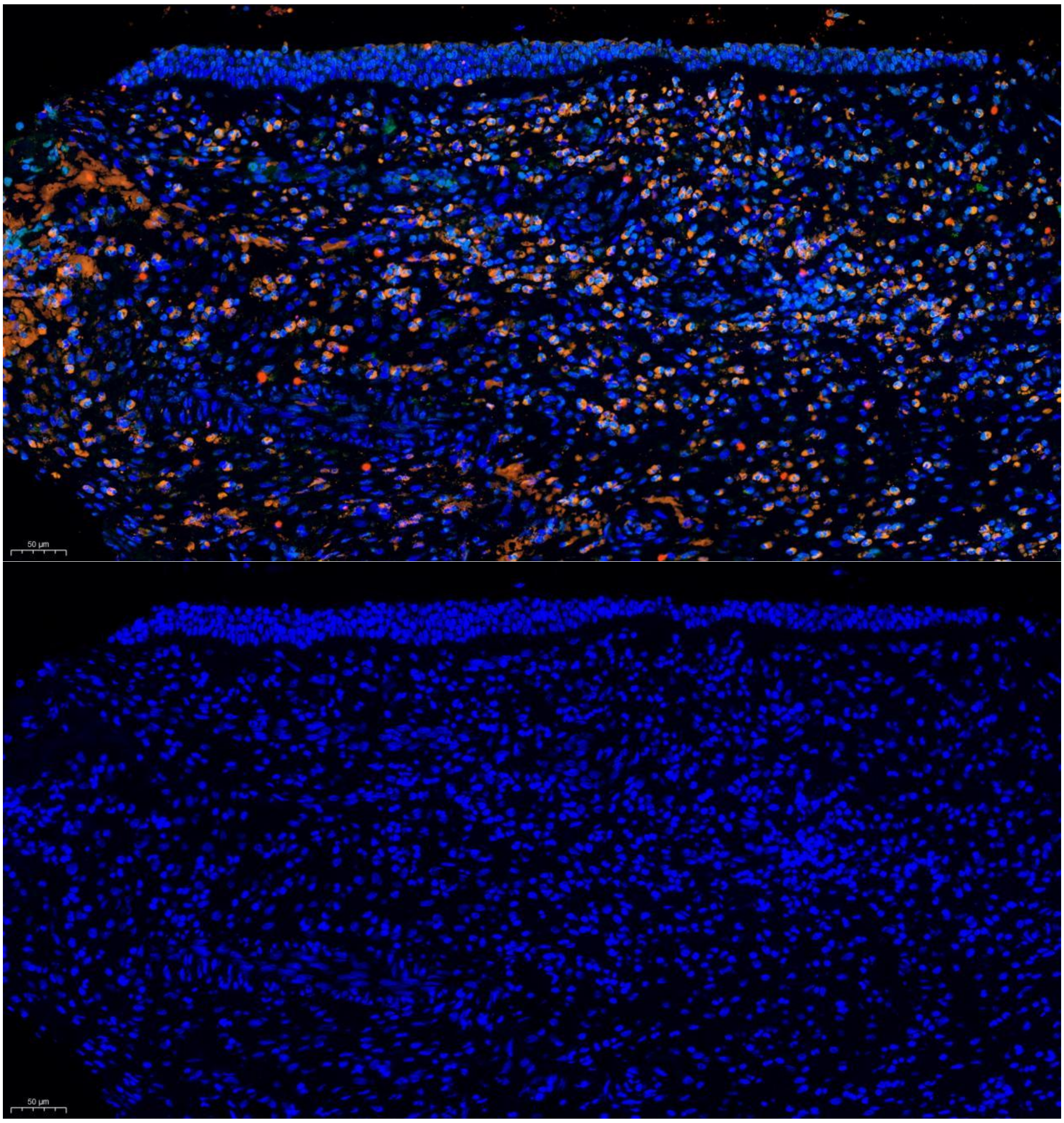

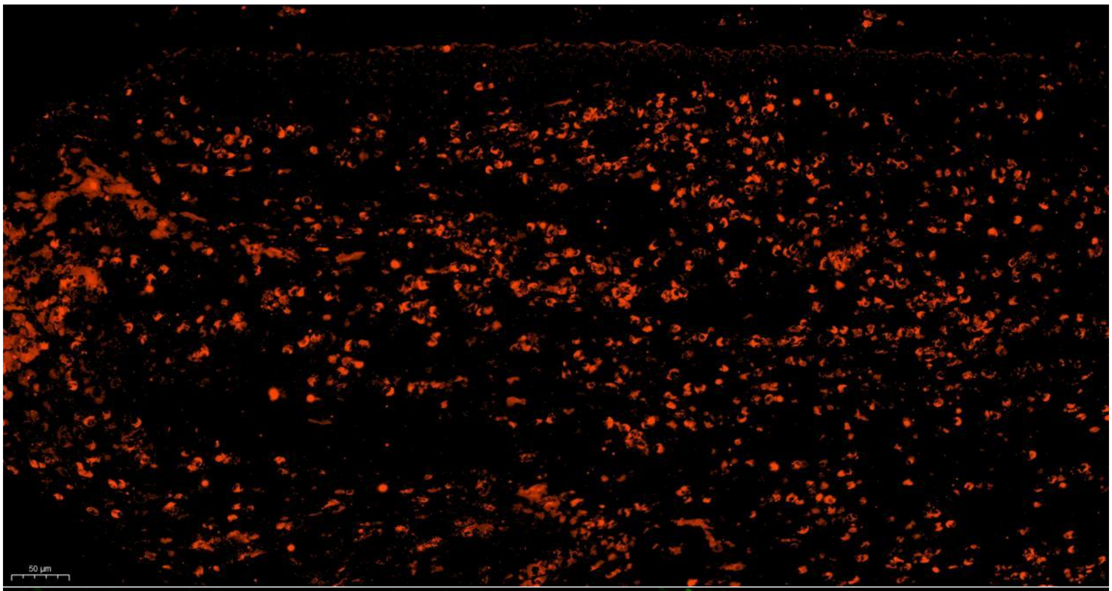
50 µm

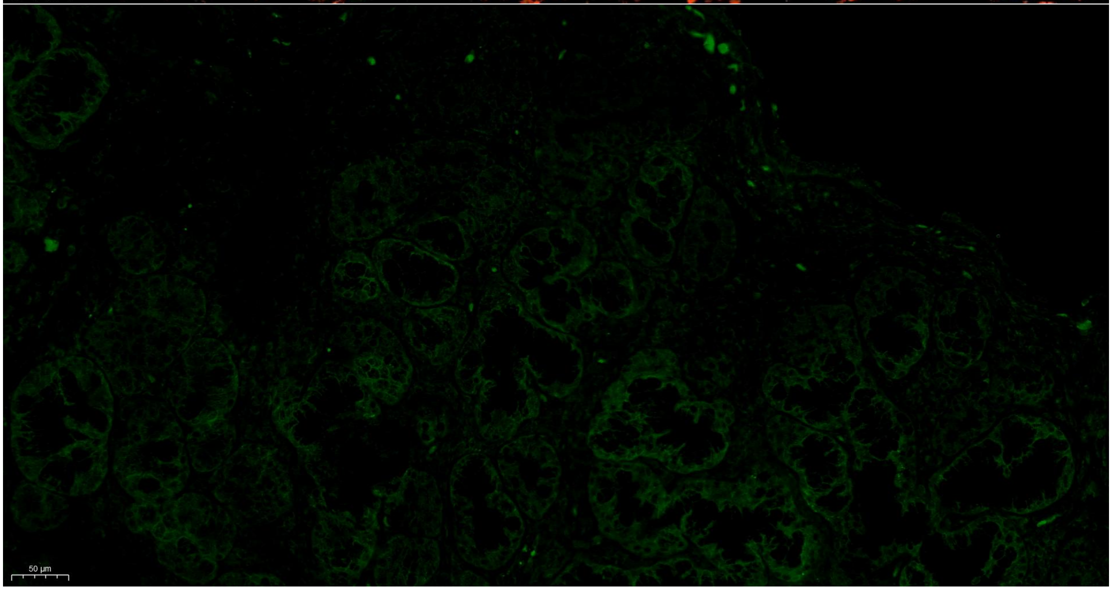
50 µm

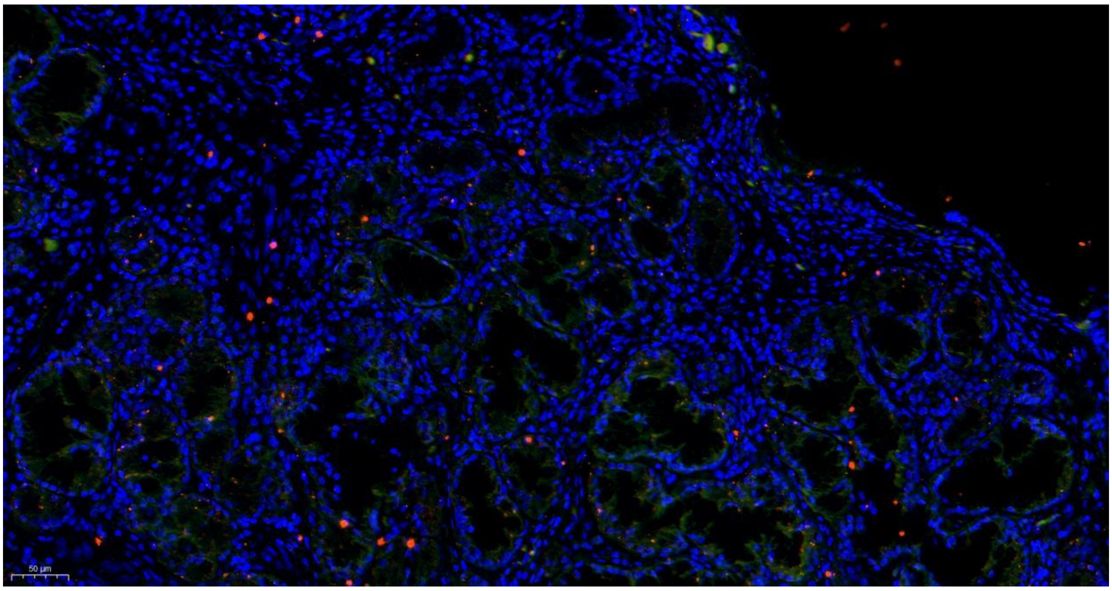
50 µm

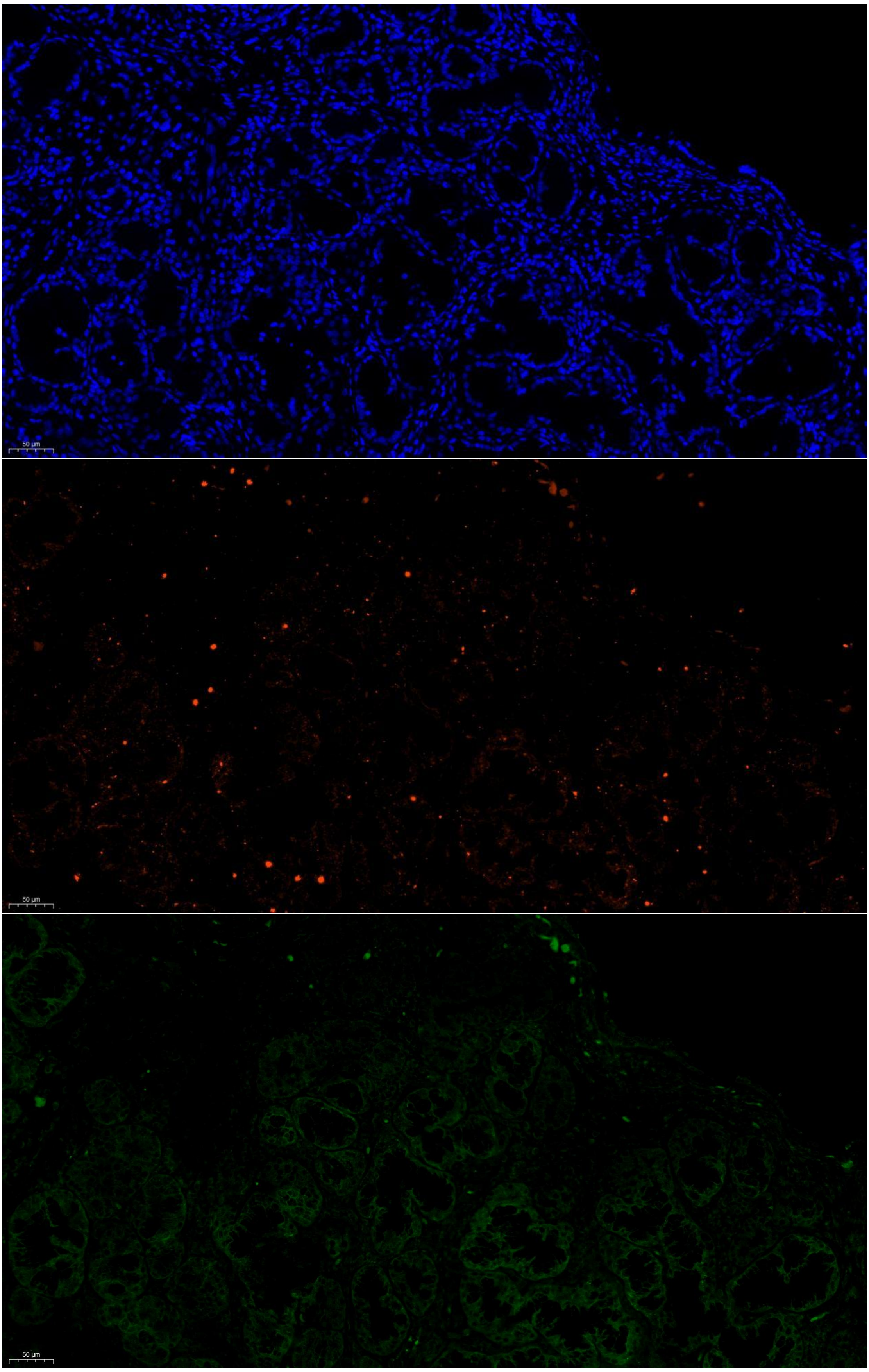
50 µm
50 µm
50 µm

## FIG1D

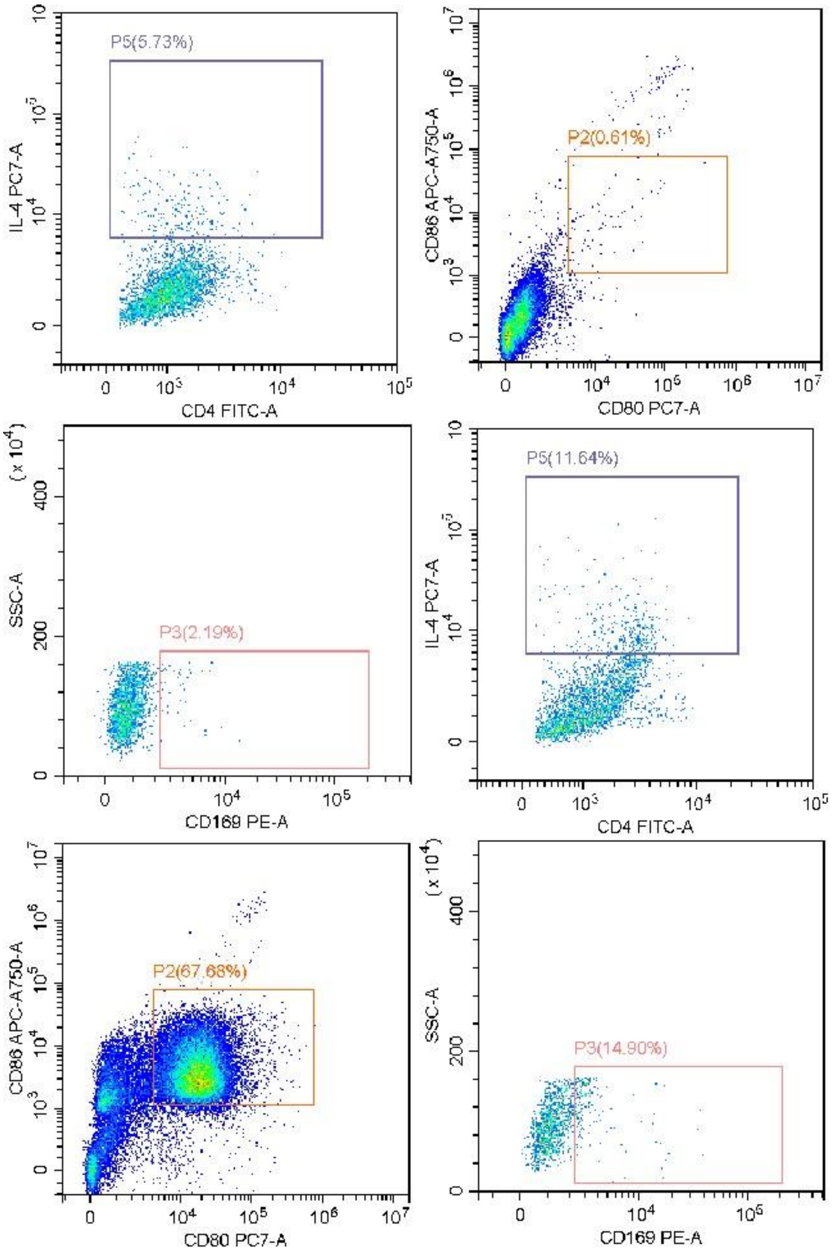
P5(5.73%)
IL-4 PC7-A
CD4 FITC-A
P2(0.61%)
CD86 APC-A750-A
CD80 PC7-A
P3(2.19%)
SSC-A
(x 10^4)
CD169 PE-A
P5(11.64%)
IL-4 PC7-A
CD4 FITC-A
P2(67.68%)
CD86 APC-A750-A
CD80 PC7-A
P3(14.90%)
SSC-A
(x 10^4)
CD169 PE-A

## FIG2C

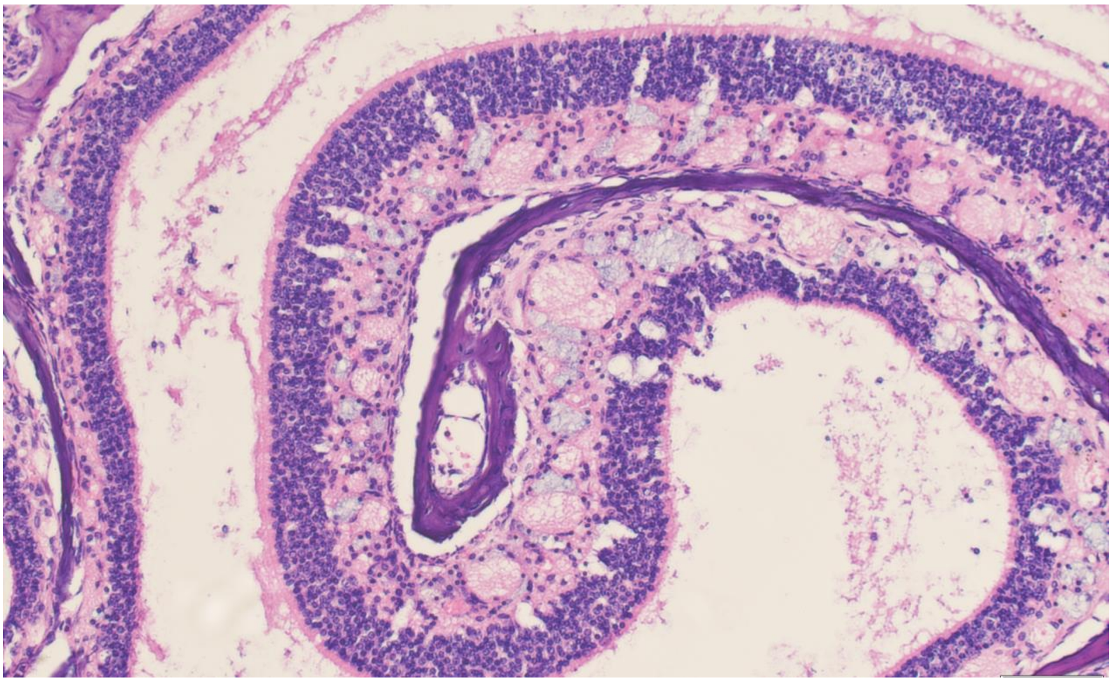

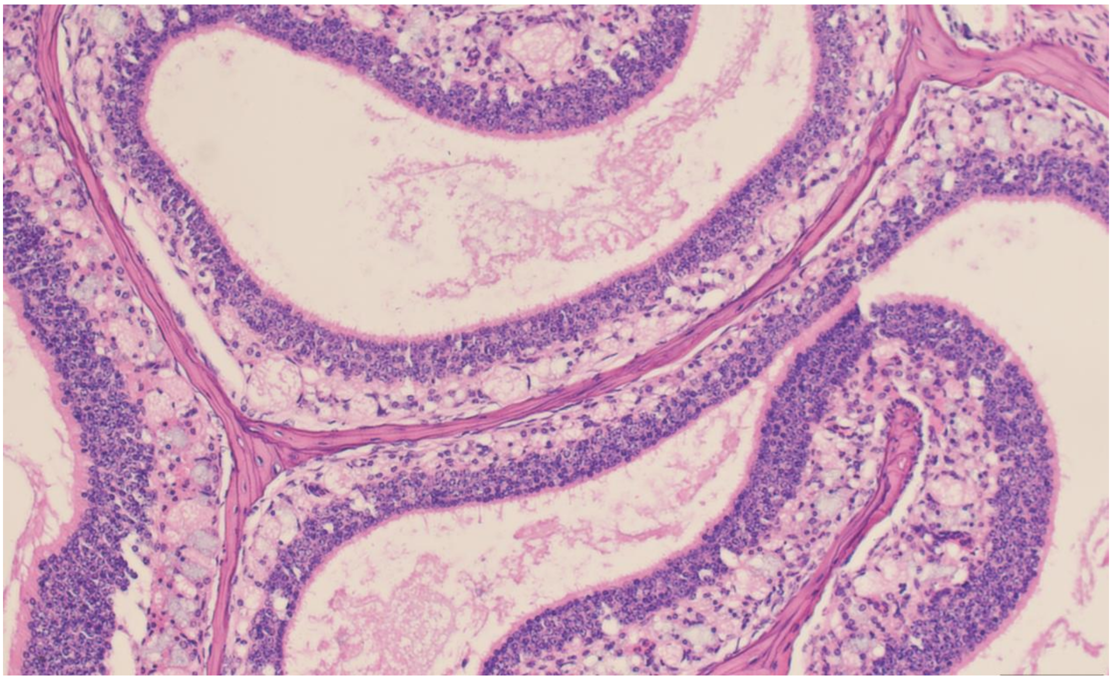

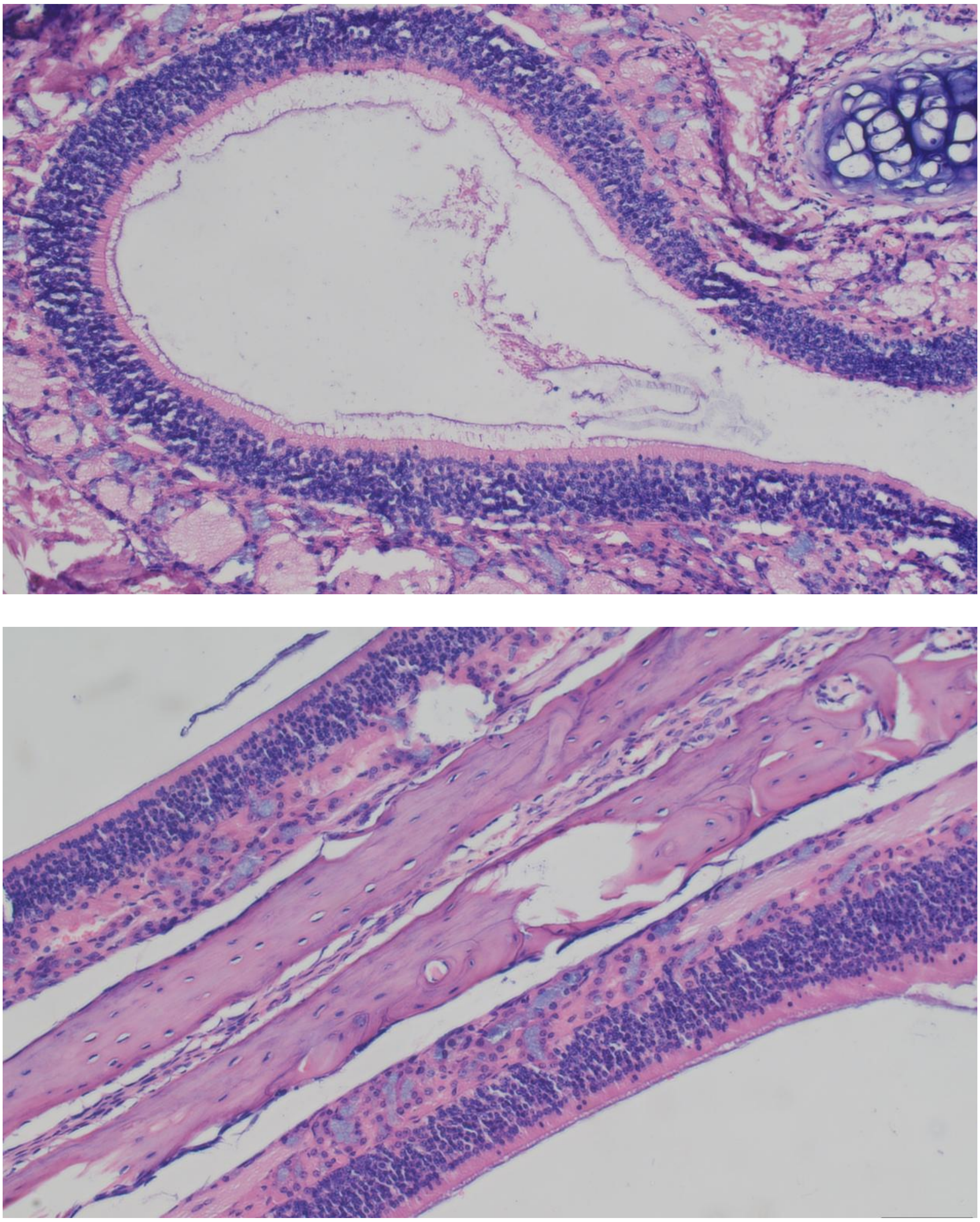

**FIG3A**

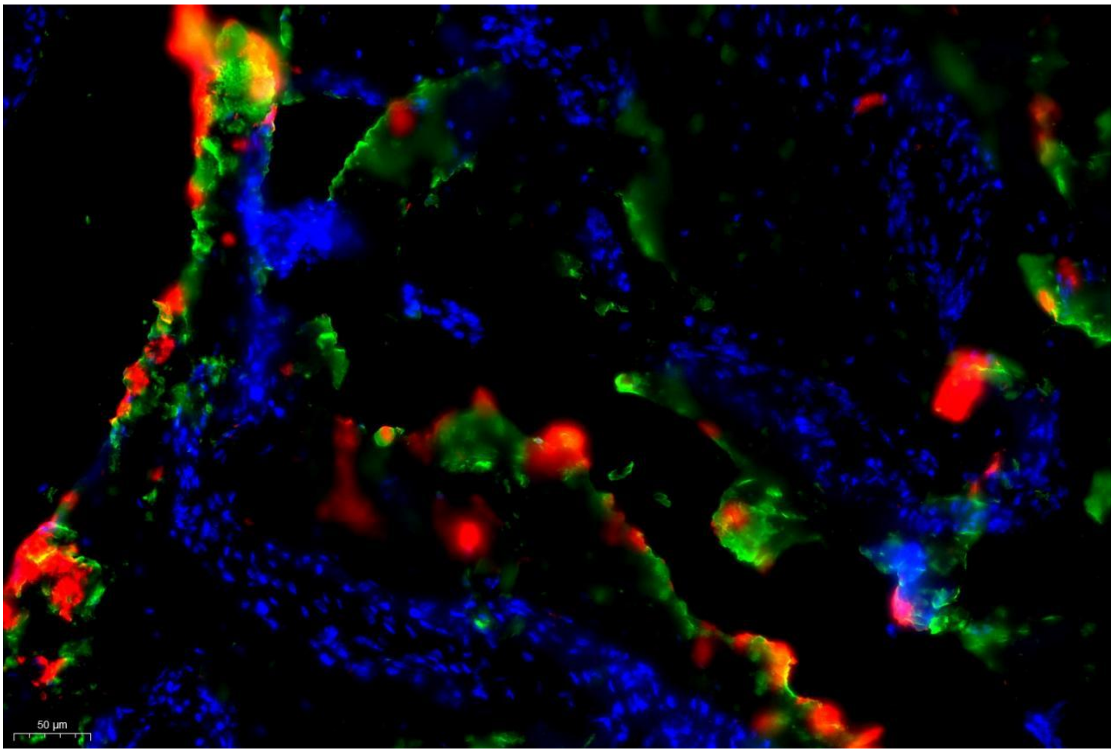
50 µm

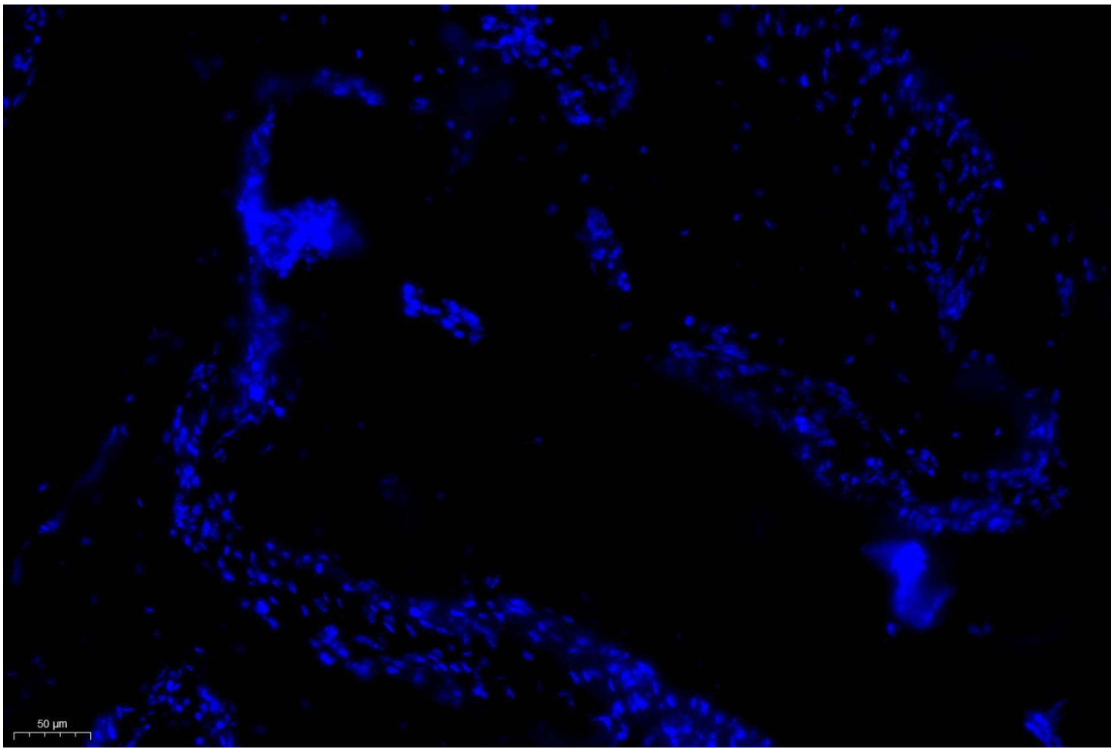
50 µm

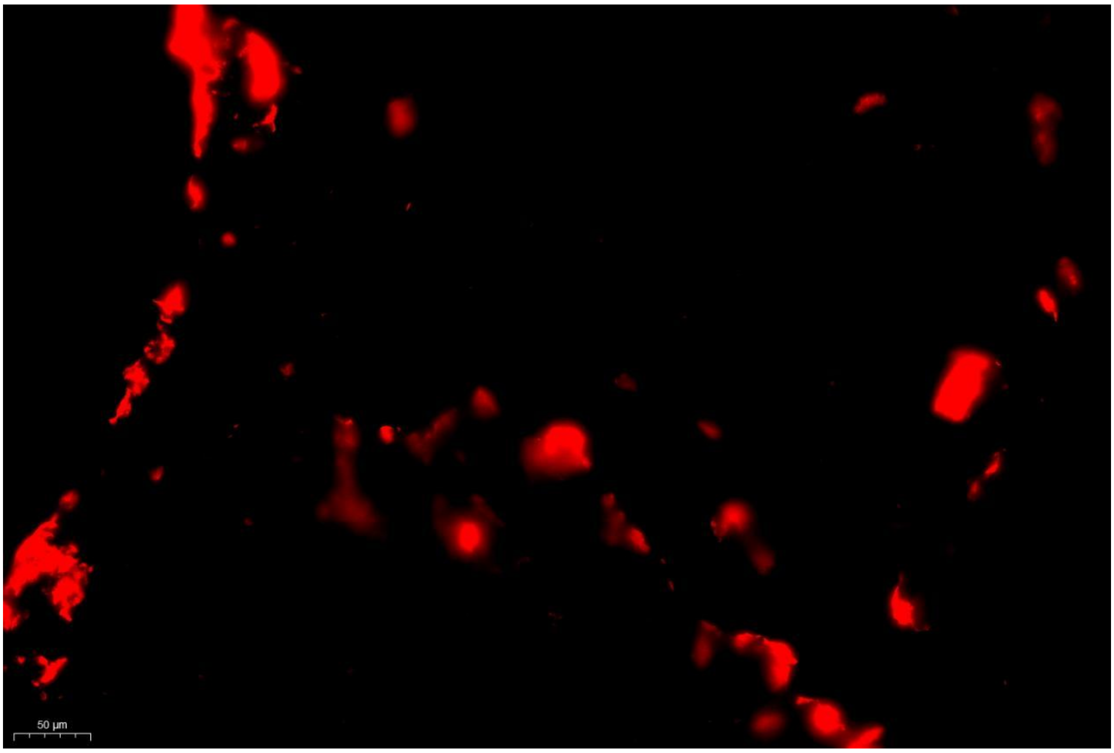
50 µm

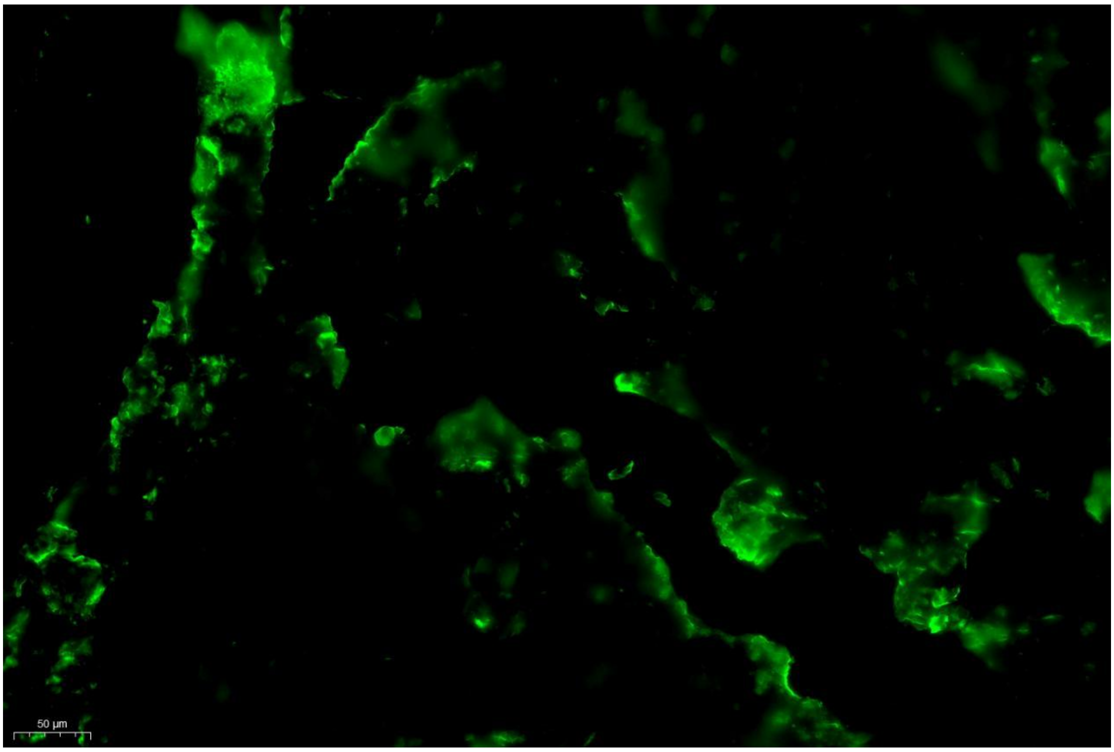
50 µm

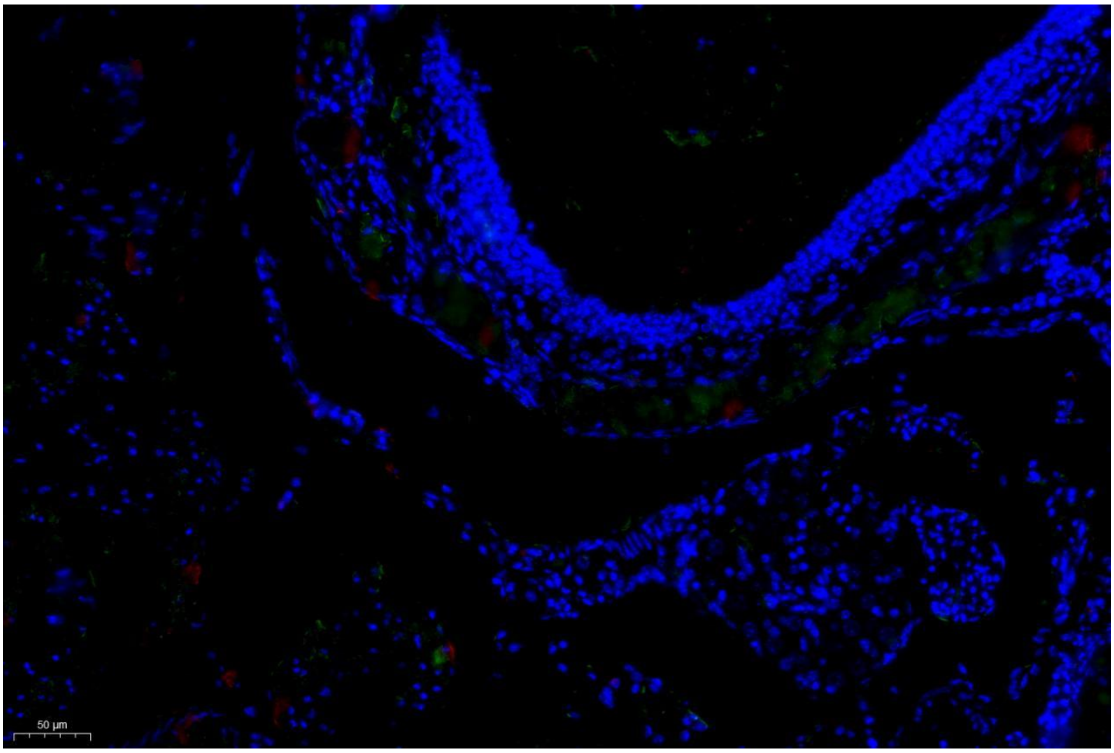
50 µm

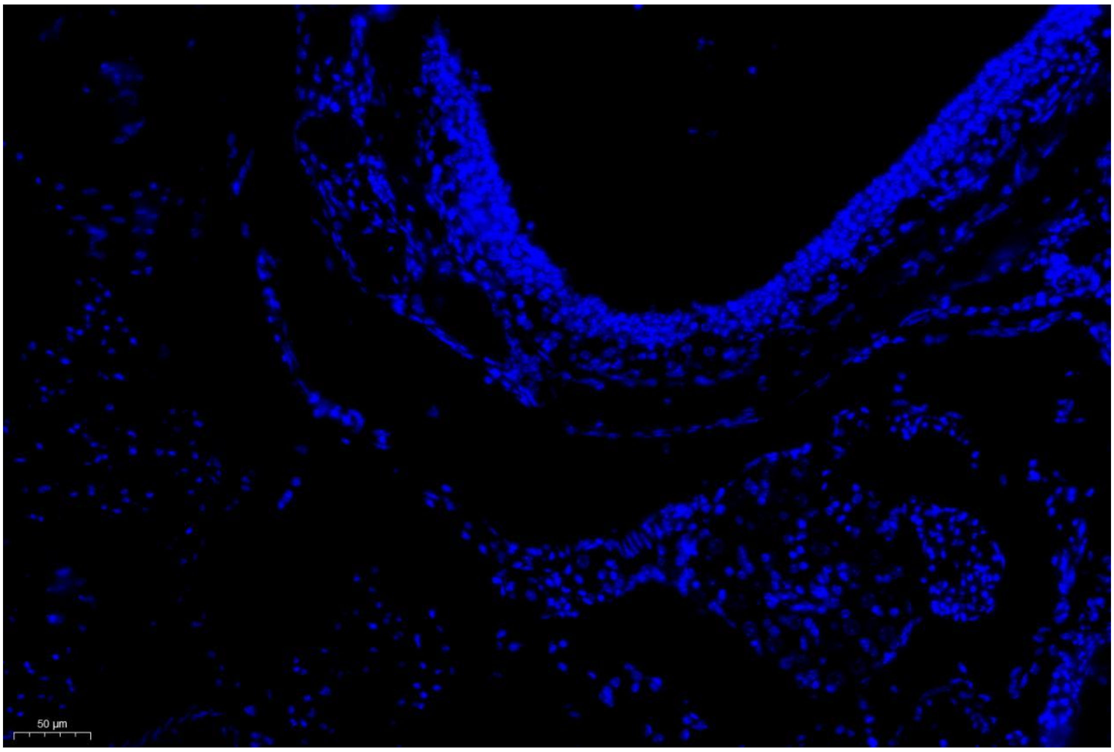
50 µm

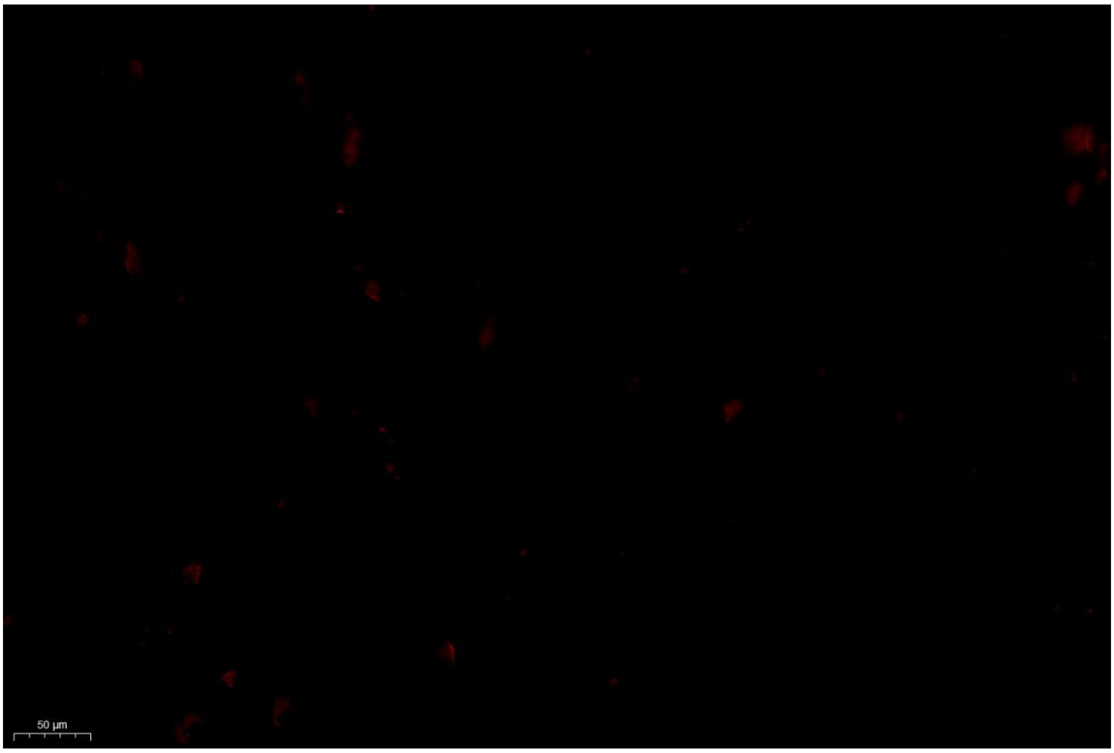
50 µm

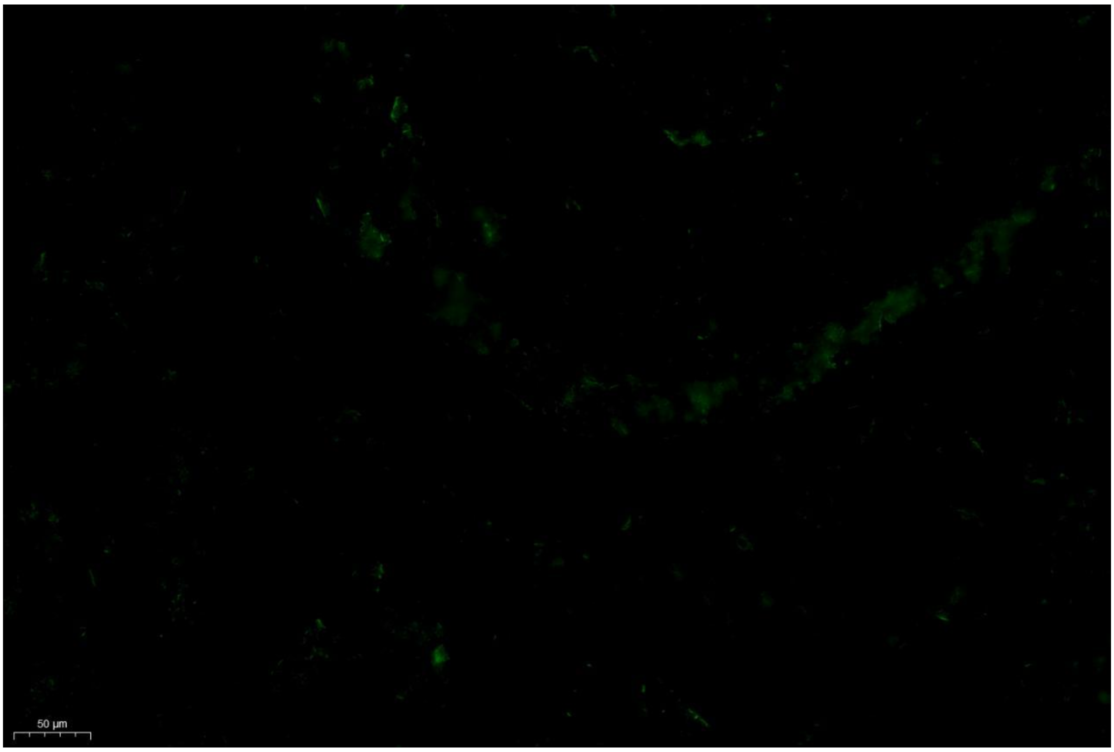
50 µm

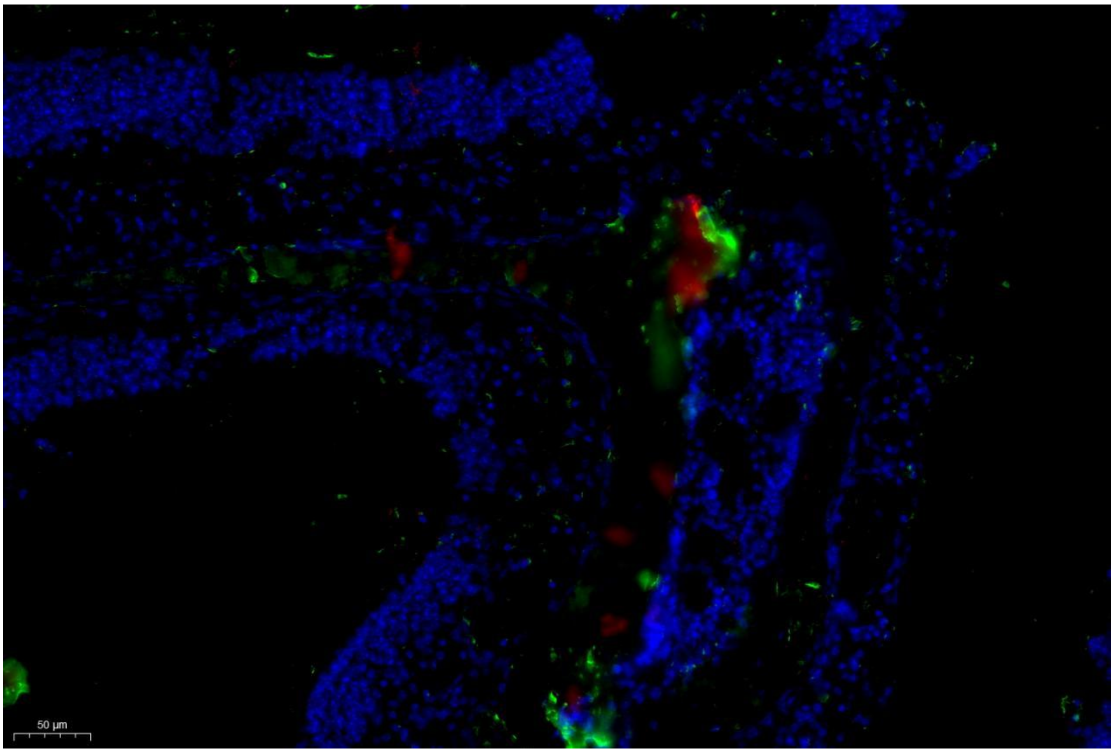
50 µm

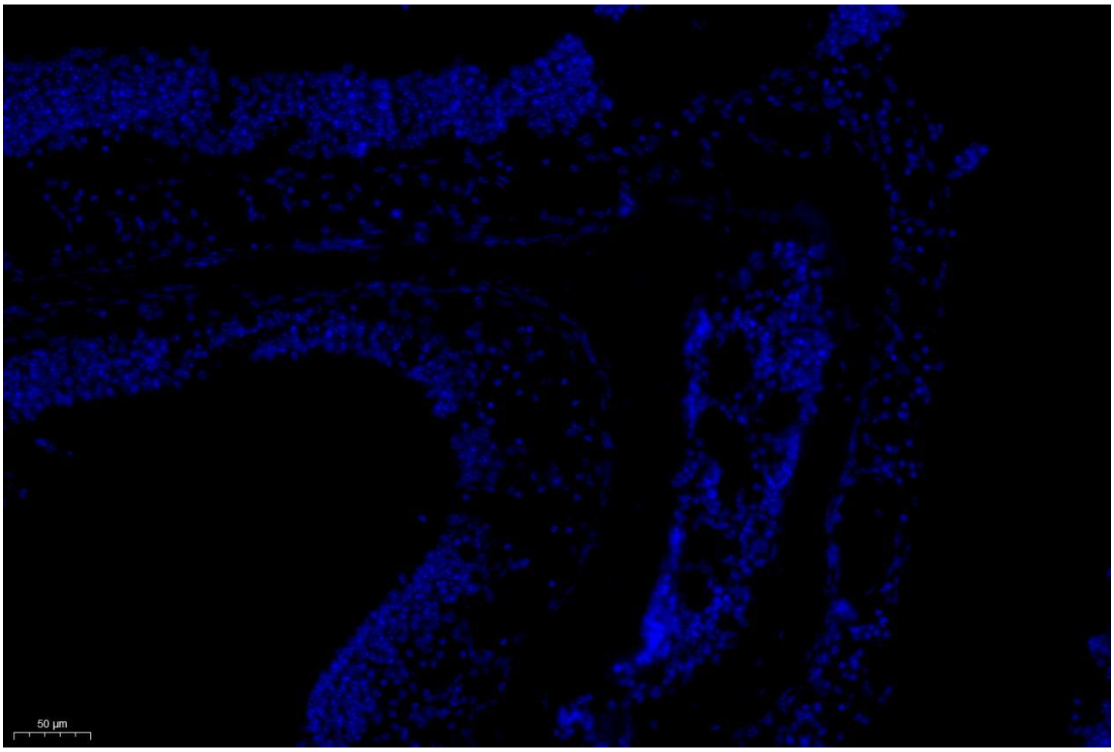
50 µm

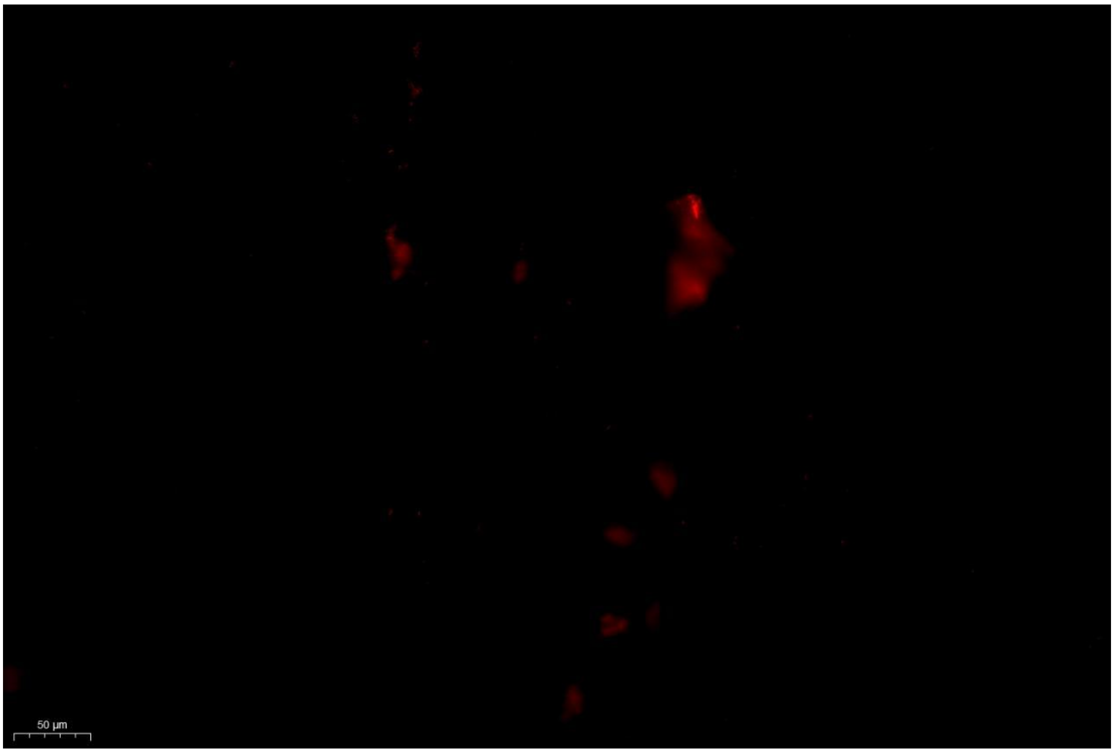
50 µm
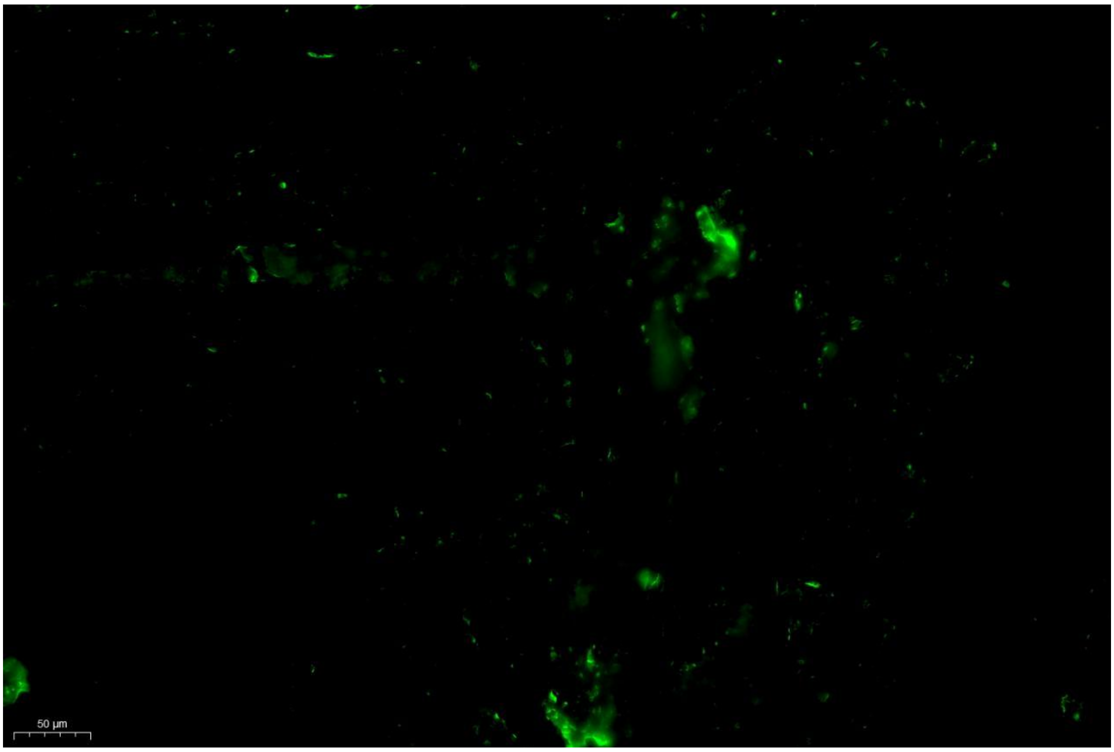
50 µm

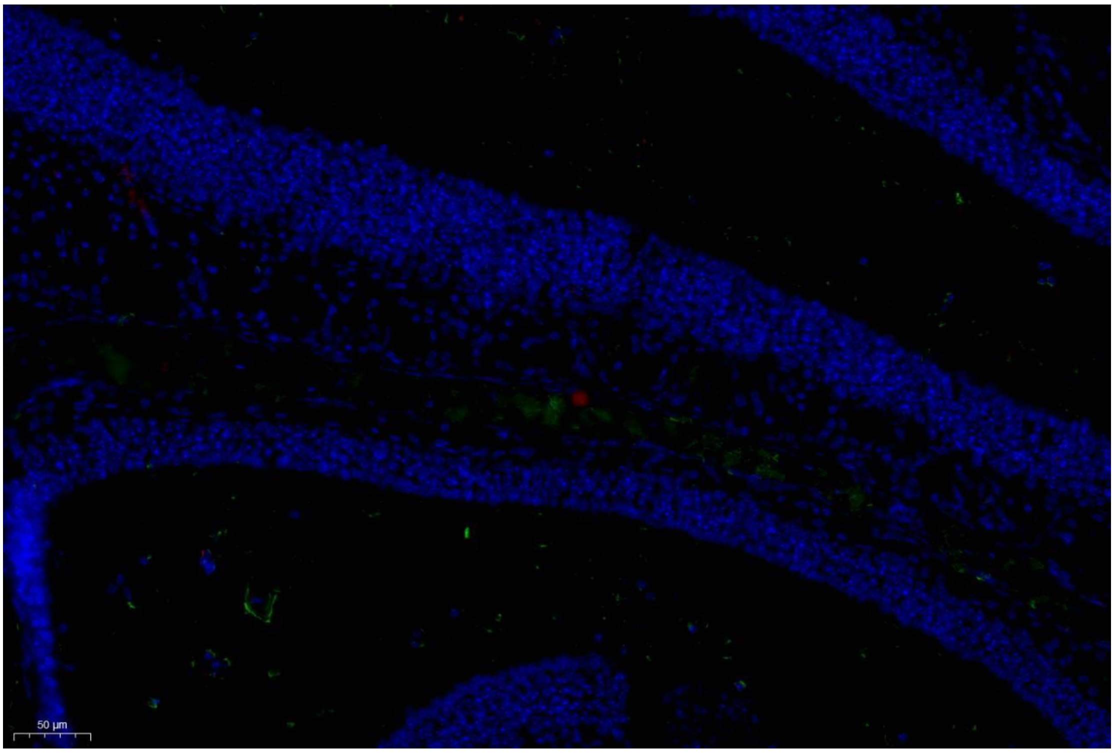
50 µm

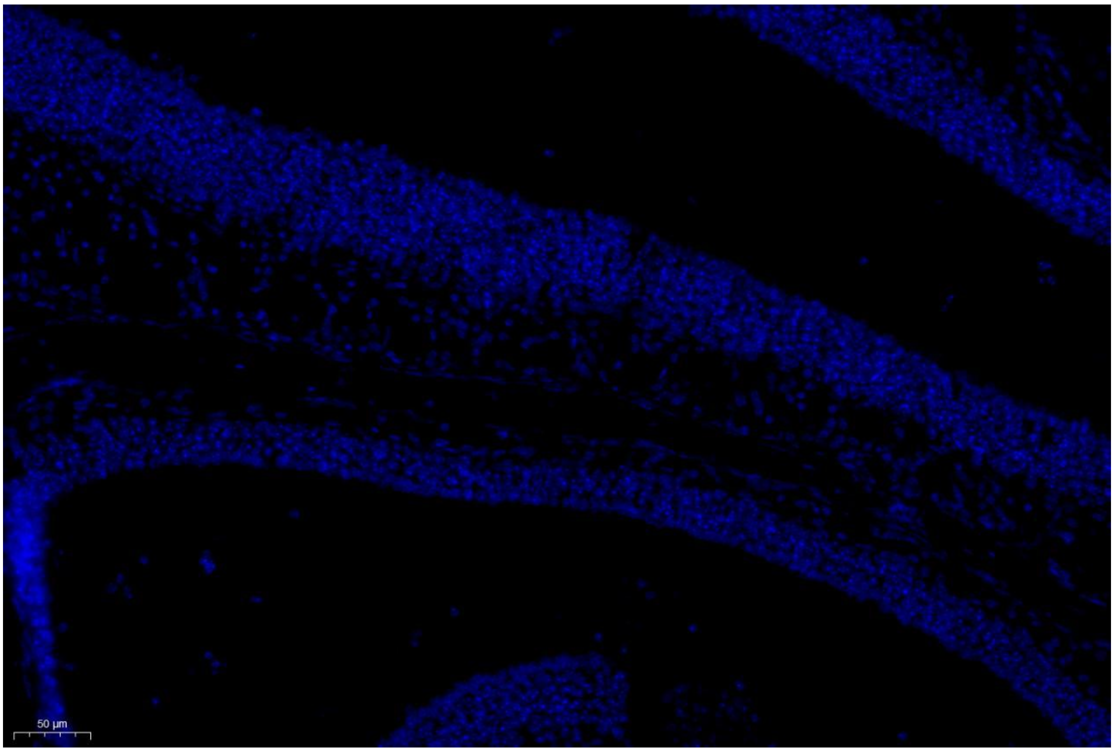
50 µm

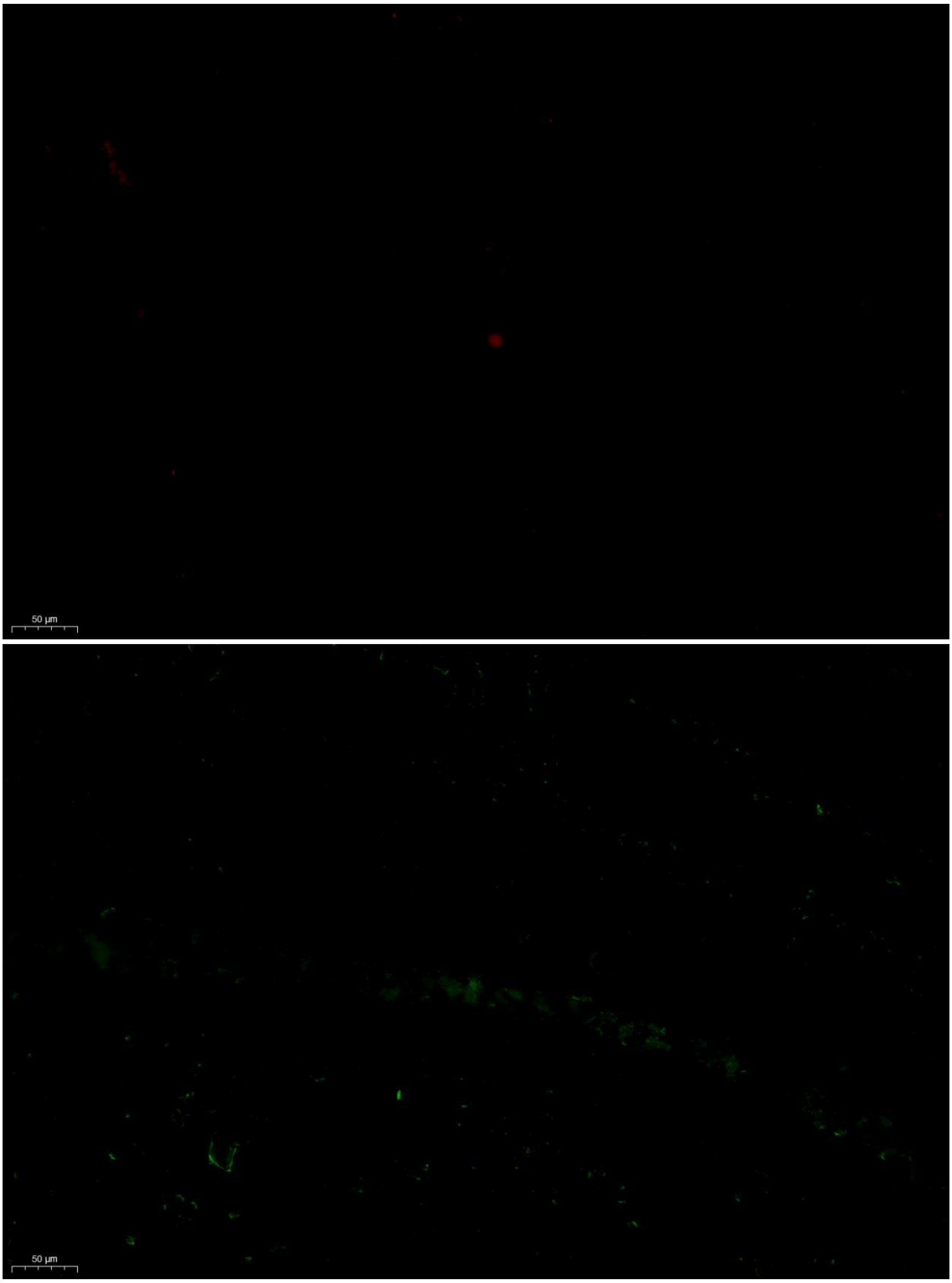


**FIG3D**

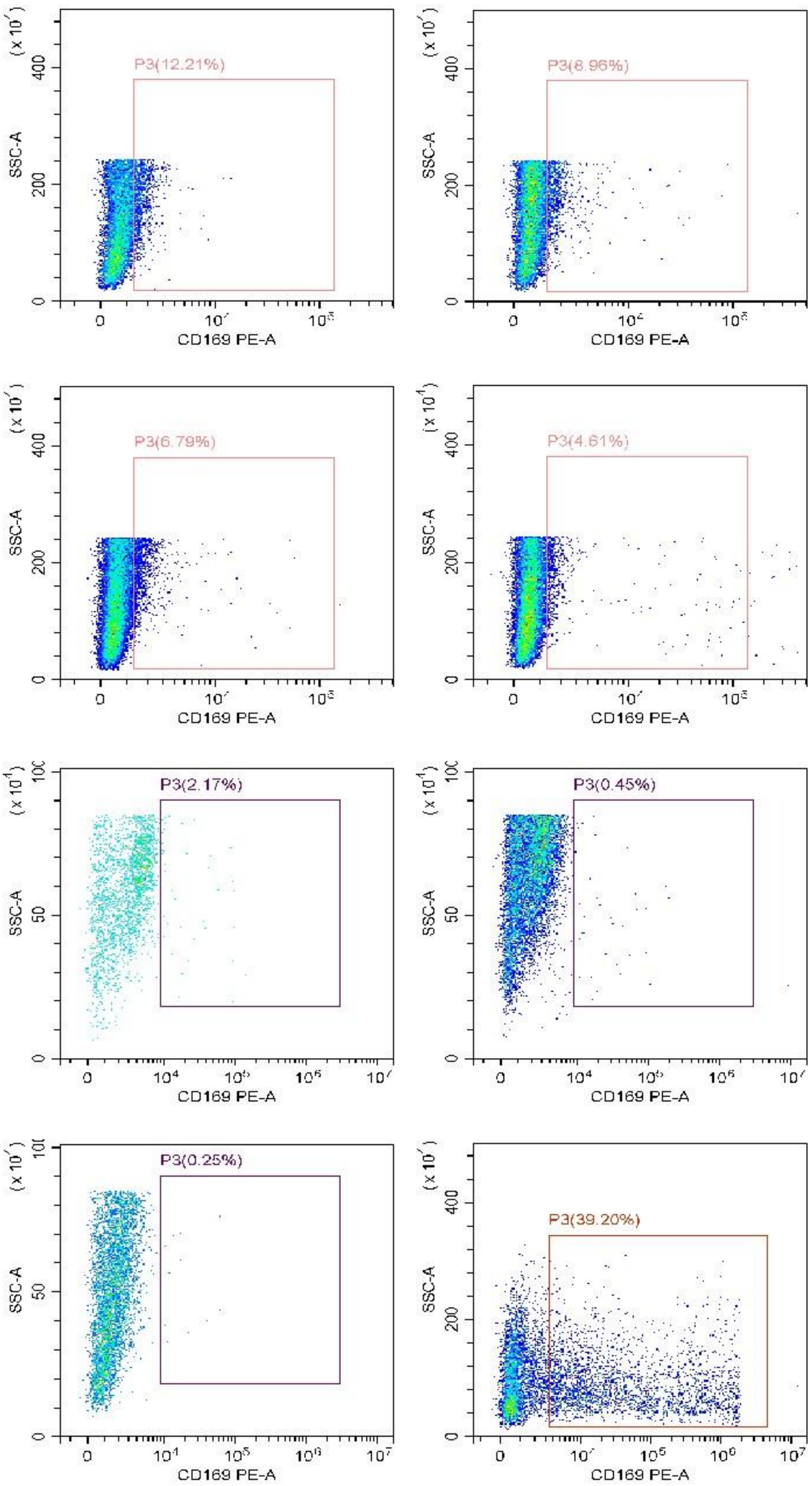
P3(12.21%)
P3(8.96%)
P3(6.79%)
P3(4.61%)
P3(2.17%)
P3(0.45%)
P3(0.25%)
P3(39.20%)
SSC-A
CD169 PE-A

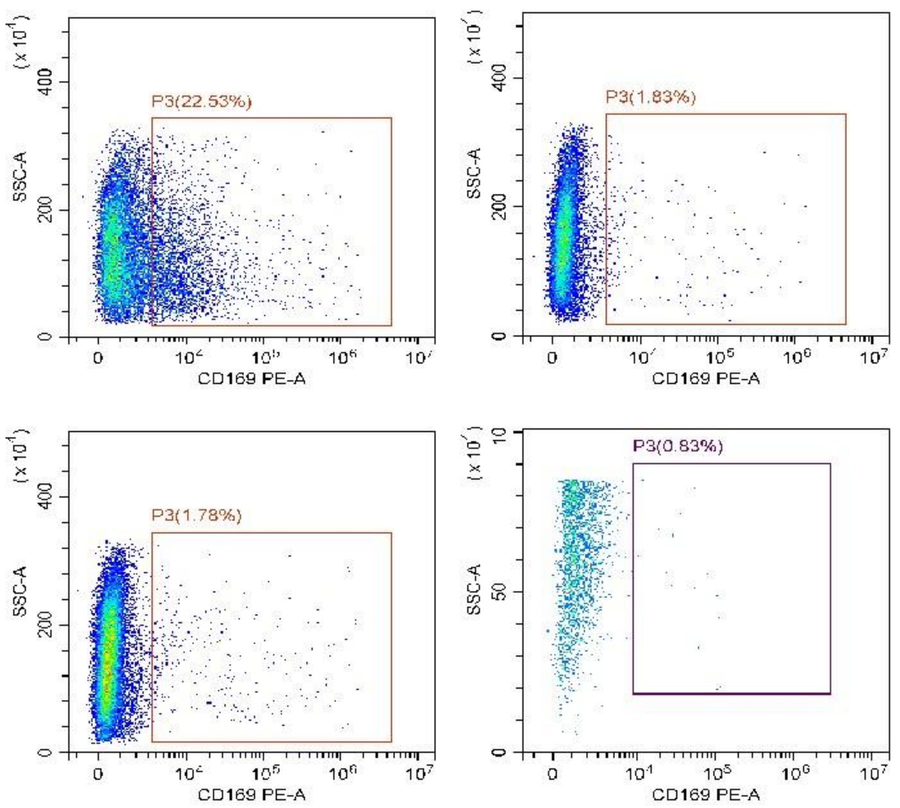
P3(22.53%)
P3(1.83%)
P3(1.78%)
P3(0.83%)
SSC-A
CD169 PE-A

# FIG4A

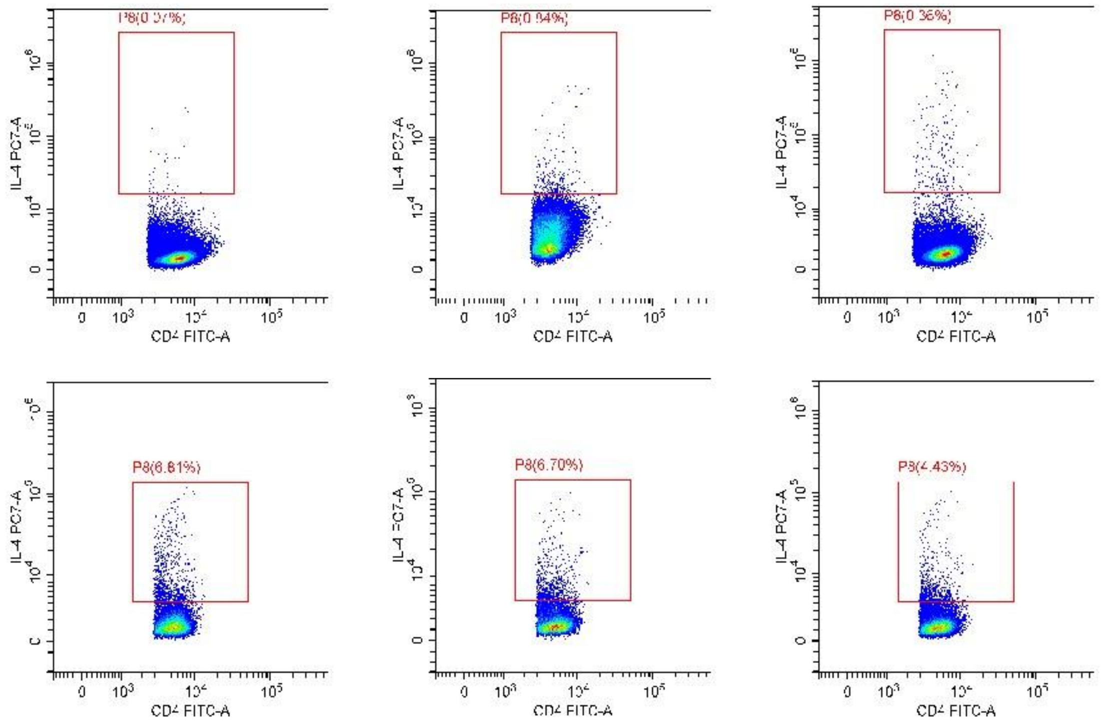
P8(0.36%)
P8(6.81%)
P8(6.70%)
P8(4.43%)
IL-4 PC7-A
CD4 FITC-A

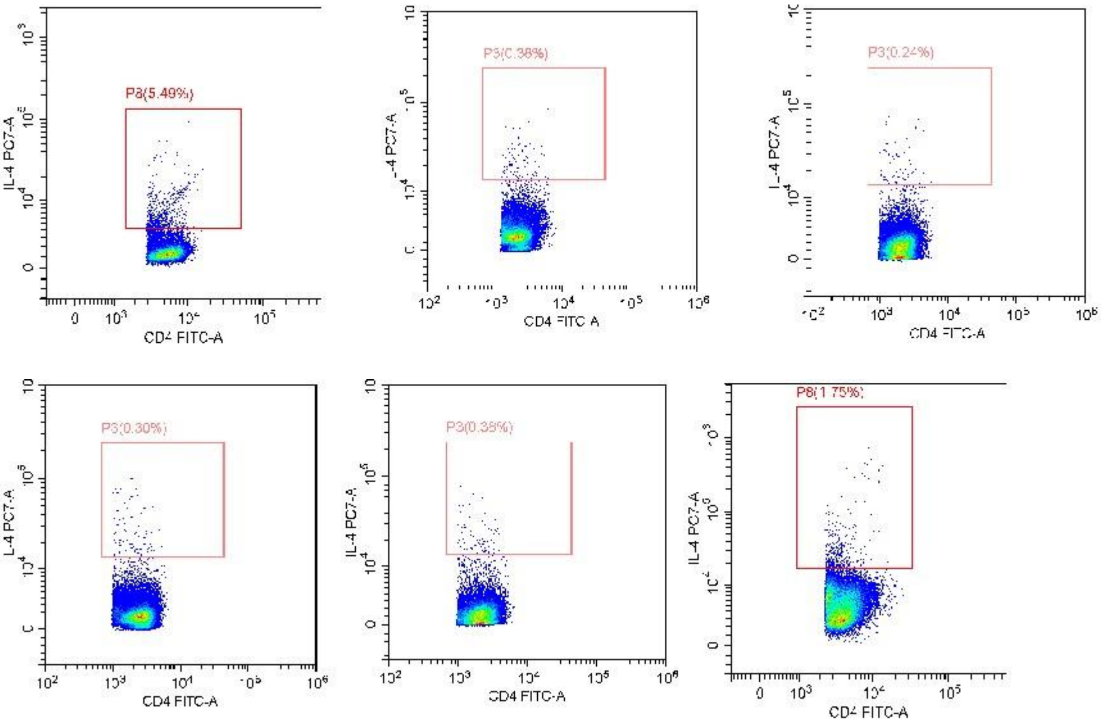
P8(5.49%)
P3(0.38%)
P3(0.24%)
P3(0.30%)
P3(0.38%)
P8(1.75%)
IL-4 PC7-A
CD4 FITC-A

# FIG4B

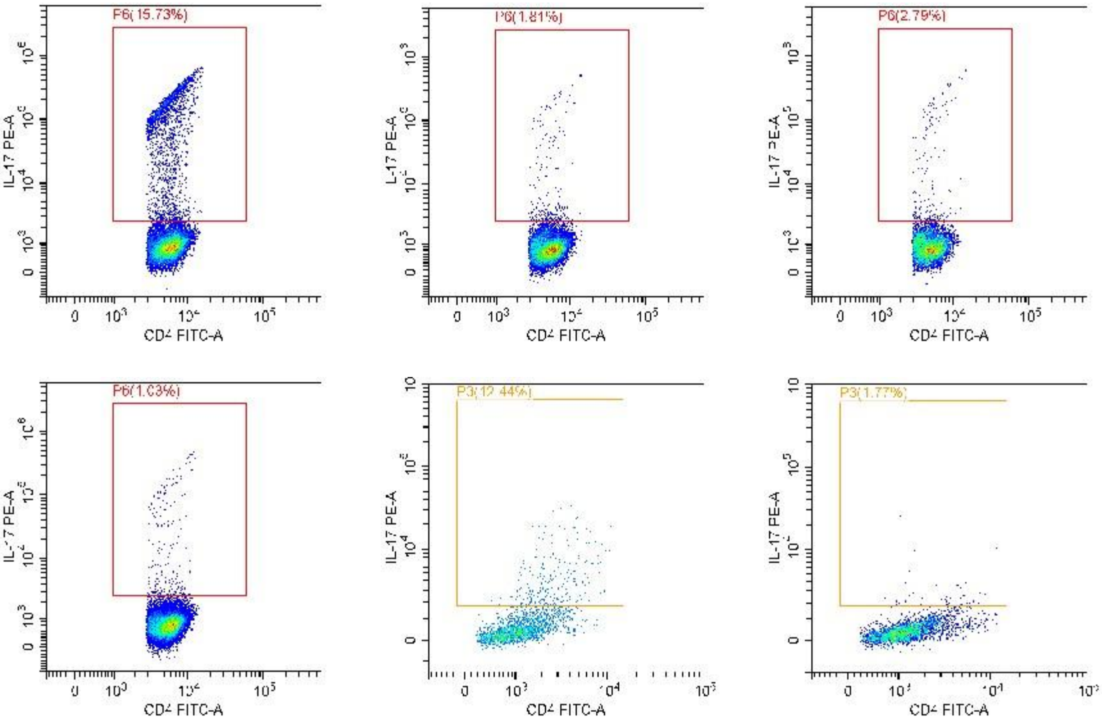
P6(15.73%)
P6(1.81%)
P6(2.79%)
P6(1.03%)
P3(12.44%)
P3(1.77%)
IL-17 PE-A
CD4 FITC-A

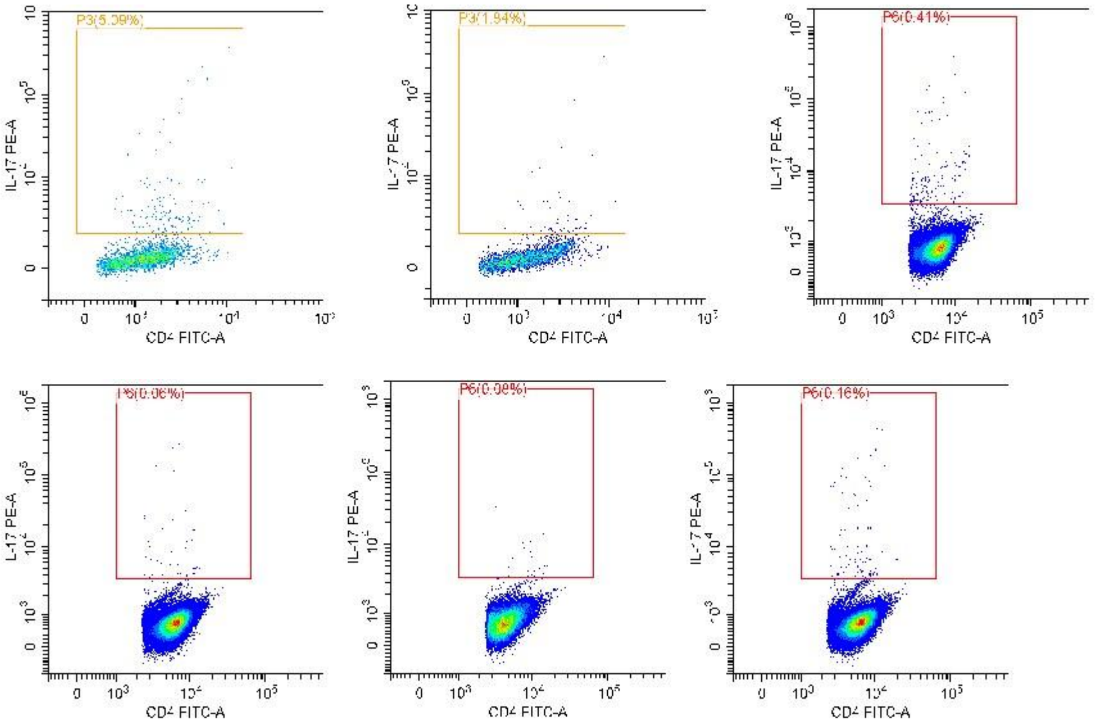


# FIG5A

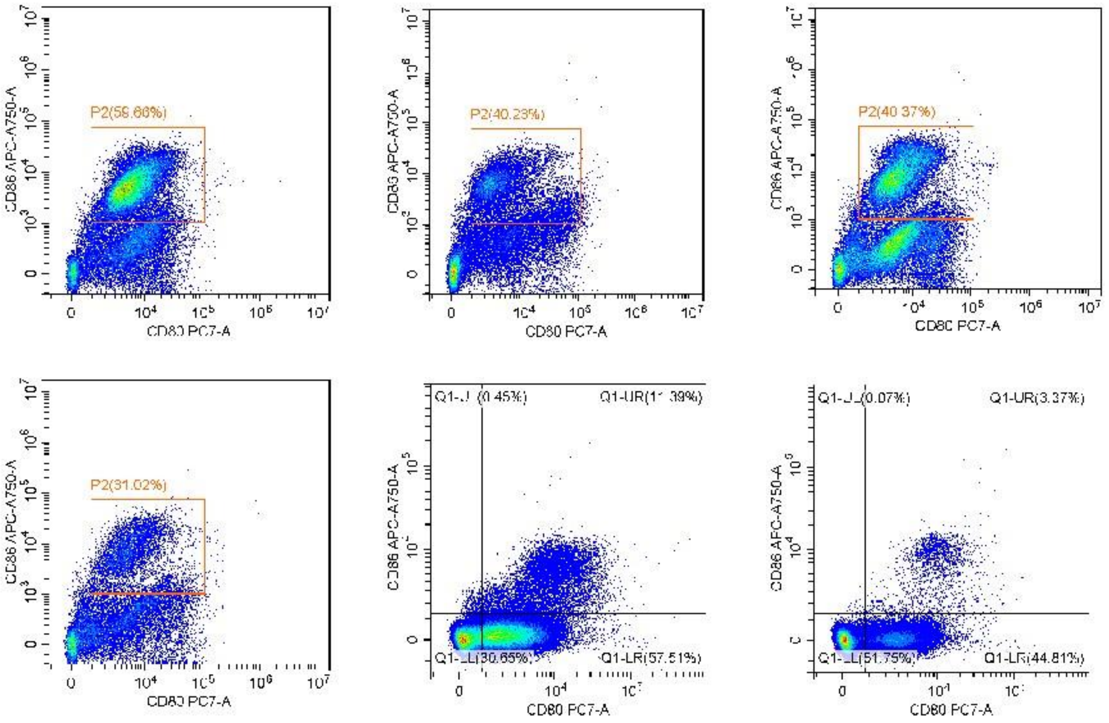

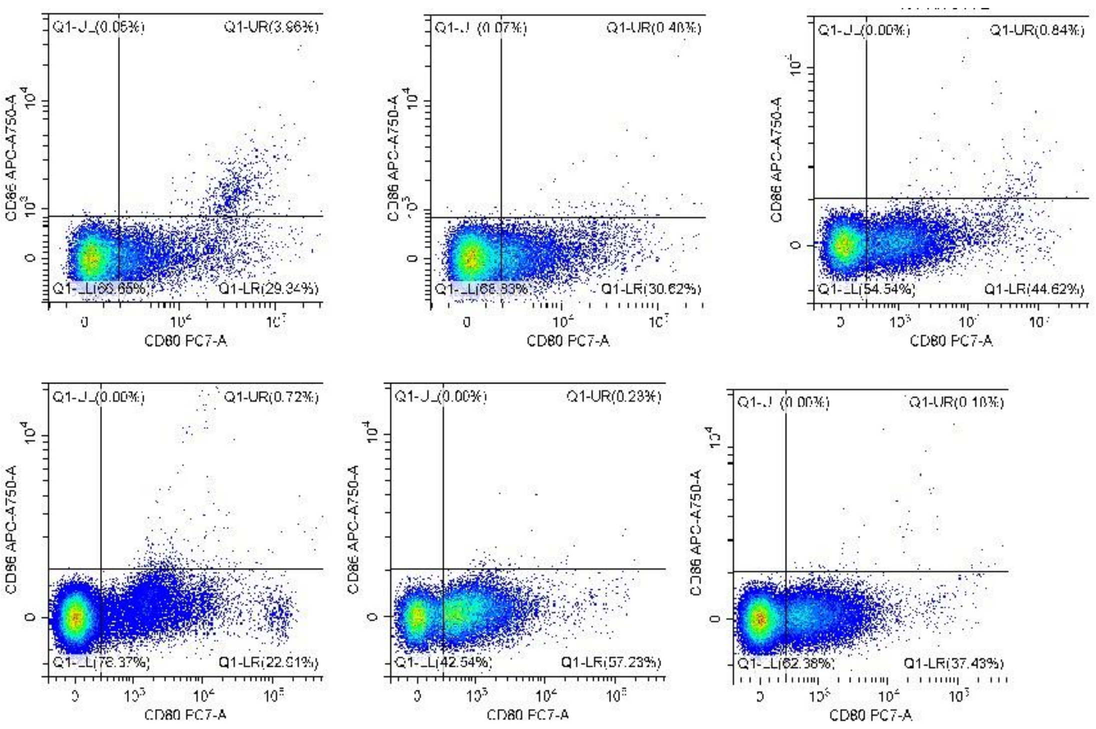


# FIG5B

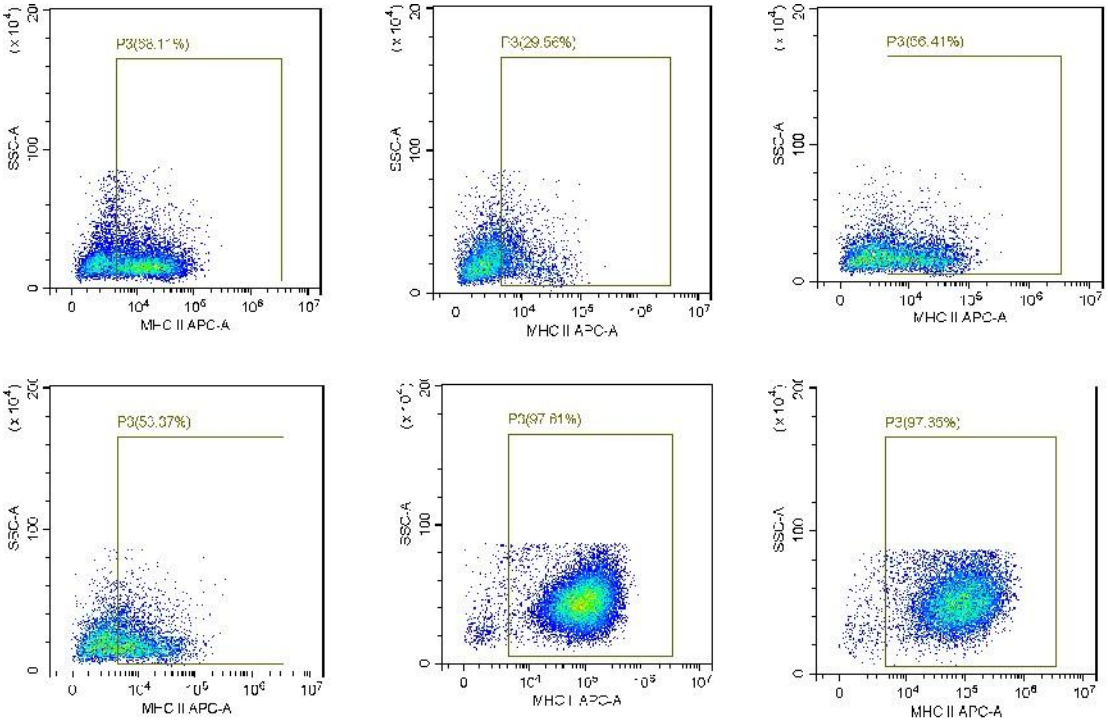

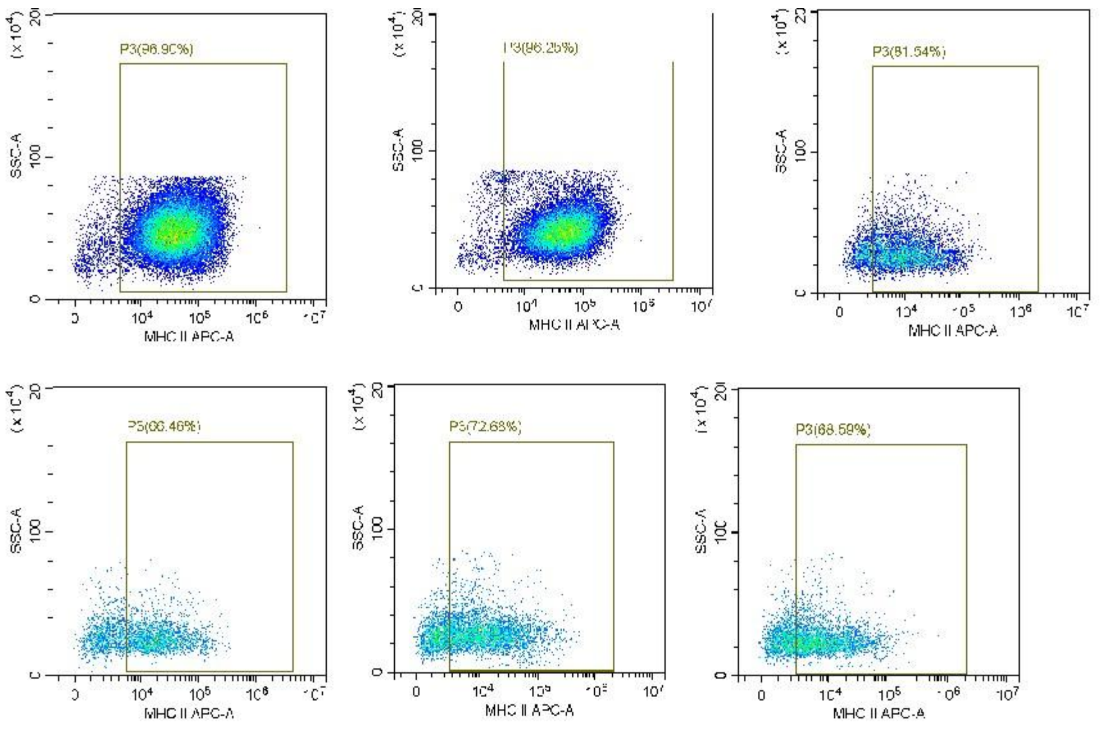
SSC-A
MHC II APC-A
P3

# FIG5C

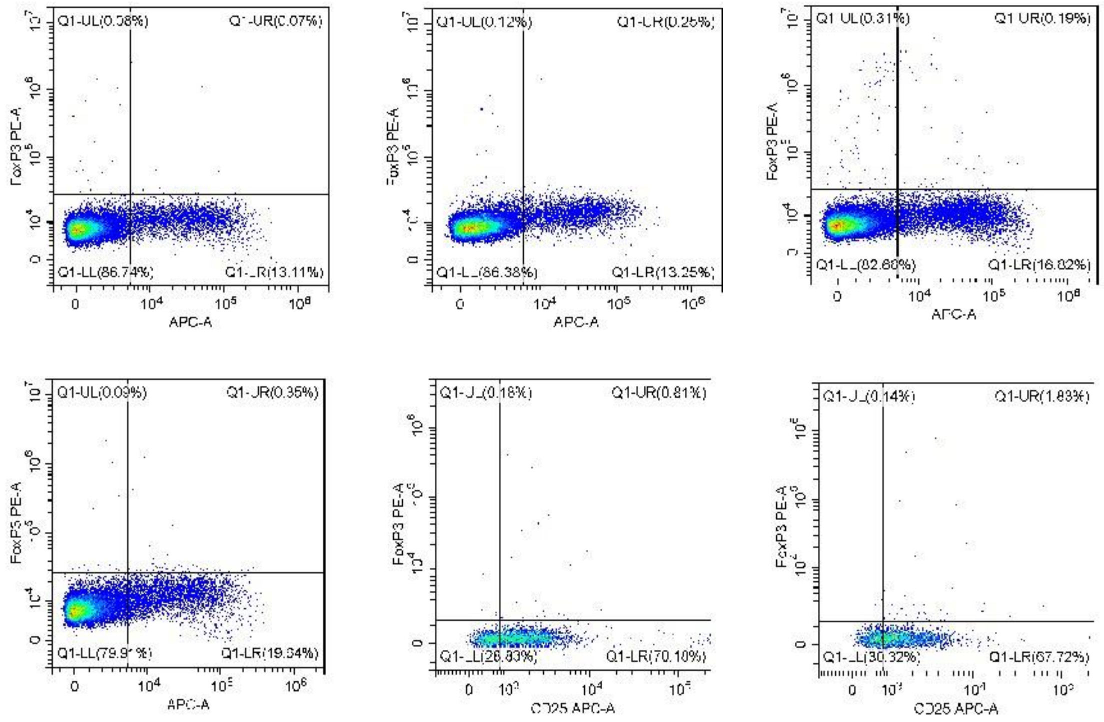
FoxP3 PE-A
APC-A
CD25 APC-A
Q1-UL
Q1-UR
Q1-LL
Q1-LR

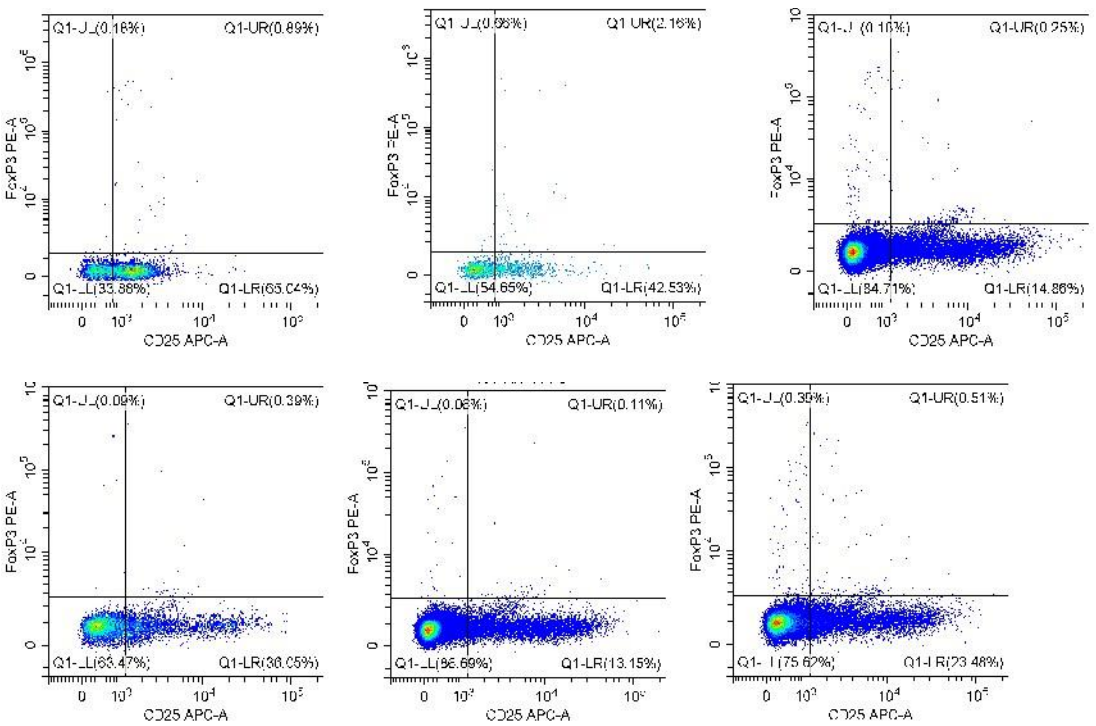
Q1-UL(0.16%)
Q1-UR(0.89%)
Q1-LL(33.88%)
Q1-LR(65.04%)
Q1-UR(2.16%)
Q1-LL(54.65%)
Q1-LR(42.53%)
Q1-UR(0.25%)
Q1-LL(84.71%)
Q1-LR(14.86%)
Q1-UL(0.09%)
Q1-UR(0.39%)
Q1-LL(63.47%)
Q1-LR(36.05%)
Q1-UL(0.06%)
Q1-UR(0.11%)
Q1-LL(86.69%)
Q1-LR(13.15%)
Q1-UL(0.39%)
Q1-UR(0.51%)
Q1-LR(23.48%)
FoxP3 PE-A
CD25 APC-A

# FIG6

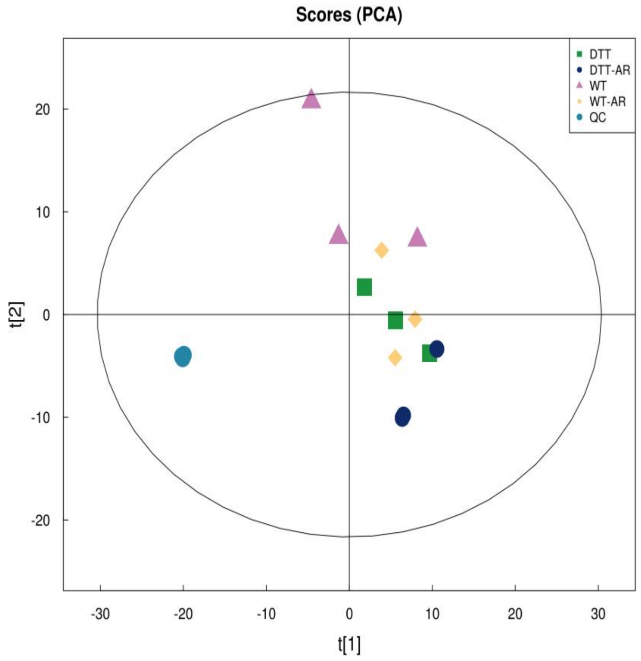
Scores (PCA)
DTT
DTT-AR
WT
WT-AR
QC
t[2]
20
10
0
-10
-20
-30
-20
-10
0
10
20
30
t[1]

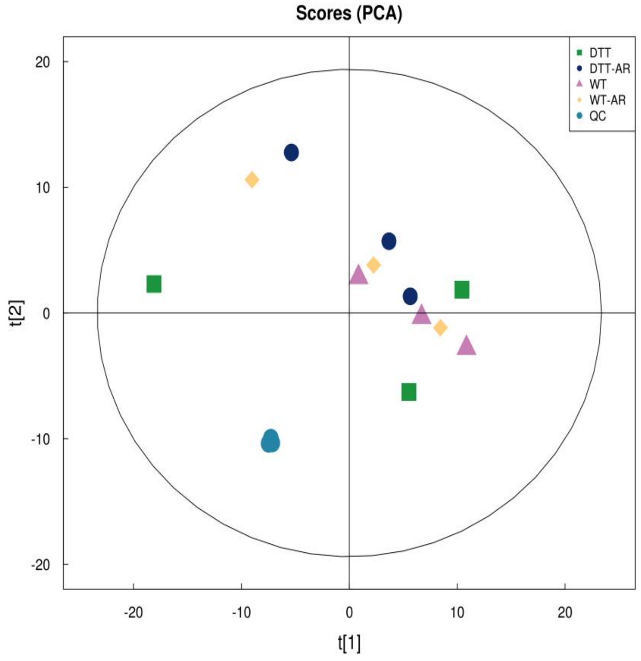
Scores (PCA)
DTT
DTT-AR
WT
WT-AR
QC
t[2]
20
10
0
-10
-20
-20
-10
0
10
20
t[1]

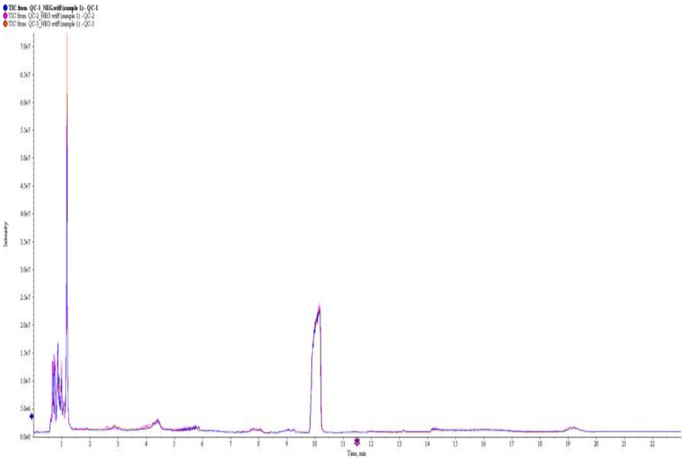

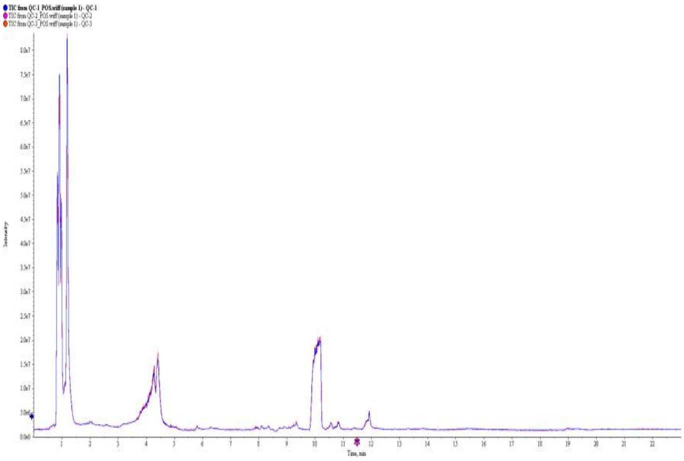

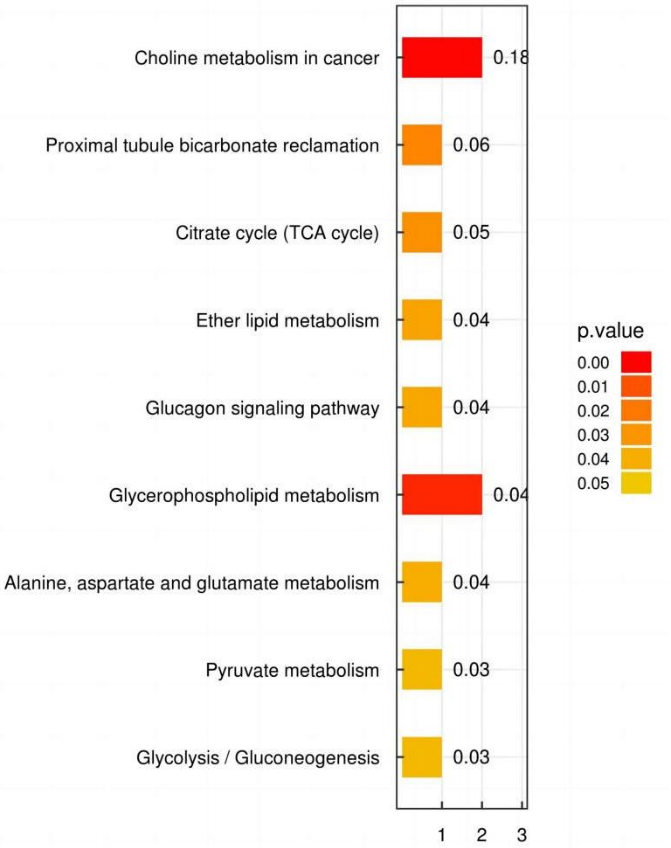
Choline metabolism in cancer
0.18
Proximal tubule bicarbonate reclamation
0.06
Citrate cycle (TCA cycle)
0.05
Ether lipid metabolism
0.04
Glucagon signaling pathway
0.04
Glycerophospholipid metabolism
0.04
Alanine, aspartate and glutamate metabolism
0.04
Pyruvate metabolism
0.03
Glycolysis / Gluconeogenesis
0.03
1
2
3
p.value
0.00
0.01
0.02
0.03
0.04
0.05

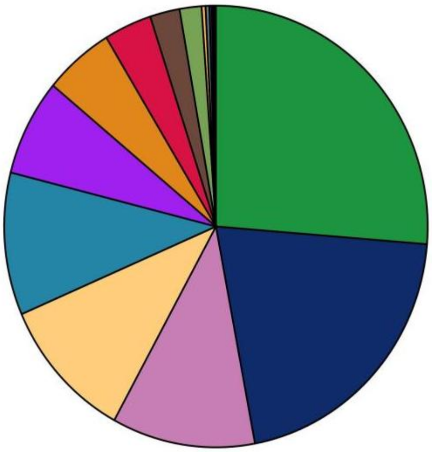

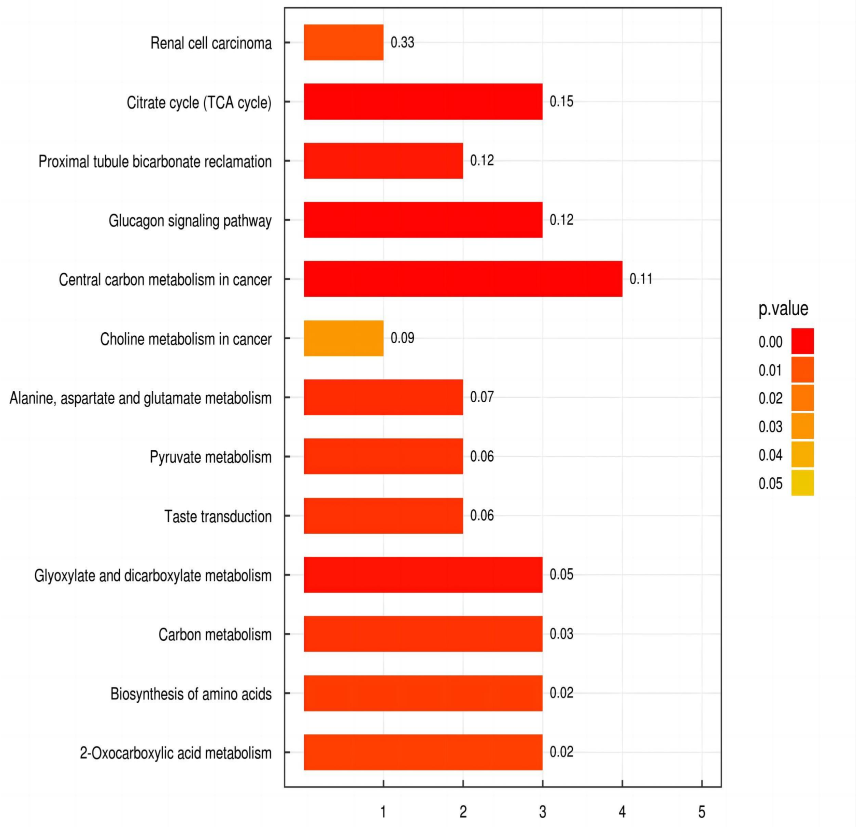
Renal cell carcinoma
0.33
Citrate cycle (TCA cycle)
0.15
Proximal tubule bicarbonate reclamation
0.12
Glucagon signaling pathway
0.12
Central carbon metabolism in cancer
0.11
Choline metabolism in cancer
0.09
Alanine, aspartate and glutamate metabolism
0.07
Pyruvate metabolism
0.06
Taste transduction
0.06
Glyoxylate and dicarboxylate metabolism
0.05
Carbon metabolism
0.03
Biosynthesis of amino acids
0.02
2-Oxocarboxylic acid metabolism
0.02
1
2
3
4
5
p.value
0.00
0.01
0.02
0.03
0.04
0.05

# FIG7

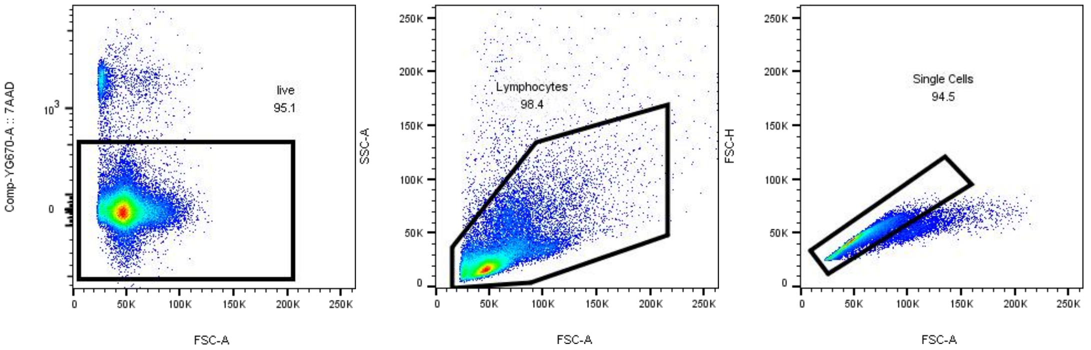
Comp-YG670-A :: 7AAD
live
95.1
FSC-A
SSC-A
Lymphocytes
98.4
FSC-A
FSC-H
Single Cells
94.5
FSC-A

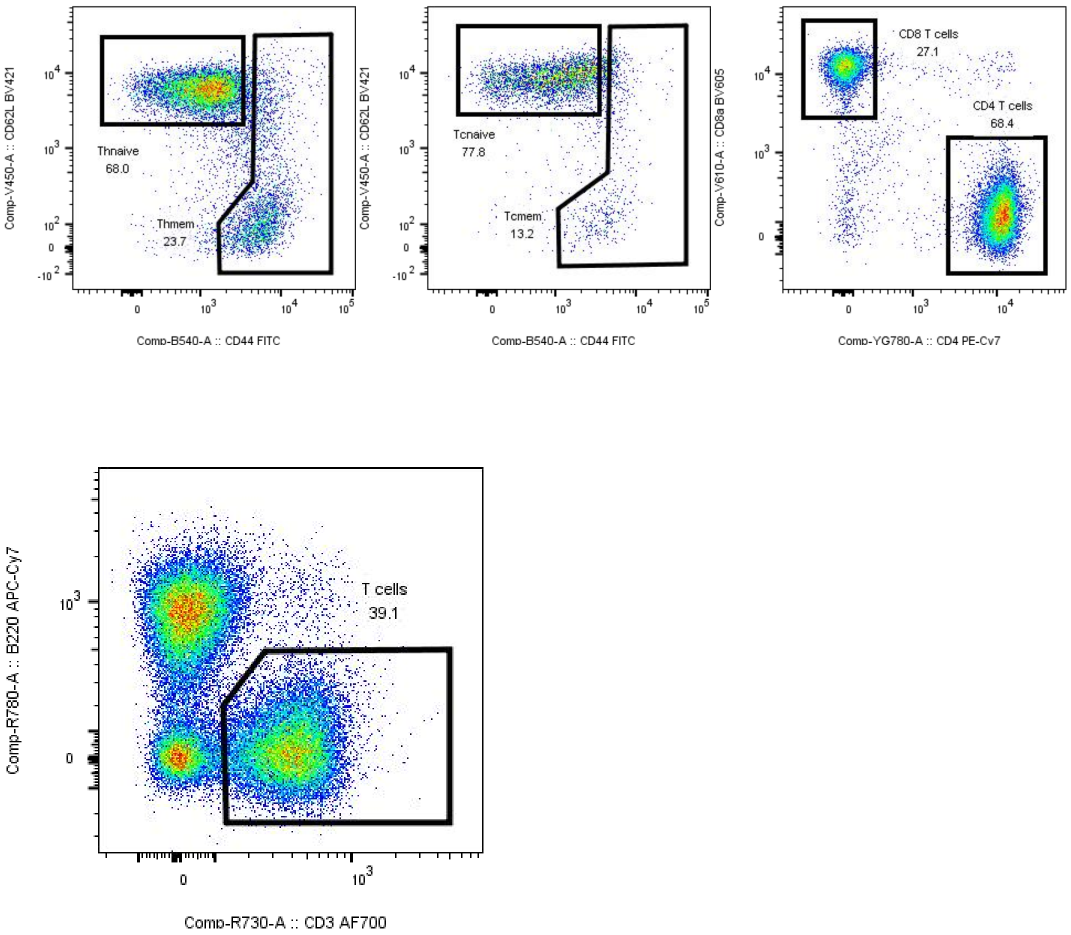

Comp-V450-A :: CD62L BV421
Thnaive
68.0
Thmem
23.7
Comp-B540-A :: CD44 FITC
Comp-V450-A :: CD62L BV421
Tcnaive
77.8
Tcmem
13.2
Comp-B540-A :: CD44 FITC
Comp-V610-A :: CD8a BV605
CD8 T cells
27.1
CD4 T cells
68.4
Comp-YG780-A :: CD4 PE-Cy7
Comp-R780-A :: B220 APC-Cy7
T cells
39.1
Comp-R730-A :: CD3 AF700


# FIG8

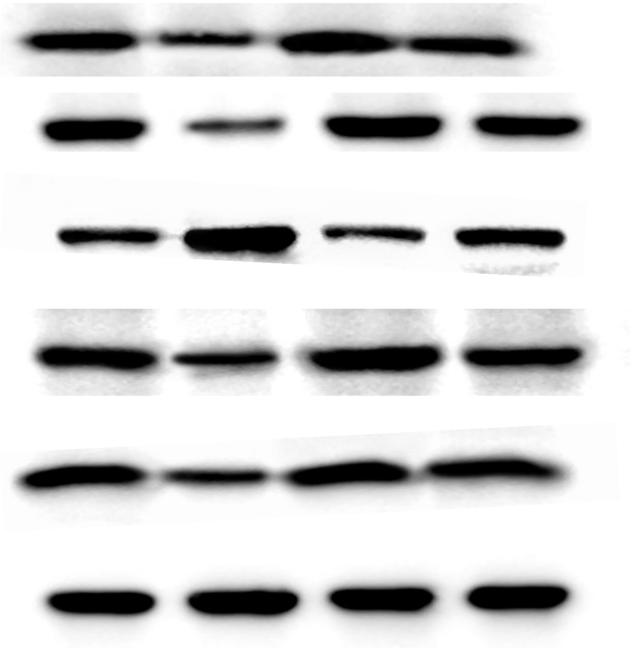

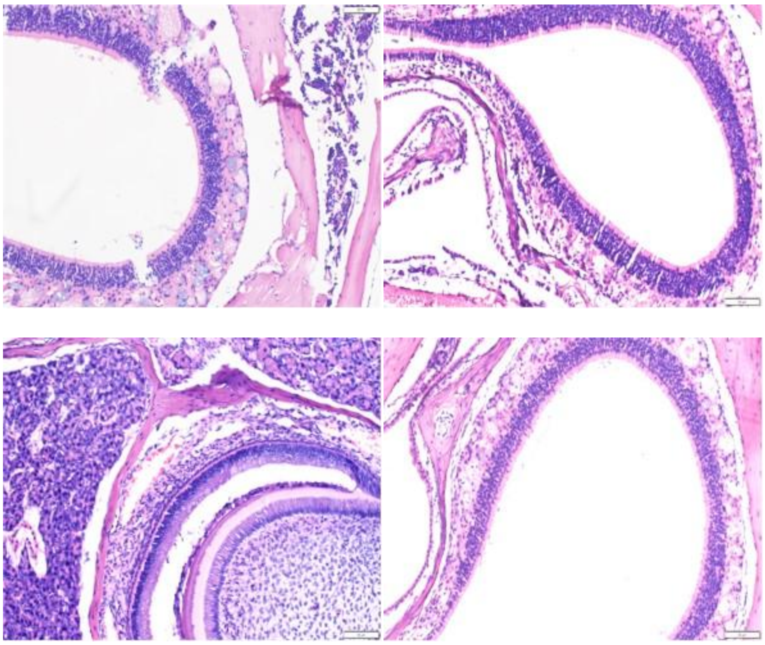

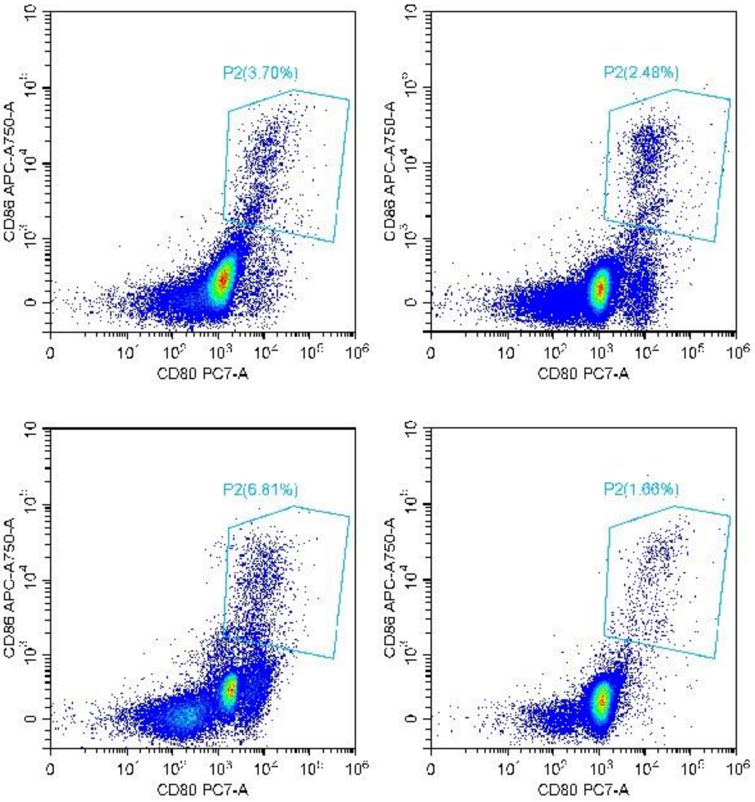
P2(3.70%)
CD86 APC-A750-A
CD80 PC7-A
P2(2.48%)
CD86 APC-A750-A
CD80 PC7-A
P2(6.81%)
CD86 APC-A750-A
CD80 PC7-A
P2(1.66%)
CD86 APC-A750-A
CD80 PC7-A

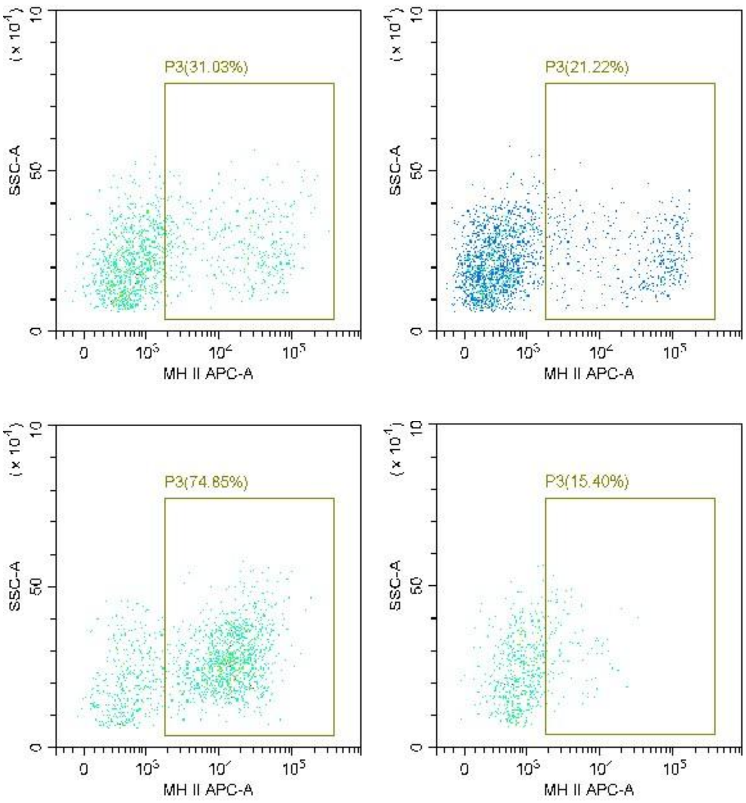
P3(31.03%)
P3(21.22%)
P3(74.85%)
P3(15.40%)
SSC-A
(x 10⁴)
MH II APC-A
0
50
10

FIG9

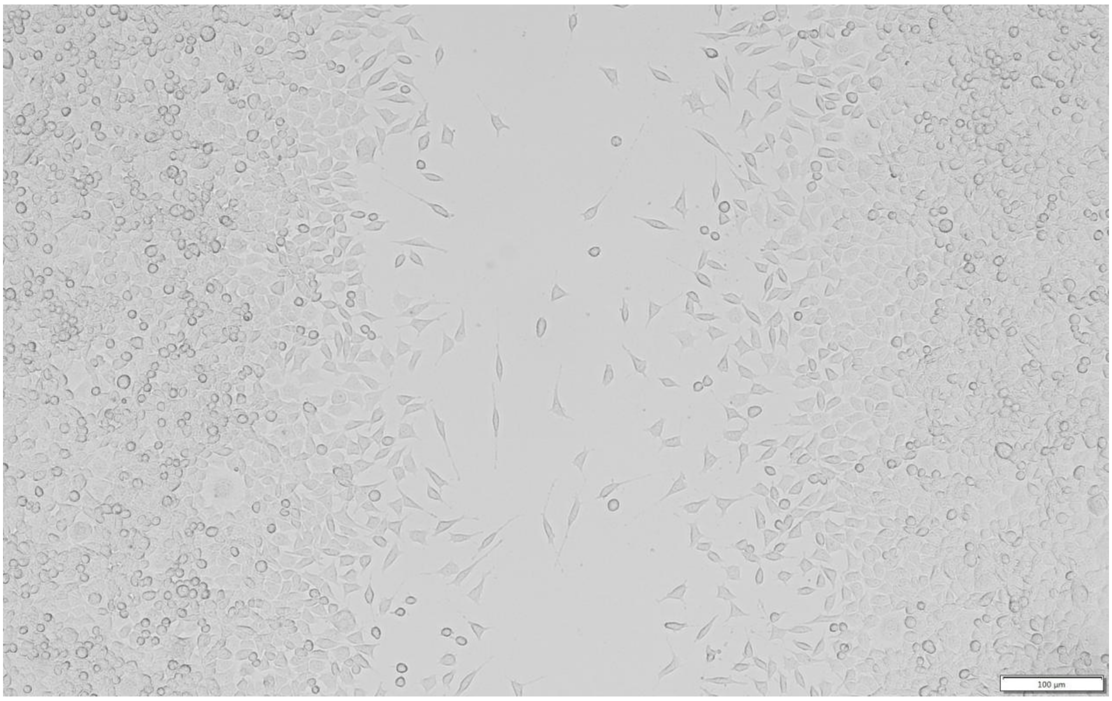
100 μm

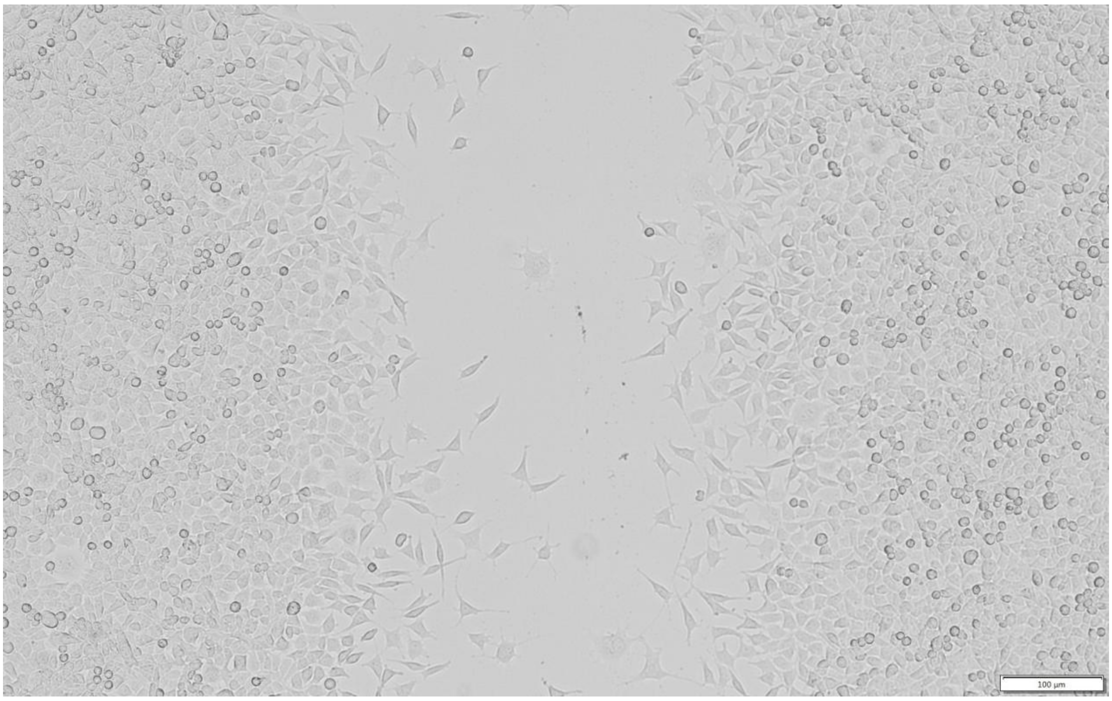
100 µm

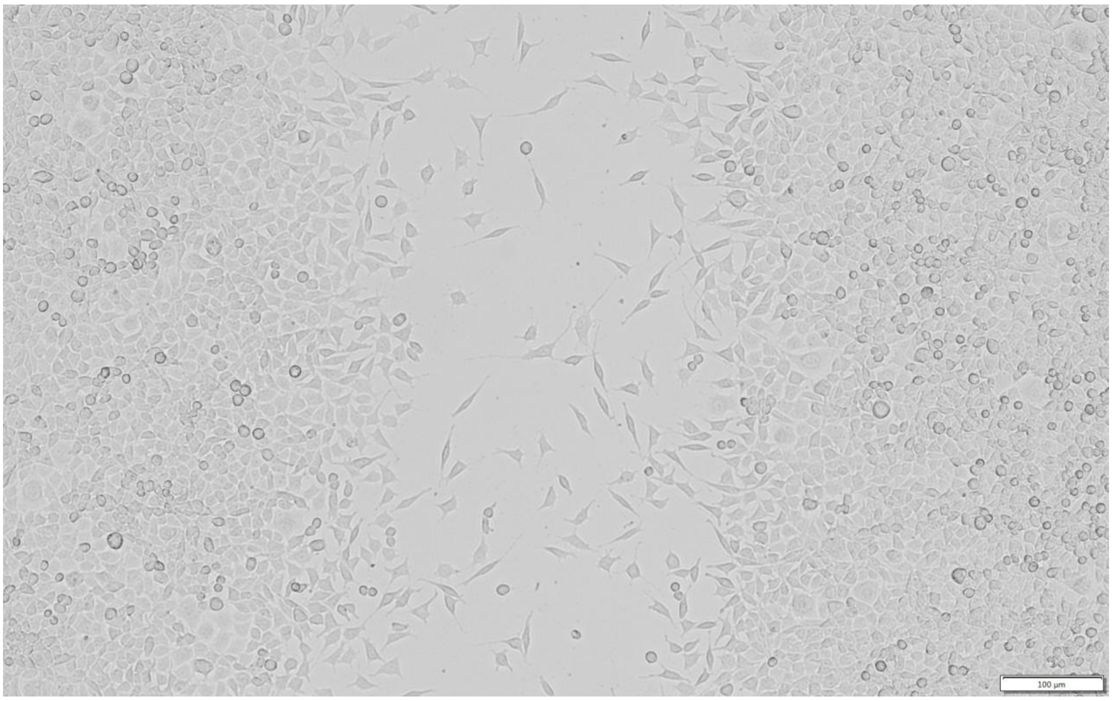
100 µm

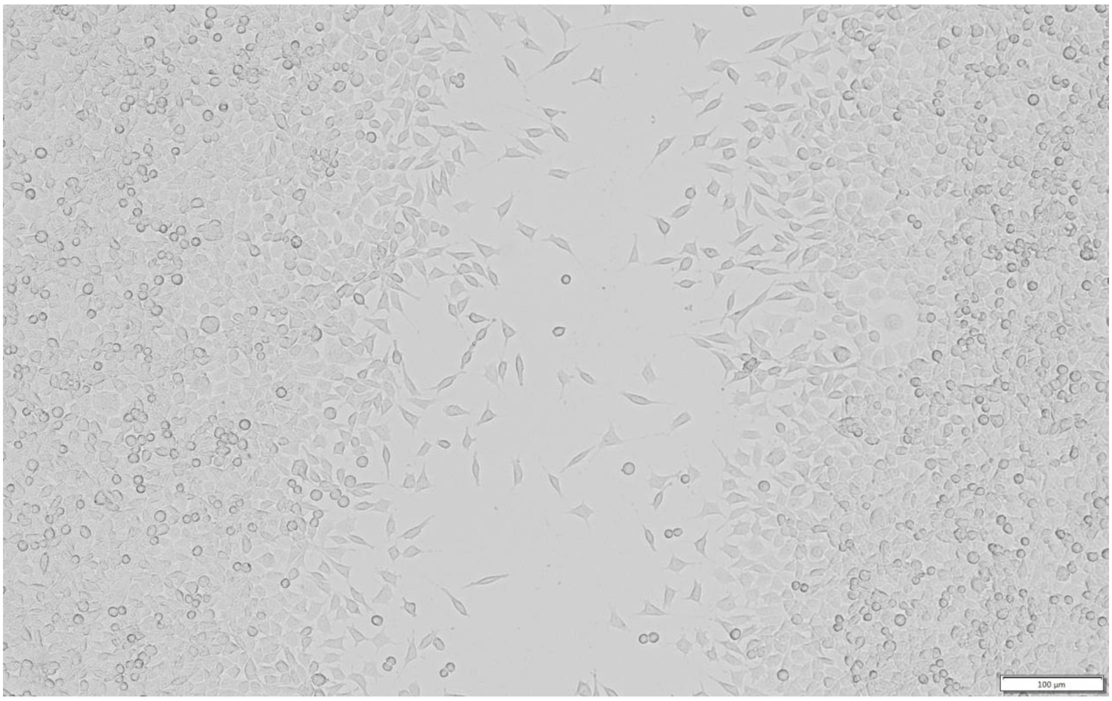
100 µm

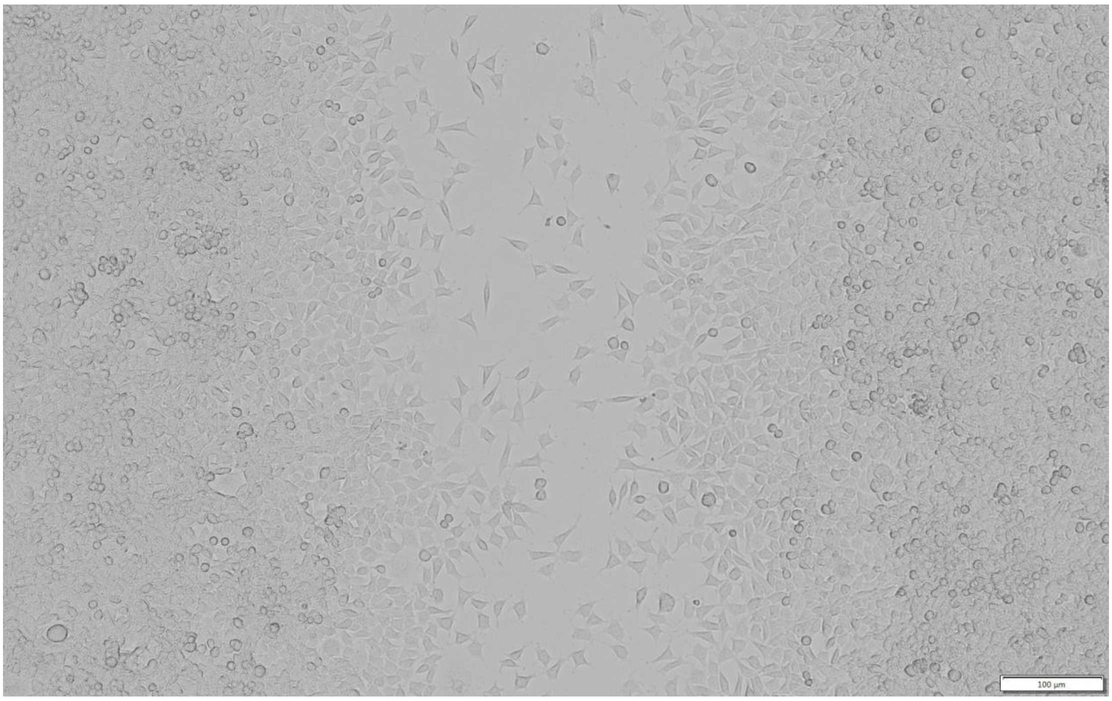
100 µm

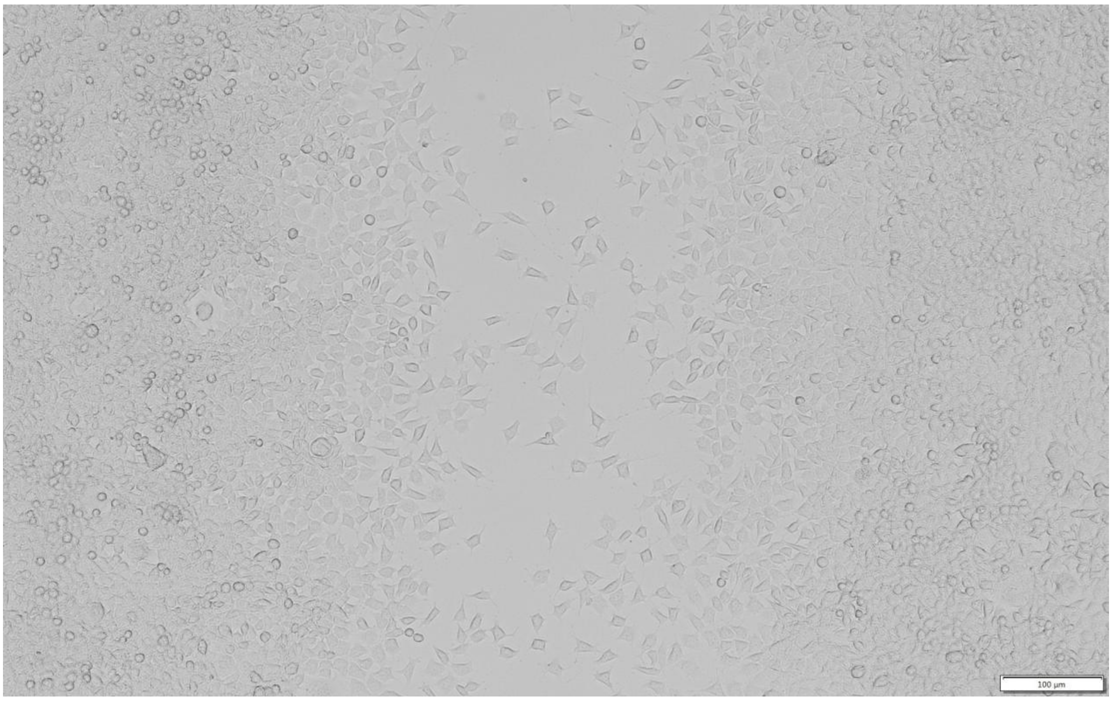
100 µm

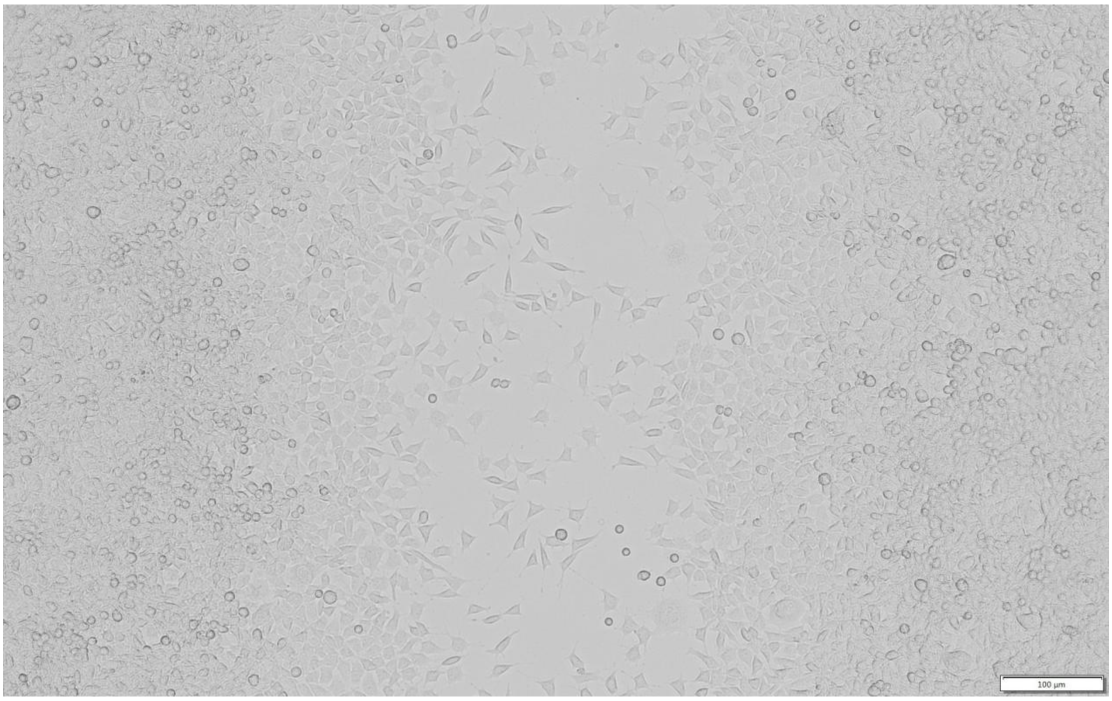
100 µm

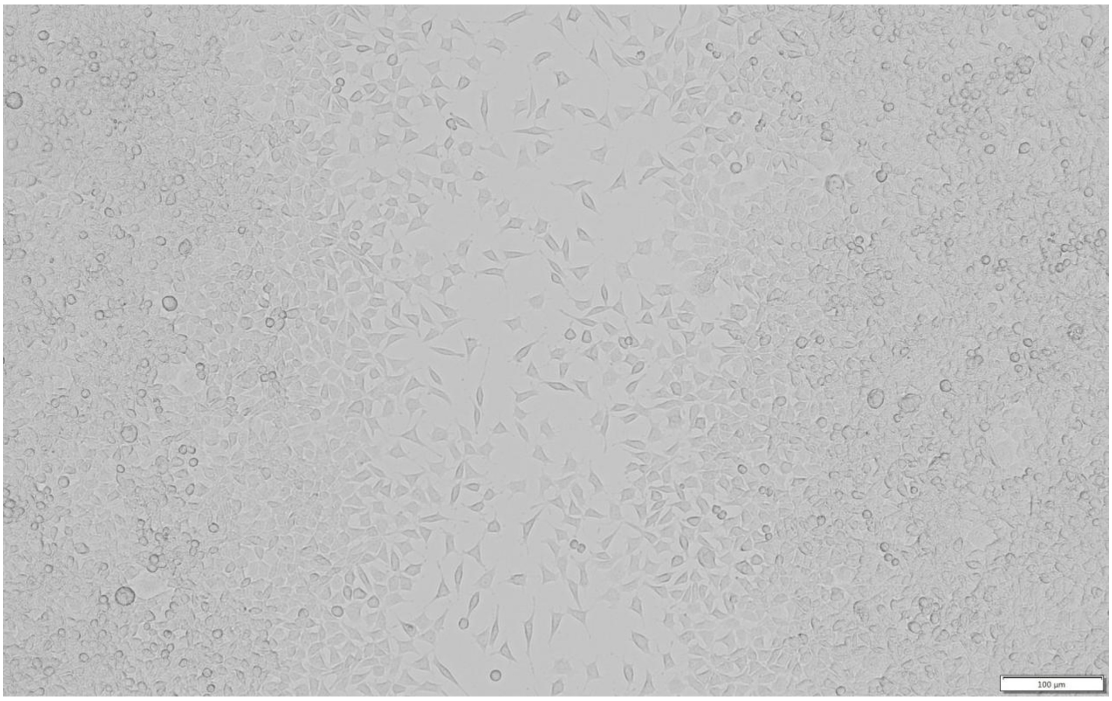
100 µm

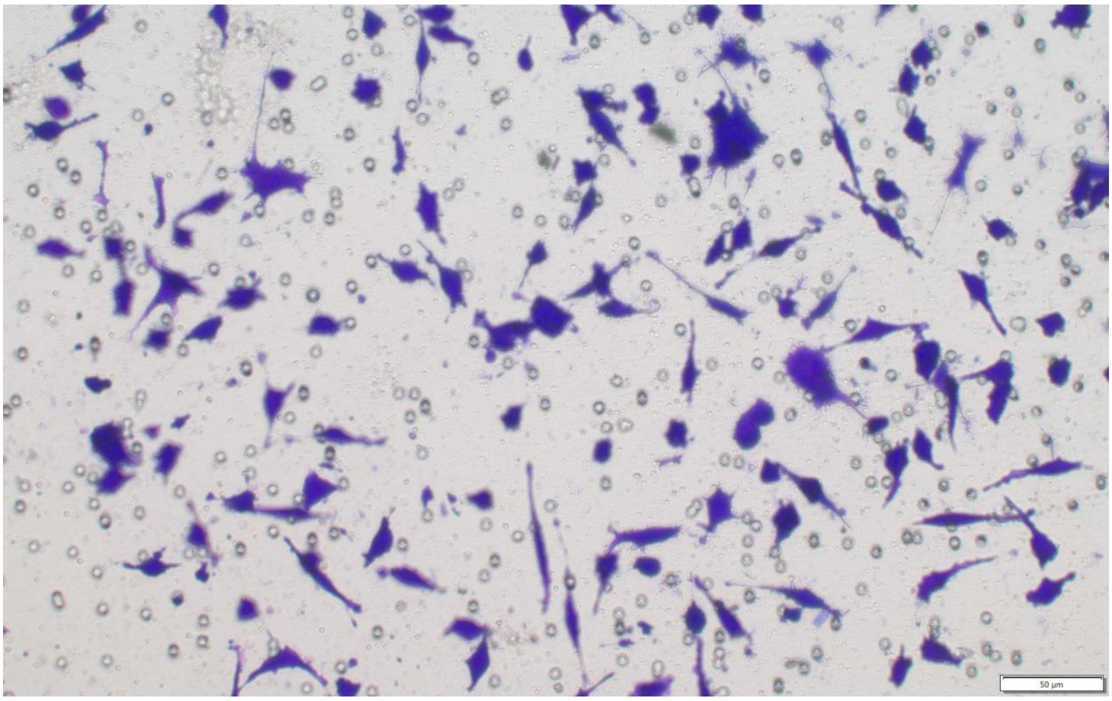
50 µm

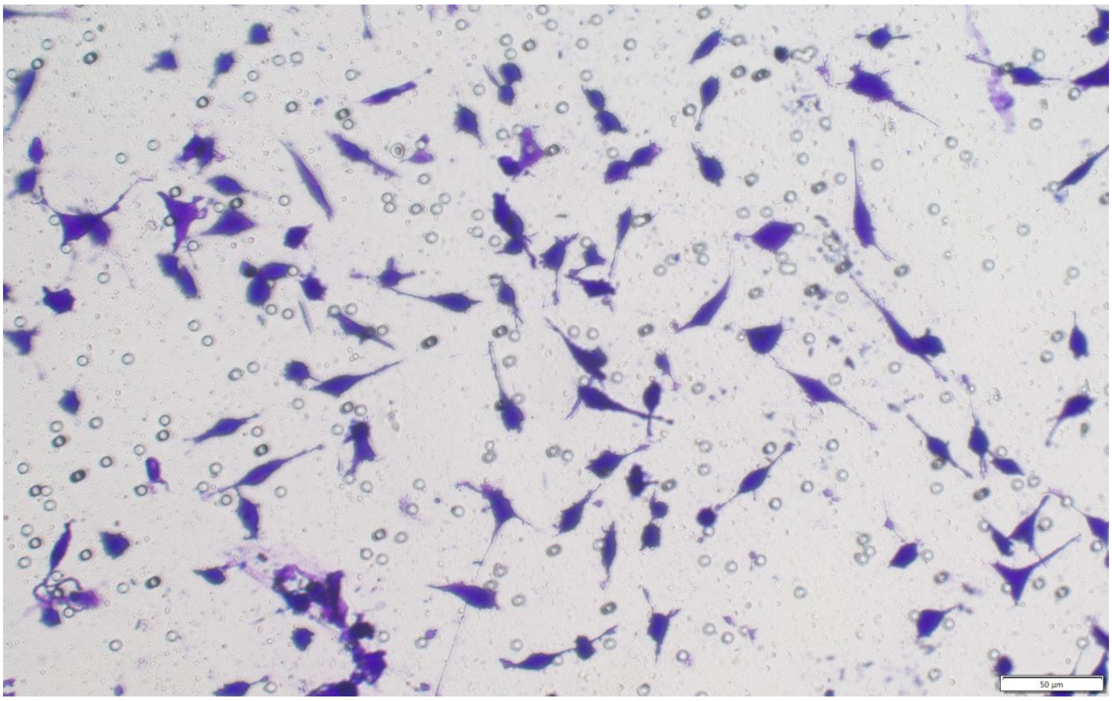
50 μm

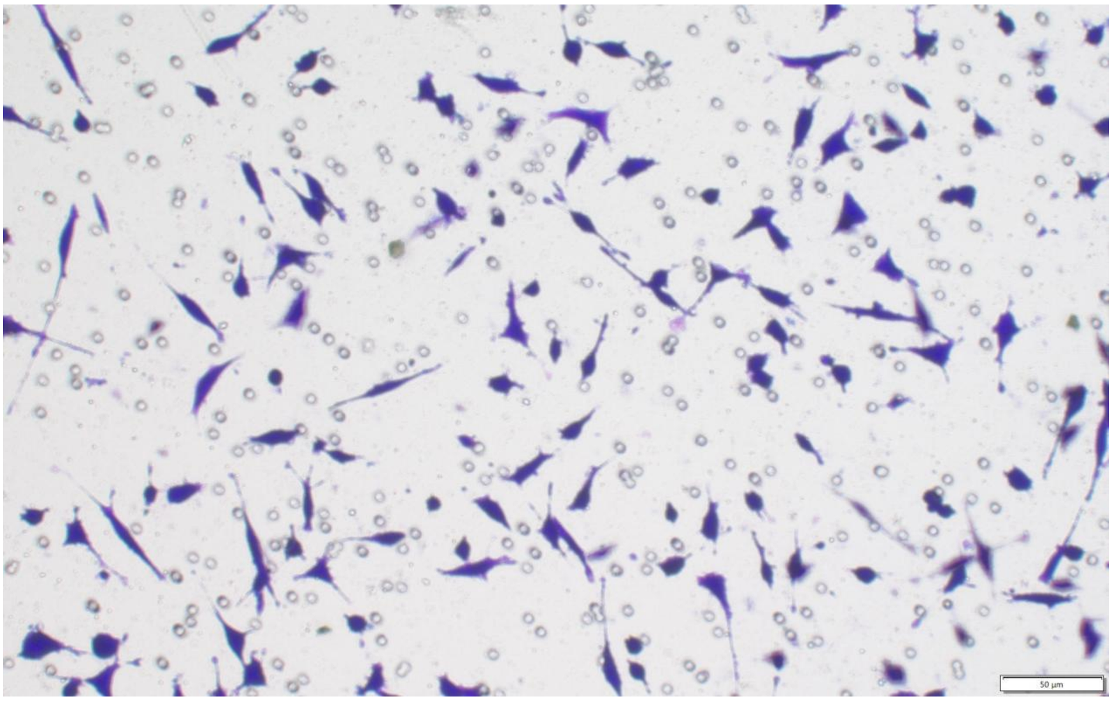
50 μm

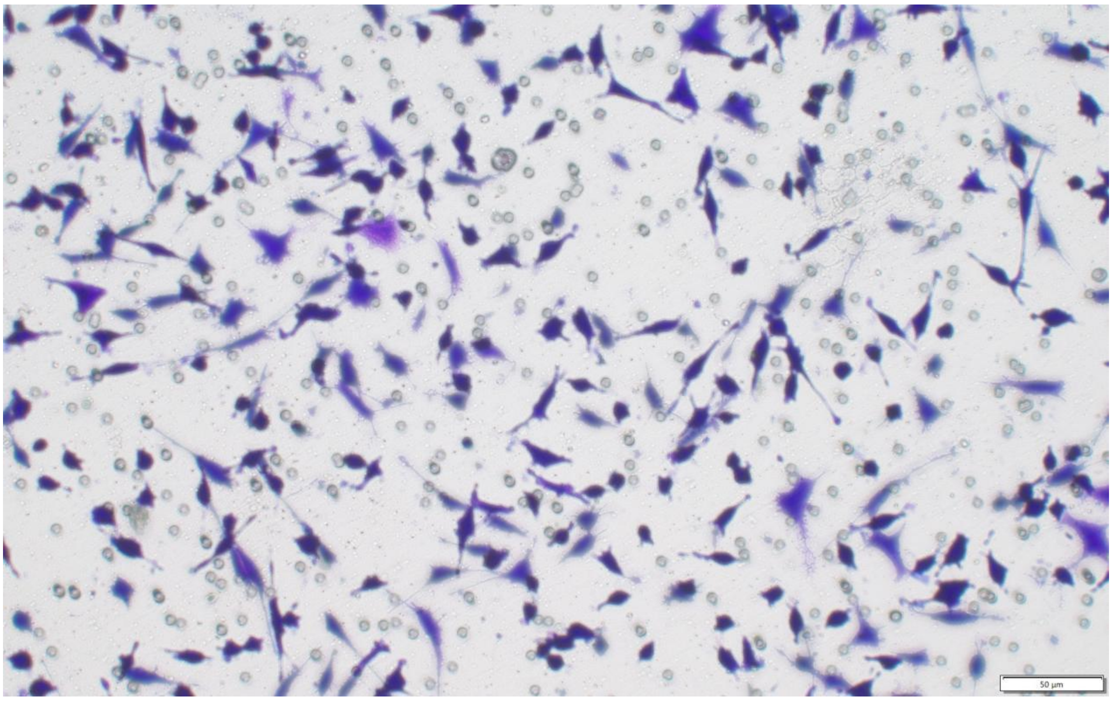
50 µm

# FIG10

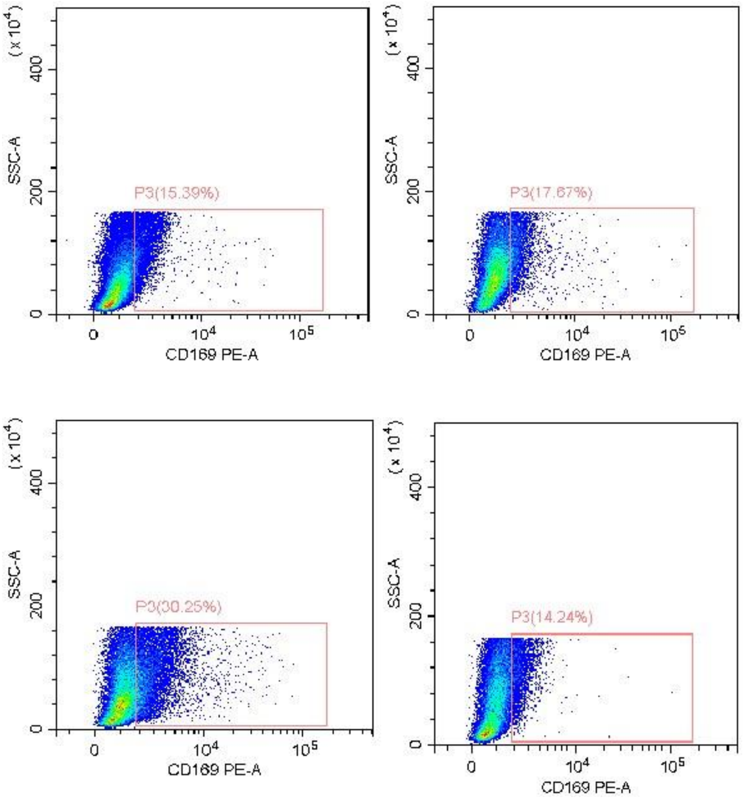
(x 10^4)
400
200
0
SSC-A
P3(15.39%)
0
10^4
10^5
CD169 PE-A
P3(17.67%)
P3(30.25%)
P3(14.24%)

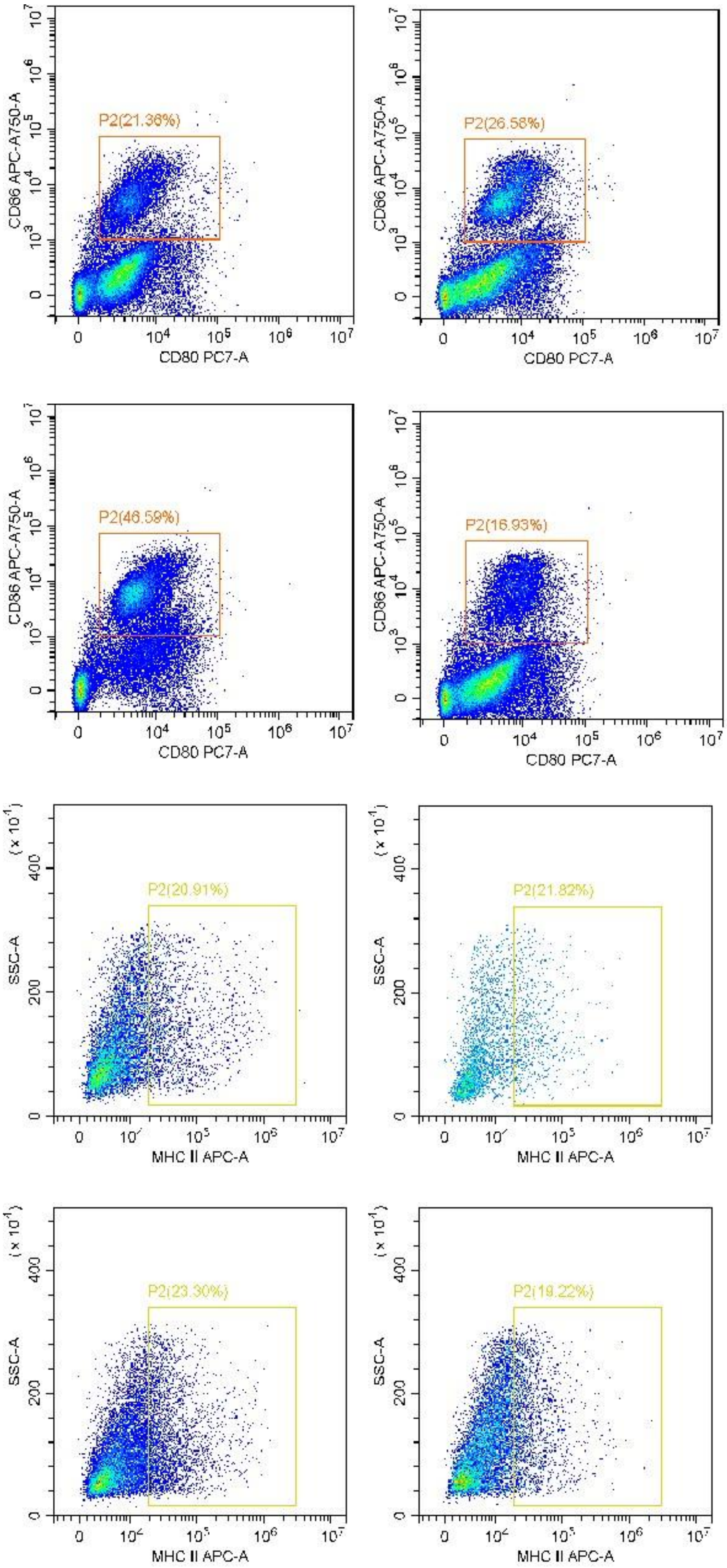
P2(21.36%)
CD86 APC-A750-A
CD80 PC7-A
P2(26.58%)
CD86 APC-A750-A
CD80 PC7-A
P2(46.59%)
CD86 APC-A750-A
CD80 PC7-A
P2(16.93%)
CD86 APC-A750-A
CD80 PC7-A
P2(20.91%)
SSC-A
MHC II APC-A
P2(21.82%)
SSC-A
MHC II APC-A
P2(23.30%)
SSC-A
MHC II APC-A
P2(19.22%)
SSC-A
MHC II APC-A

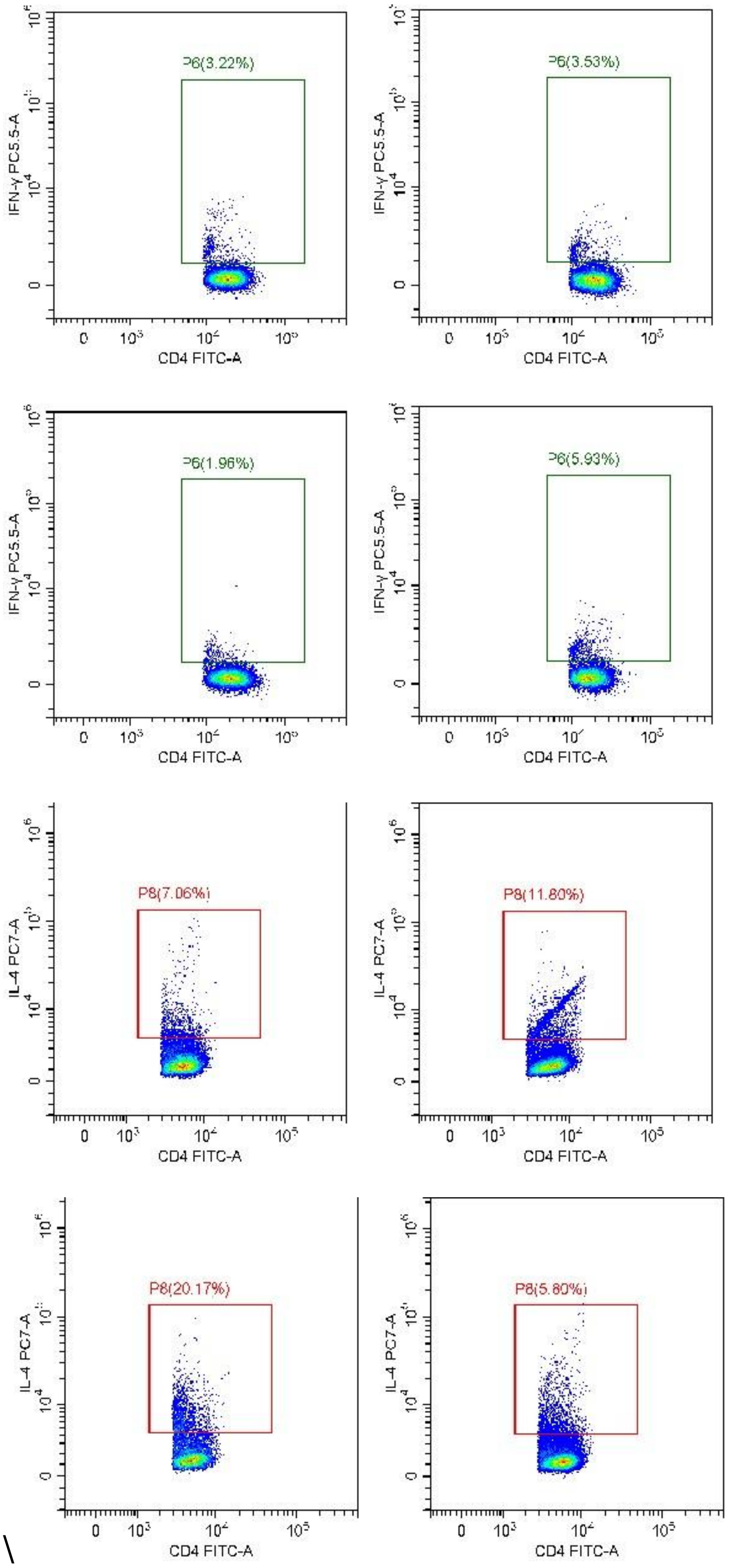
P6(3.22%)
IFN-γ PC5.5-A
CD4 FITC-A
P6(3.53%)
IFN-γ PC5.5-A
CD4 FITC-A
P6(1.96%)
IFN-γ PC5.5-A
CD4 FITC-A
P6(5.93%)
IFN-γ PC5.5-A
CD4 FITC-A
P8(7.06%)
IL-4 PC7-A
CD4 FITC-A
P8(11.80%)
IL-4 PC7-A
CD4 FITC-A
P8(20.17%)
IL-4 PC7-A
CD4 FITC-A
P8(5.80%)
IL-4 PC7-A
CD4 FITC-A

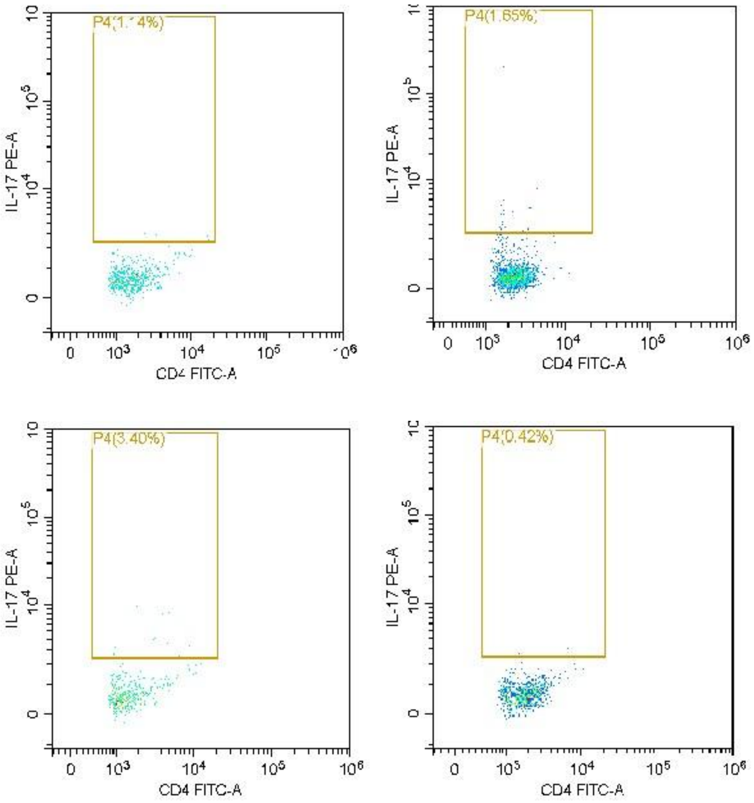
P4(1.65%)
P4(3.40%)
P4(0.42%)
IL-17 PE-A
CD4 FITC-A

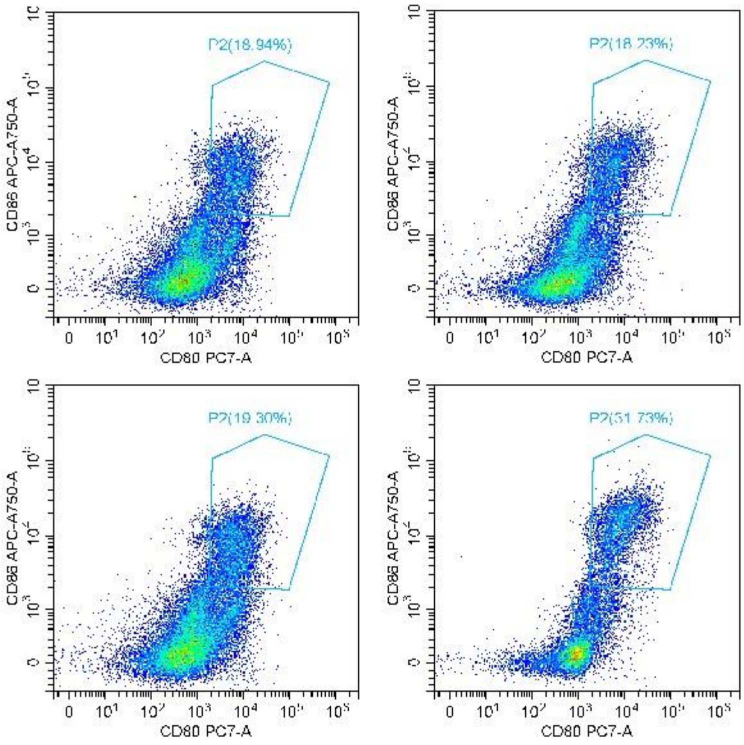
P2(18.94%)
P2(18.73%)
P2(19.30%)
CD86 APC-A750-A
CD80 PC7-A

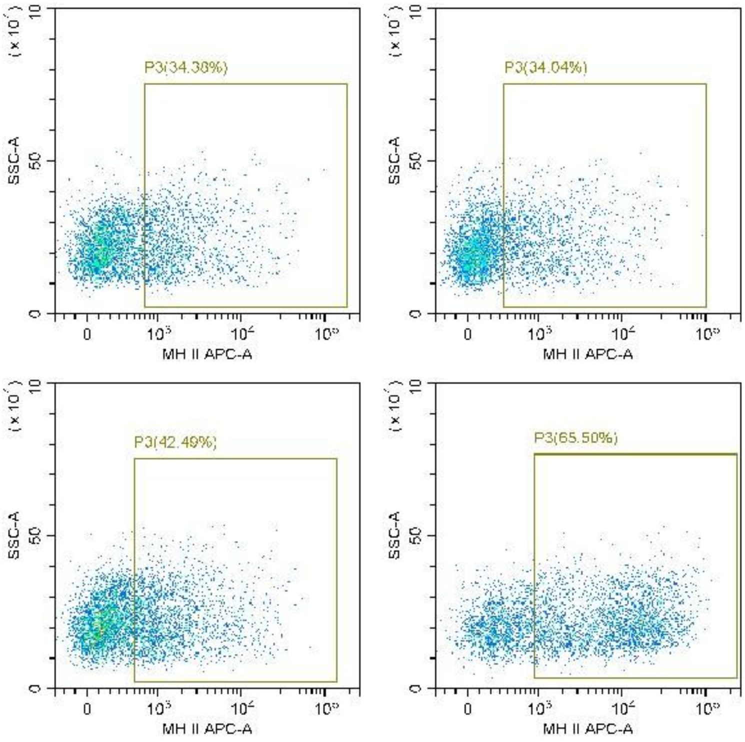


# Supplementary Materials

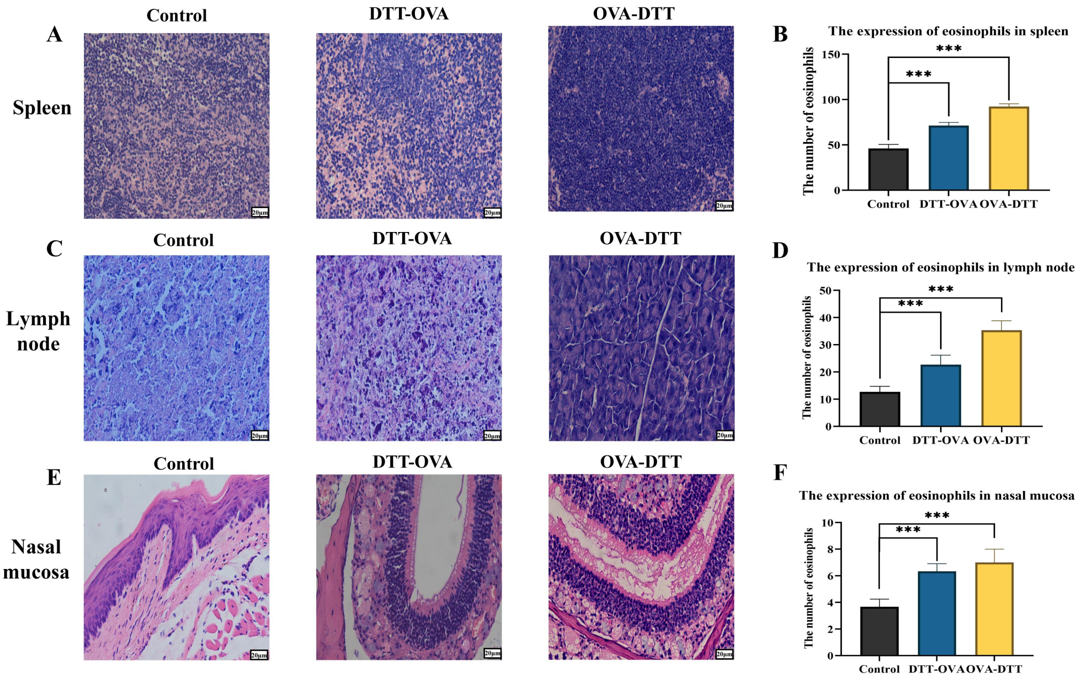

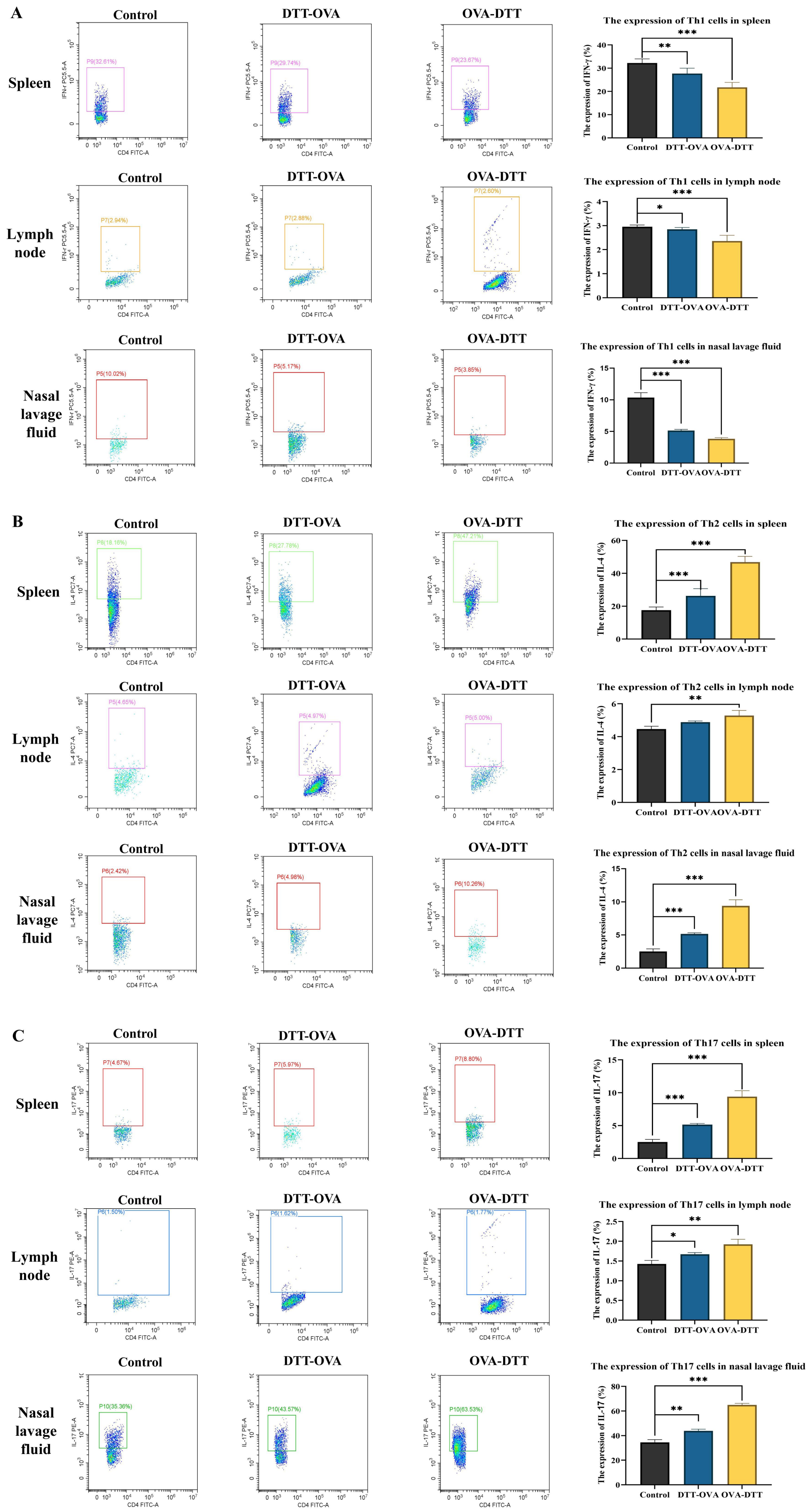

A
Control
DTT-OVA
OVA-DTT
Spleen
P9(32.61%)
P9(29.74%)
P9(23.67%)
The expression of Th1 cells in spleen
The expression of IFN-γ (%)
Lymph node
P7(2.94%)
P7(2.88%)
P7(2.60%)
The expression of Th1 cells in lymph node
Nasal lavage fluid
P5(10.02%)
P5(5.17%)
P5(3.85%)
The expression of Th1 cells in nasal lavage fluid
IFN-r PC5.5-A
CD4 FITC-A
B
Control
DTT-OVA
OVA-DTT
Spleen
P8(18.16%)
P8(27.78%)
P8(47.21%)
The expression of Th2 cells in spleen
The expression of IL-4 (%)
Lymph node
P5(4.65%)
P5(4.97%)
P5(5.00%)
The expression of Th2 cells in lymph node
Nasal lavage fluid
P6(2.42%)
P6(4.98%)
P6(10.26%)
The expression of Th2 cells in nasal lavage fluid
IL-4 PC7-A
CD4 FITC-A
C
Control
DTT-OVA
OVA-DTT
Spleen
P7(4.67%)
P7(5.97%)
P7(8.80%)
The expression of Th17 cells in spleen
The expression of IL-17 (%)
Lymph node
P6(1.50%)
P6(1.62%)
P6(1.77%)
The expression of Th17 cells in lymph node
Nasal lavage fluid
P10(35.36%)
P10(43.57%)
P10(63.53%)
The expression of Th17 cells in nasal lavage fluid
IL-17 PE-A
CD4 FITC-A

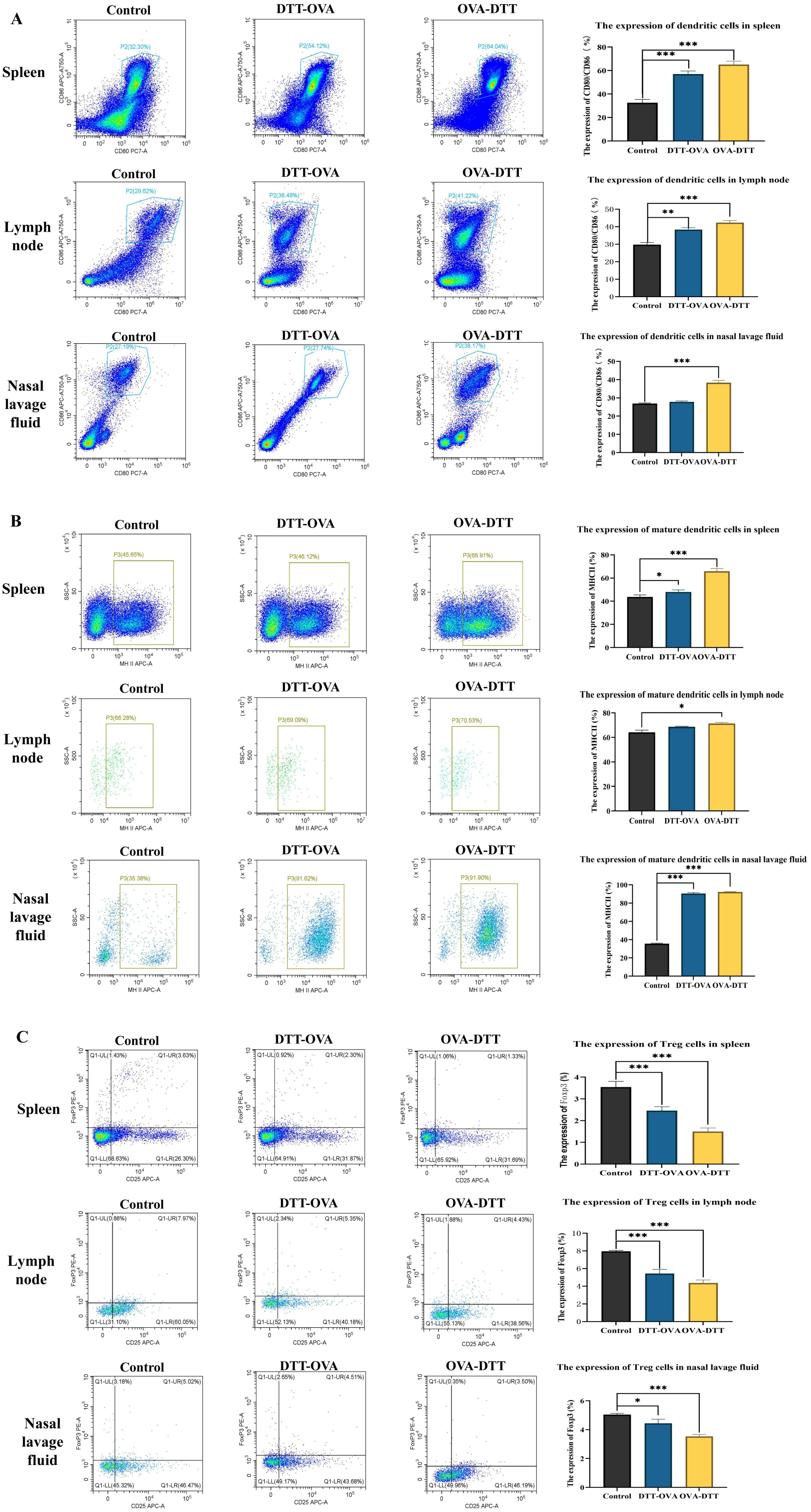
A
Control
DTT-OVA
OVA-DTT
Spleen
Lymph node
Nasal lavage fluid
CD86 APC-A750-A
CD80 PC7-A
The expression of dendritic cells in spleen
The expression of dendritic cells in lymph node
The expression of dendritic cells in nasal lavage fluid
The expression of CD80/CD86（%）
B
SSC-A
MH II APC-A
The expression of mature dendritic cells in spleen
The expression of mature dendritic cells in lymph node
The expression of mature dendritic cells in nasal lavage fluid
The expression of MHCII (%)
C
FoxP3 PE-A
CD25 APC-A
The expression of Treg cells in spleen
The expression of Treg cells in lymph node
The expression of Treg cells in nasal lavage fluid
The expression of Foxp3 (%)

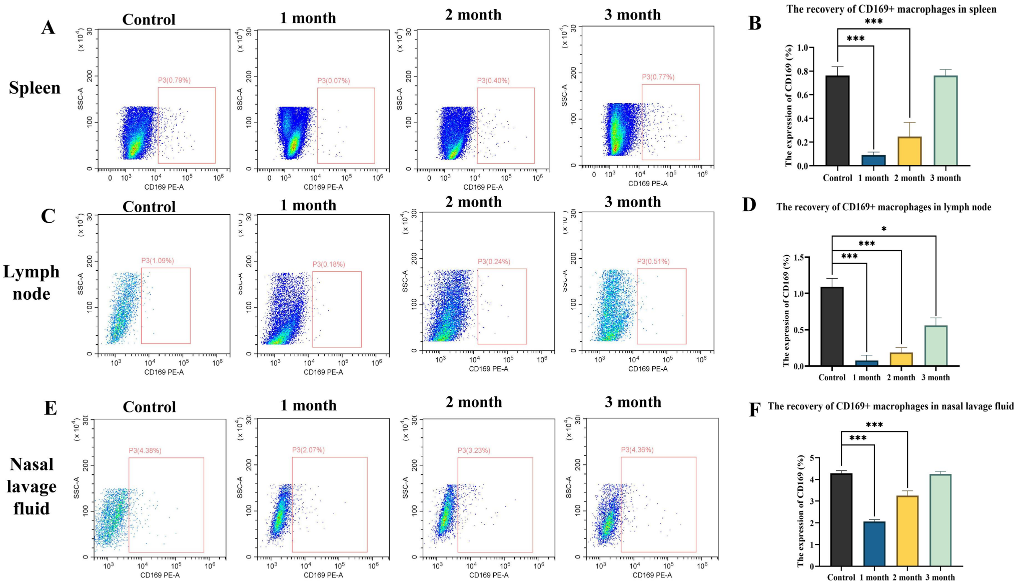
A
B
C
D
E
F
Control
1 month
2 month
3 month
Spleen
Lymph node
Nasal lavage fluid
SSC-A
CD169 PE-A
The recovery of CD169+ macrophages in spleen
The recovery of CD169+ macrophages in lymph node
The recovery of CD169+ macrophages in nasal lavage fluid
The expression of CD169 (%)
***
*

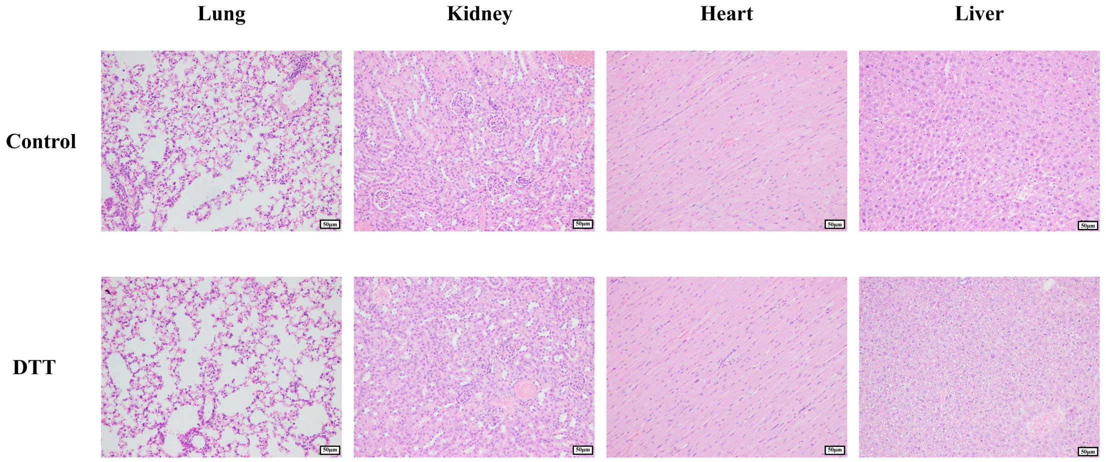
Lung
Kidney
Heart
Liver
Control
DTT
50μm
